# Design, synthesis, and discovery of novel oxindoles bearing 3-heterocycles as species-specific and combinatorial agents in eradicating *Staphylococcus* species

**DOI:** 10.1038/s41598-019-44304-1

**Published:** 2019-09-16

**Authors:** Jonghoon Shin, Krishna Bahadur Somai Magar, Jungwoon Lee, Kwang-sun Kim, Yong Rok Lee

**Affiliations:** 1 Department of Chemistry and Chemistry Institute for Functional Materials, Pusan National University, Busan, 46241 Republic of Korea; 2 School of Chemical Engineering, Yeungnam University, Gyeongsan, 38541 Republic of Korea; 3 Environmental Disease Research Center, Korea Research Institute of Bioscience & Biotechnology, 125 Gwahak-ro, Yuseong-gu, Daejeon 34141 Republic of Korea

**Keywords:** Chemical modification, Synthetic chemistry methodology

## Abstract

A series of new functionalized 3-indolylindolin-2-ones, 3-(1-methylpyrrol-2-yl)indolin-2-ones, and 3-(thiophen-2-yl)indolin-2-ones were synthesized by using novel indium (III)-catalysed reaction of various 3-diazoindolin-2-ones with indoles, 1-methylpyrrole, or thiophene *via* one-pot procedure. The newly synthesized compounds were characterized and screened for their *in vitro* antibacterial activity against various *Staphylococcus* species, including methicillin-resistant *Staphylococcus aureus*. results revealed that five compounds **KS15**, **KS16**, **KS17**,** KS19**, and **KS20** exhibited potent and specific antibacterial activity against *Staphylococcus* species albeit inactive against Gram-negative bacteria. Especially, compounds exhibited superior antibacterial potency against *Staphylococcus epidermidis* compared to the reference drug streptomycin. The most potential compound **KS16** also increased the susceptibility of *Staphylococcus aureus* to ciprofloxacin, gentamicin, kanamycin, and streptomycin. Among them, **KS16** was found to be a synergistic compound with gentamicin and kanamycin. Furthermore, the cellular level of autolysin protein was increased from the **KS16**-treated *Staphylococcus aureus* cells. Finally, *in vitro* CCK-8 assays showed that **KS16** exhibited no cytotoxicity at the minimum inhibitory concentrations used for killing *Staphylococcus* species. From all our results, novel oxindole compounds directly have lethal action or boost existing antibiotic power with the reduction of doses and toxicity in the treatment of multidrug-resistant *Staphylococcus* species.

## Introduction

The eruption of antimicrobial resistance (AMR) has become a serious health concern, making once-treatable pathogenic diseases deadly again and undermining the achievements of current medicine^1,2^. It is also expected that deaths attributable to AMR will exceed 10 million per annum by 2050, from a current baseline of 700,000 deaths annually^3^. Therefore, the development of new therapeutic materials and methods to eradicate AMR pathogens is in high demand to overcome the infections caused by such bacteria.

Among such pathogens, opportunistic human pathogens *Staphylococcus* species are the most abundant skin-colonizing Gram-positive bacteria and the most common cause of nosocomial infections and community-associated skin infections, which significantly complicate and increase the cost of medical treatment^4,5^. Among the *Staphylococcus* species, *Staphylococcus aureus* (*S*. *aureus*) has been the subject of most studies owing to its ability to cause severe and life-threatening diseases, such as severe sepsis, pneumonia, toxic shock syndrome, and endocarditis^6,7^. In addition to *S*. *aureus*, other *Staphylococcus* species are also highly detrimental to human health, such as *S*. *epidermidis*, one of the most frequent causes of nosocomial infections present on indwelling devices^8–10^ and *S*. *saprophyticus*, the second most important cause of urinary tract infections^11^. In particular, *S*. *aureus* and *S*. *epidermidis*, methicillin-resistant strains, are now common to hospitals and have spread in a pandemic fashion within the community, producing community-associated methicillin resistant *Staphylococcus* species^12,13^. Many different approaches have been tried to prevent or eradicate the infection of *Staphylococcus* species. One approach is that the development of promising vaccines against *S*. *aureus* species. However, there is no *S*. *aureus* vaccine on the market. There are main reasons why *S*. *aureus* vaccine development has made it to challenge^14^. First, the immune responses caused by *S*. *aureus* infection has not been clearly understood. Second, *S*. *aureus* also expresses a large array of virulence factors, resulting any one may not prove effective. Therefore, current trials are focused on multiple antigen preparations. Recently, it was hypothesized that the master mechanism of *S*. *aureus* counteracts may hinder the immunological responses, resulting in failure of target-oriented vaccine development^15^. Therefore, the understanding of such mechanisms are the key success for a developing vaccine for the future, but it is still remaining as a challenge. In other aspects, new potential anti-*Staphylococcus* compounds with their plausible action mechanism^16–19^ and therapeutics^19,20^ have been discovered in recent years, but there is still a high demand for highly potent compounds to specifically target AMR *Staphylococcus* species among bacterial mixtures. Biofilm, a surface‐associated bacterial community surrounded by a self‐produced extracellular matrix^21–25^ associated with resistance to antimicrobial agents^26,27^. Recently, new materials such as dihydrazone analogues against biofilm forming Gram-negative^28^ and benzodioxane midst piperazine decorated chitosan silver nanoparticles (BP*C@AgNPs) was controlled the MRSA biofilm^29^, still indole and its derivatives have been regarded as interesting heterocyclic antibacterial molecules against AMR bacteria owing to their promising potential to inhibit the formation of biofilm. In addition, the modification of indoles with diverse functional groups would change the efficiency of biofilm inhibition and the antibacterial specificity. For example, C-5/C-7-hydroxylated indoles were more efficient with respect to biofilm inhibition in *Escherichia coli* (*E. coli*)^30,31^. Moreover, various halogenated indoles, especially 5-iodoindole derivatives, potently inhibit biofilm formation of both *E*. *coli* and *S*. *aureus*^32^. Alternatively, oxindoles, which are structurally similar to indoles, have diverse pharmacological profiles^33–35^ and are the most recent synthetic class of new antibacterial agents. For example, a series of oxindole-based 3(*Z*)-{4-[4-(arylsulfonyl)piperazin-1-ylbenzylidene]-1,3-dihydro-2*H*-indol-2-one derivatives exhibited significant antibacterial activity against *S*. *aureus*, *Streptococcus pyrogenes*, *E*. *coli*, and *Pseudomonas* (*P*.) *aeruginosa*^36^. Moreover, several pharmacophores combined with a single heterocyclic indole or oxindole molecule can result to an enhanced antibacterial activity. For example, novel heterocyclic spiro-oxindole derivatives were active against *E*. *coli* and *S*. *aureus*, with minimum inhibitory concentrations (MIC) of 500 μg equivalent to that of the standard drug, streptomycin^37^. One study reported that a combination of spiro-oxindole, 2-amino-4*H*-pyran, and 1,2,3-triazoles in a single matrix showed good Gram-positive antibacterial activity against *S*. *aureus* (MIC = 32–256 μg/mL); however, none of the compounds affected Gram-negative bacteria (*E*. *coli* and *P*. *aeruginosa*)^38^. In addition, indole derivatives containing heterocyclic moieties synthesized by using 3-chloro-1*H*-indole-2-carbaldehyde have shown excellent antibacterial activity^39^. Based on these previous reports, the antibacterial activity of oxindole derivatives has been observed to enhance and alter for specificity through different functionalization and structural modifications. Therefore, it is of great interest to synthesize and identify highly efficient and selective oxindoles bearing pharmacologically active heterocycles at the 3-position, such as pyrrole, thiophene, and indole moieties, that are probable novel antibacterial agents against health-threatening *Staphylococcus* species.

**Figure 1:**
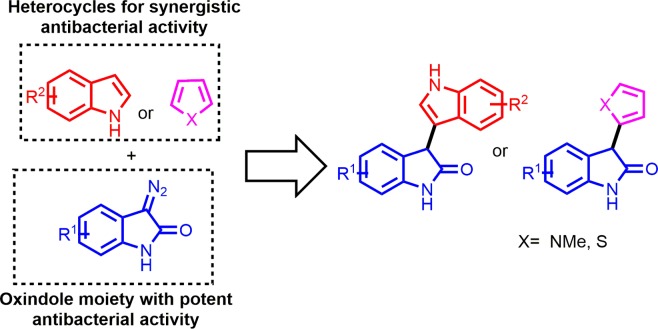
Design strategy for the novel heterocycle-bearing oxindoles. Chemical structures were drawn by ChemDraw Pro 13.0 Suite (PerkinElemer, USA).

Based on the above considerations, we have designed and synthesized several different C-3-functionalized oxindoles bearing indole, pyrrole, and thiophene moieties by a novel indium(III)-catalysed reaction (Fig. 1). In addition, we also screened the synthetic compounds for their anti-*Staphylococcus* activity and synergistic action to existing antibiotics. A preliminary studies of structure-activity relationship (SAR) for the synthesized compounds have also been performed.

## Methods

### Materials

All materials were obtained from commercial suppliers and were used without further purification.

### Synthesis of 3-(1H-indol-3-yl)indolin-2-ones KS1 to KS12

**Figure 2:**
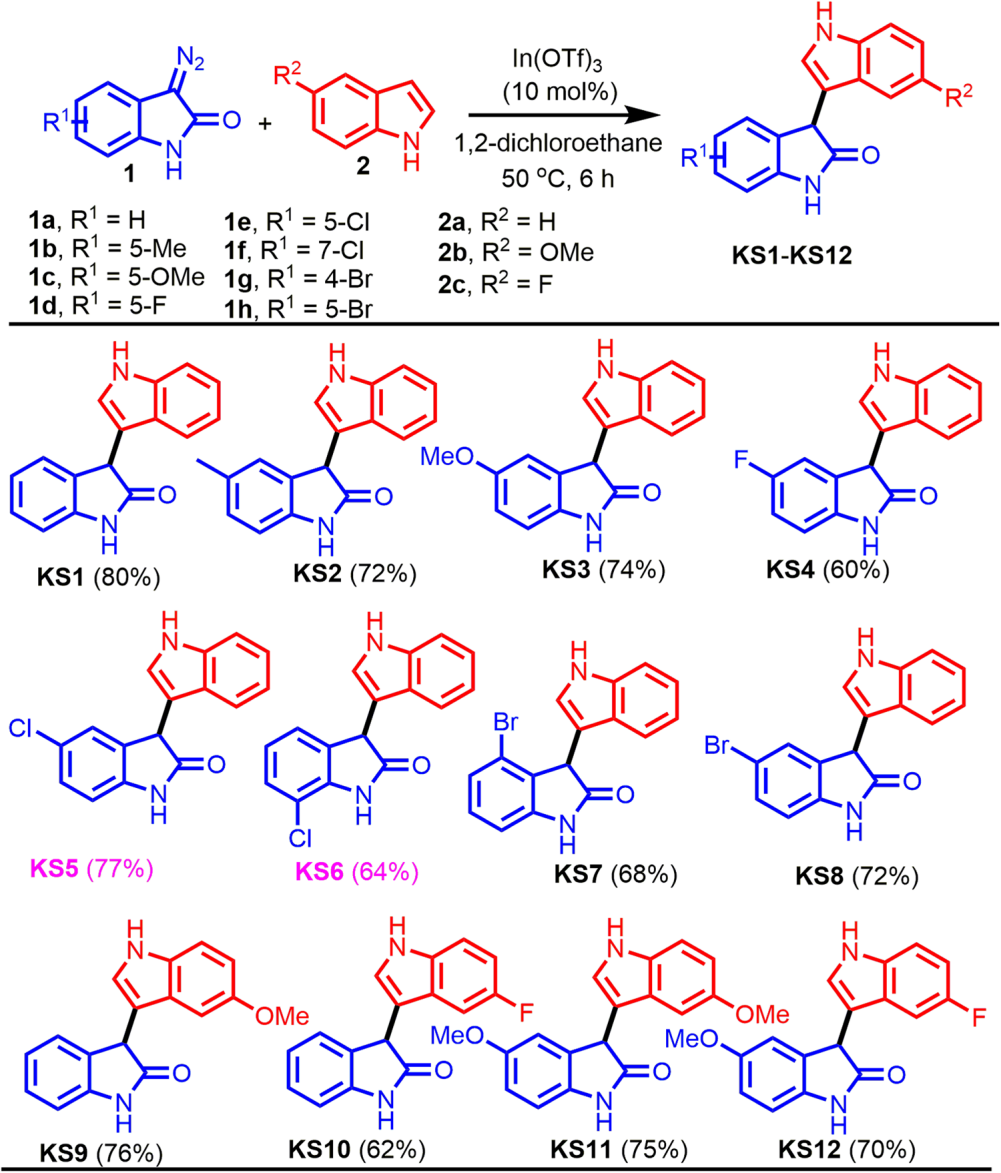
One-pot synthesis of 3-indolylindolin-2-ones **KS1**-**KS12**. Chemical structures were drawn by ChemDraw Pro 13.0 Suite (PerkinElemer, USA).

A reaction mixture of the corresponding 3-diazoindolin-2-ones (1, 0.5 mmol) and indoles (2, 0.55 mmol) in 1,2-dichloroethane was stirred at 50 °C for 6 h in the presence of 10 mol% In(OTf)_3_ (Fig. 2). After completion of the reaction, as indicated by TLC, the reaction mixture was evaporated in a rotary evaporator and the residue was purified by silica gel column chromatography using hexane/ethyl acetate as the eluent to afford the desired products **KS1** to **KS12**.

### Synthesis of 3-(1-methyl-1H-pyrrol-2-yl)indolin-2-ones KS13 to KS17 and 3-(thiophen-2-yl)indolin-2-ones KS18 to KS20

**Figure 3:**
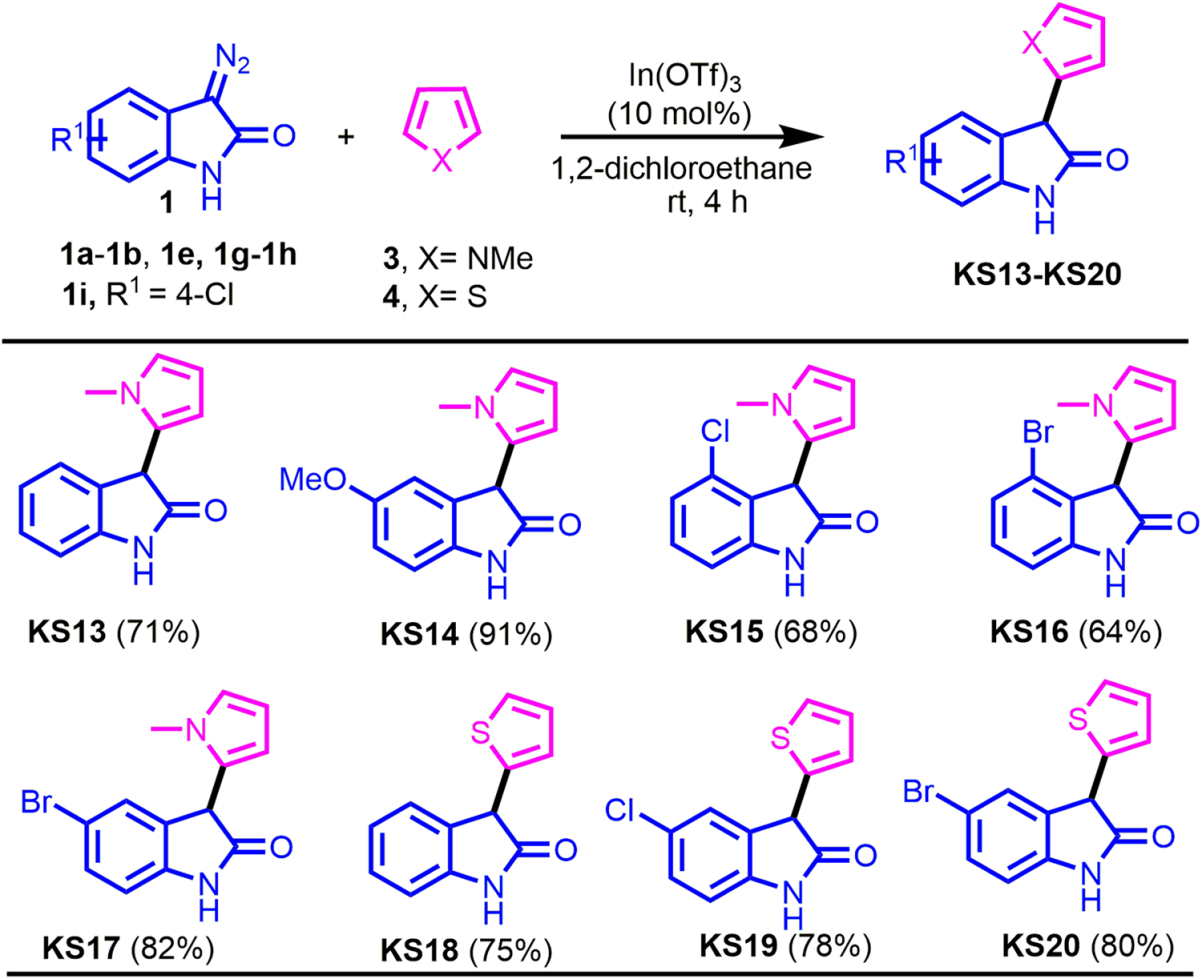
Synthesis of 3-(1-methylpyrrol-2-yl)indolin-2-ones **KS13**-**KS17** and 3-(thiophen-2-yl)indolin-2-ones **KS18**-**KS20**. Chemical structures were drawn by ChemDraw Pro 13.0 Suite (PerkinElemer, USA).

A reaction mixture of the corresponding 3-diazoindolin-2-ones (**1**, 0.5 mmol) and 1-methylpyrrole (**3**, 0.55 mmol) or thiophene (**4**, 0.55 mmol) in 1,2-dichloroethane was stirred at room temperature for 4 h in the presence of 10 mol% In(OTf)_3_ (Fig. 3). After completion of the reaction, as indicated by TLC, the reaction mixture was evaporated in a rotary evaporator and the residue was purified by silica gel column chromatography using hexane/ethyl acetate as the eluent to afford the desired products **KS13** to **KS17** and **KS18** to **KS20**.

## Characterization

All melting point (MP) was obtained on a Büchi Melting Point B-540 apparatus (Büchi Labortechnik, Switzerland). Mass spectra (MS) were taken in ESI mode on Agilent 1100 LC-MS (Agilent, USA). ^1^H NMR and ^13^C NMR spectra were recorded on Bruker ARX-400, 400 MHz or Bruker ARX-600, 600 MHz spectrometers (Bruker Bioscience, USA) with TMS as an internal standard. Reaction time of the products were monitored by TLC on FLUKA silica gel aluminium cards (0.2 mm thickness) with fluorescent indicator at 254 nm. Column chromatography was run on silica gel. The purity of all the synthesized compounds, determined by their ^1^H and ^13^C NMR spectra, was observed to be 95–99% (see Supporting Information, SI1 for details). In addition, the purity of compound **KS16** was determined by Gas Chromatography (GC) and was found to be 99% (see SI2 for details).

### 3-(1H-Indol-3-yl)indolin-2-one (KS1)

Yield 80% (99 mg), as a red solid: MP 140–142 °C; IR (attenuated total reflectance (ATR)) ν_max_/cm^−1^ 3271, 1700, 1616, 1465, 1329, 1246, 1095, 1042, 805, 740, 600, and 550; ^1^H NMR (600 MHz, DMSO-d_6_) δ 11.02 (1H, s), 10.56 (1H, s), 7.37 (1H, d, *J* = 8.4 Hz), 7.28–7.27 (1H, m), 7.22–7.19 (1H, m), 7.06–7.02 (3H, m), 6.95 (1H, d, *J* = 7.2 Hz), 6.91–6.84 (2H, m), 5.01 (1H, s); ^13^C NMR (150 MHz, DMSO-d_6_) δ 177.7, 142.5, 136.5, 130.4, 127.8, 126.7, 126.0, 124.4, 121.4, 121.1, 118.5, 111.6, 110.0, 109.3, 109.2, 44.4; HRMS m/z (M^+^) calculated for C_16_H_12_N_2_O: 248.0950. Found: 248.0951.

### 3-(1H-Indol-3-yl)-5-methylindolin-2-one (KS2)

Yield 72% (94 mg), as a white solid: MP 195–197 °C; IR (ATR) ν_max_/cm^−1^ 3400, 3165, 1691, 1600, 1488, 1450, 1266, 1106, 1020, and 745; ^1^H NMR (600 MHz, DMSO-d_6_) δ 11.00 (1H, s), 10.44 (1H, s), 7.36 (1H, d, *J* = 7.8 Hz), 7.28 (1H, d, *J* = 2.4 Hz), 7.04–7.02 (2H, m), 7.00 (1H, d, *J* = 8.4 Hz), 6.87–6.85 (1H, m), 6.84–6.82 (2H, m), 4.86 (1H, s), 2.16 (3H, s); ^13^C NMR (150 MHz, DMSO-d_6_) δ 177.7, 140.0, 136.5, 130.5, 130.2, 128.0, 126.0, 125.0, 124.5, 121.1, 118.6, 118.5, 111.5, 110.1, 109.0, 44.4, 20.6; HRMS m/z (M^+^) calculated for C_17_H_14_N_2_O: 262.1106. Found: 262.1108.

### 3-(1H-Indol-3-yl)-5-methoxyindolin-2-one (KS3)

Yield 74% (103 mg), as a yellow solid: MP 205–207 °C; IR (ATR) ν_max_/cm^−1^ 3402, 3169, 2959, 1689, 1605, 1486, 1454, 1306, 1263, 1206, 1100, 1024, 747, 614, 585, and 518; ^1^H NMR (600 MHz, DMSO-d_6_) δ 11.01 (1H, s), 10.37 (1H, s), 7.36 (1H, d, *J* = 8.4 Hz), 7.28 (1H, d, *J* = 1.8 Hz), 7.05–7.03 (2H, m), 6.87–6.85 (2H, m), 6.78 (1H, dd, *J* = 8.4, 1.8 Hz), 6.62 (1H, s), 4.88 (1H, s), 3.61 (3H, s); ^13^C NMR (150 MHz, DMSO-d_6_) δ 177.5, 154.7, 136.5, 136.0, 131.7, 126.0, 124.5, 124.4, 121.1, 118.5, 112.5, 111.4, 111.3, 110.0, 109.5, 55.3, 45.0; HRMS m/z (M^+^) calculated for C_17_H_14_N_2_O_2_: 278.1055. Found: 278.1054.

### 5-Fluoro-3-(1H-indol-3-yl)indolin-2-one (KS4)

Yield 60% (80 mg), as a white solid: MP 158–160 °C; IR (ATR) ν_max_/cm^−1^ 3318, 2954, 1708, 1612, 1482, 1394, 1258, 1179, 1101, 1015, 743, 692, and 590; ^1^H NMR (600 MHz, acetone-d_6_) δ 10.22 (1H, s), 9.55 (1H, s), 7.39 (1H, d, *J* = 8.4 Hz), 7.29 (1H, d, *J* = 2.4 Hz), 7.24 (1H, d, *J* = 8.4 Hz), 7.07 (1H, t, *J* = 7.8 Hz), 7.00–6.99 (2H, m), 6.93–6.90 (2H, m), 5.00 (1H, s); ^13^C NMR (150 MHz, acetone-d_6_) δ 178.0, 159.5 (d, *J* = 235.8 Hz), 139.6, 138.0, 133.3 (d, *J* = 8.1 Hz), 127.2, 125.0, 122.4, 119.7 (d, *J* = 5.7 Hz), 114.8 (d, *J* = 23.1 Hz), 113.1 (d, *J* = 25.2 Hz), 112.4, 112.3, 111.1, 110.7 (d, *J* = 8.1 Hz), 45.8; HRMS m/z (M^+^) calculated for C_16_H_11_FN_2_O: 266.0855. Found: 266.0854.

### 5-Chloro-3-(1H-indol-3-yl)indolin-2-one (KS5)

Yield 64% (90 mg), as a white solid: MP 170–172 °C; IR (ATR) ν_max_/cm^−1^ 3360, 3130, 1694, 1612, 1459, 1325, 1220, 1136, 1042, 925, and 732; ^1^H NMR (600 MHz, DMSO-d_6_) δ 11.05 (1H, s), 10.67 (1H, s), 7.37 (1H, d, *J* = 7.8 Hz), 7.30 (1H, d, *J* = 2.4 Hz), 7.26 (1H, d, *J* = 8.4 Hz), 7.06 (1H, t, *J* = 7.8 Hz), 7.00 (1H, d, *J* = 8.4 Hz), 6.99 (1H, s), 6.94 (1H, d, *J* = 9.0 Hz), 6.87 (1H, t, *J* = 7.8 Hz), 4.97 (1H, s); ^13^C NMR (150 MHz, DMSO-d_6_) δ 177.3, 141.4, 136.4, 132.6, 127.6, 125.7, 125.4, 124.7, 124.3, 121.2, 118.6, 118.2, 111.7, 110.6, 109.2, 44.4; HRMS m/z (M^+^) calculated for C_16_H_11_ClN_2_O: 282.0560. Found: 282.0562.

### 7-Chloro-3-(1H-indol-3-yl)indolin-2-one (KS6)

Yield 77% (109 mg), as a yellowish solid: MP 213–215 °C; IR (ATR) ν_max_/cm^−1^ 3369, 3133, 1697, 1616, 1455, 1322, 1224, 1133, 1098, 1047, 928, 739, 636, and 539; ^1^H NMR (600 MHz, DMSO-d_6_) δ 11.06 (1H, s), 10.97 (1H, s), 7.38 (1H, d, *J* = 8.4 Hz), 7.30 (1H, d, *J* = 1.2 Hz), 7.27 (1H, d, *J* = 7.8 Hz), 7.07–7.03 (2H, m), 6.97 (1H, d, *J* = 7.2 Hz), 6.92 (1H, d, *J* = 8.4 Hz), 6.90–6.87 (1H, m), 5.07 (1H, s); ^13^C NMR (150 MHz, DMSO-d_6_) δ 177.5, 140.2, 136.4, 132.2, 127.7, 125.8, 124.6, 123.0, 122.7, 121.2, 118.6, 118.3, 113.5, 111.6, 109.3, 45.1; HRMS m/z (M^+^) calculated for C_16_H_11_ClN_2_O: 282.0560. Found: 282.0561.

### 4-Bromo-3-(1H-indol-3-yl)indolin-2-one (KS7)

Yield 68% (111 mg), as a white solid: MP 216–218 °C; IR (ATR) ν_max_/cm^−1^ 3282, 3115, 3056, 1695, 1607, 1438, 1373, 1243, 1102, 1043, 733, 640, and 563; ^1^H NMR (600 MHz, DMSO-d_6_) δ 11.01 (1H, s), 10.73 (1H, s), 7.35 (1H, d, *J* = 7.8 Hz), 7.27 (1H, d, *J* = 2.4 Hz), 7.19 (1H, t, *J* = 7.8 Hz), 7.07 (1H, d, *J* = 7.8 Hz), 7.04–7.01 (1H, m), 6.97–6.93 (2H, m), 6.83 (1H, t, *J* = 7.2 Hz), 4.88 (1H, s); ^13^C NMR (150 MHz, DMSO-d_6_) δ 176.5, 144.6, 136.3, 130.0, 129.1, 125.8, 125.4, 124.8, 121.0, 119.2, 118.5, 118.1, 111.6, 108.5, 107.7, 45.8; HRMS m/z (M^+^) calculated for C_16_H_11_BrN_2_O: 326.0055. Found: 326.0057.

### 5-Bromo-3-(1H-indol-3-yl)indolin-2-one (KS8)

Yield 72% (118 mg), as a white solid: MP 202–204 °C; IR (ATR) ν_max_/cm^−1^ 3280, 3112, 3050, 1698, 1600, 1431, 1378, 1243, 1042, and 730; ^1^H NMR (600 MHz, DMSO-d_6_) δ 11.05 (1H, s), 10.68 (1H, s), 7.39–7.36 (2H, m), 7.30 (1H, d, *J* = 2.4 Hz), 7.10 (1H, s), 7.05 (1H, t, *J* = 7.8 Hz), 7.01 (1H, d, *J* = 7.8 Hz), 6.91–6.87 (2H, m), 4.98 (1H, s); ^13^C NMR (150 MHz, DMSO-d_6_) δ 177.2, 144.8, 136.4, 133.1, 130.4, 127.0, 125.7, 124.7, 121.2, 118.7, 118.2, 113.1, 111.7, 111.2, 109.2, 44.4; HRMS m/z (M^+^) calculated for C_16_H_11_BrN_2_O: 326.0055. Found: 326.0052.

### 3-(5-Methoxy-1H-indol-3-yl)indolin-2-one (KS9)

Yield 76% (106 mg), as a white solid: MP 146–148 °C; IR (ATR) ν_max_/cm^−1^ 3201, 2921, 1697, 1616, 1466, 1271, 1211, 1021, 802, 749, and 678; ^1^H NMR (600 MHz, DMSO-d_6_) δ 10.84 (1H, s), 10.52 (1H, s), 7.25 (1H, d, *J* = 9.0 Hz), 7.21 (1H, t, *J* = 7.8 Hz), 7.18 (1H, d, *J* = 1.8 Hz), 7.03 (1H, d, *J* = 6.6 Hz), 6.93 (1H, d, *J* = 7.8 Hz), 6.90 (1H, t, *J* = 6.6 Hz), 6.71 (1H, dd, *J* = 9.0, 1.8 Hz), 6.54 (1H, d, *J* = 1.2 Hz), 4.87 (1H, s), 3.60 (3H, s); ^13^C NMR (150 MHz, DMSO-d_6_) δ 177.7, 152.8, 142.6, 131.6, 130.3, 127.7, 126.4, 124.9, 124.4, 121.4, 112.1, 110.7, 109.6, 109.1, 101.0, 55.1, 44.2; HRMS m/z (M^+^) calculated for C_17_H_14_N_2_O_2_: 278.1055. Found: 278.1054.

### 3-(5-Fluoro-1H-indol-3-yl)indolin-2-one (KS10)

Yield 62% (83 mg), as a yellowish solid: MP 152–154 °C; IR (ATR) ν_max_/cm^−1^ 3291, 2921, 2853, 1696, 1620, 1465, 1292, 1230, 1180, 1098, 935, 797, 747, 680, and 596; ^1^H NMR (600 MHz, DMSO-d_6_) δ 11.12 (1H, s), 10.51 (1H, s), 7.36 (1H, dd, *J* = 8.4, 4.2 Hz), 7.31 (1H, s), 7.22 (1H, t, *J* = 7.2 Hz), 7.04 (1H, d, *J* = 7.2 Hz), 6.95–6.93 (1H, m), 6.92–6.88 (2H, m), 6.75 (1H, d, *J* = 8.4 Hz), 5.01 (1H, s); ^13^C NMR (150 MHz, DMSO-d_6_) δ 177.4, 156.5 (d, *J* = 229.8 Hz), 142.5, 133.0, 130.0, 127.8, 126.2, 126.1 (d, *J* = 10.3 Hz), 124.4, 121.4, 112.5 (d, *J* = 9.1 Hz), 110.2 (d, *J* = 4.6 Hz), 109.3, 109.2 (d, *J* = 26.5 Hz), 103.0 (d, *J* = 22.9 Hz), 44.1; HRMS m/z (M^+^) calculated for C_16_H_11_FN_2_O: 266.0855. Found: 266.0856.

### 5-Methoxy-3-(5-methoxy-1H-indol-3-yl)indolin-2-one (KS11)

Yield 75% (115 mg), as a yellowish solid: MP 208–210 °C; IR (ATR) ν_max_/cm^−1^ 3256, 2924, 1698, 1489, 1280, 1200, 1024, 800, and 605; ^1^H NMR (600 MHz, DMSO-d_6_) δ 10.84 (1H, s), 10.34 (1H, s), 7.25 (1H, d, *J* = 9.0 Hz), 7.19 (1H, d, *J* = 2.4 Hz), 6.85–6.84 (1H, m), 6.78 (1H, dd, *J* = 8.4, 2.4 Hz), 6.72 (1H, dd, *J* = 8.4, 1.8 Hz), 6.63 (1H, s), 6.55 (1H, d, *J* = 1.8 Hz), 4.84 (1H, s), 3.62 (3H, s), 3.60 (3H, s); ^13^C NMR (150 MHz, DMSO-d_6_) δ 177.5, 154.7, 152.8, 136.1, 131.7, 131.6, 126.4, 125.0, 112.5, 112.1, 111.3, 110.7, 109.7, 109.5, 101.0, 55.3, 55.2, 40.0; HRMS m/z (M^+^) calculated for C_18_H_16_N_2_O_3_: 308.1161. Found: 308.1162.

### 3-(5-Fluoro-1H-indol-3-yl)-5-methoxyindolin-2-one (KS12)

Yield 70% (104 mg), as a yellowish solid: MP 214–216 °C; IR (ATR) ν_max_/cm^−1^ 3414, 3171, 2931, 1689, 1477, 1262, 1200, 1116, 1025, 779, 602, and 509; ^1^H NMR (600 MHz, DMSO-d_6_) δ 11.13 (1H, s), 10.38 (1H, s), 7.37 (1H, dd, *J* = 9.0, 4.2 Hz), 7.34 (1H, d, *J* = 2.4 Hz), 6.92–6.86 (2H, m), 6.81 (1H, d, *J* = 2.4 Hz), 6.79–6.76 (1H, m), 6.66 (1H, s), 4.89 (1H, s), 3.62 (3H, s); ^13^C NMR (150 MHz, DMSO-d_6_) δ 177.4, 158.1 (d, *J* = 229.9 Hz), 154.9, 136.0, 133.2, 131.4, 126.5, 126.2 (d, *J* = 9.9 Hz), 112.8, 112.6, 111.4, 110.3 (d, *J* = 4.7 Hz), 109.7, 109.4 (d, *J* = 25.8 Hz), 103.2 (d, *J* = 23.1Hz), 55.4, 44.7; HRMS m/z (M^+^) calculated for C_17_H_13_FN_2_O_2_: 296.0961. Found: 296.0958.

### 3-(1-Methyl-1H-pyrrol-2-yl)indolin-2-one (KS13)

Yield 71% (75 mg), as a brownish solid: MP 130–132 °C; IR (ATR) ν_max_/cm^−1^ 3075, 2927, 1703, 1611, 1467, 1315, 1157, 1088, 870, 711, and 547; ^1^H NMR (600 MHz, CDCl_3_) δ 9.07 (1H, s), 7.15 (1H, t, *J* = 7.2 Hz), 7.11 (1H, d, *J* = 7.8 Hz), 6.95 (1H, t, *J* = 7.8 Hz), 6.83 (1H, d, *J* = 7.8 Hz), 6.54 (1H, br s), 5.98 (1H, t, *J* = 3.0 Hz), 5.81 (1H, br s), 4.68 (1H, s), 3.50 (3H, s); ^13^C NMR (150 MHz, CDCl_3_) δ 177.8, 141.4, 129.5, 128.4, 128.2, 125.1, 123.6, 122.6, 110.1, 109.0, 107.0, 45.4, 34.2; HRMS m/z (M^+^) calculated for C_13_H_12_N_2_O: 212.0950. Found: 212.0947.

### 5-Methoxy-3-(1-methyl-1H-pyrrol-2-yl)indolin-2-one (KS14)

Yield 91% (110 mg), as a white solid: MP 204–206 °C; IR (ATR) ν_max_/cm^−1^ 3200, 2990, 1692, 1612, 1480, 1315, 1092, and 806; ^1^H NMR (600 MHz, DMSO-d_6_) δ 10.32 (1H, s), 6.82–6.78 (2H, m), 6.69–6.68 (2H, m), 5.89 (1H, t, *J* = 3.0 Hz), 5.62 (1H, br s), 4.92 (1H, s), 3.55 (3H, s), 3.35 (3H, s); ^13^C NMR (150 MHz, DMSO-d_6_) δ 176.2, 154.8, 135.8, 130.0, 127.1, 123.0, 113.0, 111.4, 109.7, 107.4, 106.3, 55.4, 45.0, 33.7; HRMS m/z (M^+^) calculated for C_14_H_14_N_2_O_2_: 242.1055. Found: 242.1056.

### 4-Chloro-3-(1-methyl-1H-pyrrol-2-yl)indolin-2-one (KS15)

Yield 68% (84 mg), as a white solid: MP 116–118 °C; IR (ATR) ν_max_/cm^−1^ 3194, 2903, 2805, 1689, 1625, 1480, 1315, 1149, 1082, and 812; ^1^H NMR (600 MHz, DMSO-d_6_) δ 10.66 (1H, s), 7.25 (1H, t, *J* = 7.8 Hz), 6.98 (1H, d, *J* = 8.4 Hz), 6.86 (1H, d, *J* = 7.2 Hz), 6.67 (1H, br s), 5.87 (1H, t, *J* = 3.0 Hz), 5.52 (1H, br s), 4.99 (1H, s), 3.59 (3H, s); ^13^C NMR (150 MHz, DMSO-d_6_) δ 175.4, 144.4, 130.0, 129.8, 126.2, 125.0, 122.7, 122.0, 108.2, 107.0, 106.4, 44.2, 33.7; HRMS m/z (M^+^) calculated for C_13_H_11_ClN_2_O: 246.0560. Found: 246.0562.

### 4-Bromo-3-(1-methyl-1H-pyrrol-2-yl)indolin-2-one (KS16)

Yield 64% (93 mg), as a white solid: MP 208–210 °C; IR (ATR) ν_max_/cm^−1^ 3160, 2912, 2855, 1695, 1621, 1487, 1313, 1152, 1092, and 812; ^1^H NMR (600 MHz, DMSO-d_6_) δ 10.64 (1H, s), 7.18 (1H, t, *J* = 7.2 Hz), 7.12 (1H, d, *J* = 7.2 Hz), 6.88 (1H, d, *J* = 7.8 Hz), 6.67 (1H, t, *J* = 2.4 Hz), 5.81 (1H, t, *J* = 2.4 Hz), 5.49 (1H, br s), 4.91 (1H, s), 3.59 (3H, s); ^13^C NMR (150 MHz, DMSO-d_6_) δ 175.3, 144.5, 130.1, 128.4, 127.6, 125.1, 124.9, 122.6, 118.9, 108.6, 106.5, 45.5, 33.7; HRMS m/z (M^+^) calculated for C_13_H_11_BrN_2_O: 290.0055. Found: 290.0058.

### 5-Bromo-3-(1-methyl-1H-pyrrol-2-yl)indolin-2-one (KS17)

Yield 82% (119 mg), as a white solid: MP 112–114 °C; IR (ATR) ν_max_/cm^−1^ 3180, 2905, 2845, 1698, 1608, 1475, 1330, 1169, 1080, and 801; ^1^H NMR (600 MHz, DMSO-d_6_) δ 10.63 (1H, s), 7.41 (1H, dd, *J* = 7.8, 2.4 Hz), 7.18 (1H, s), 6.85 (1H, d, *J* = 8.4 Hz), 6.71 (1H, t, *J* = 2.4 Hz), 5.90 (1H, t, *J* = 3.0 Hz), 5.62 (1H, br s), 5.03 (1H, s), 3.54 (3H, s); ^13^C NMR (150 MHz, DMSO-d_6_) δ 176.0, 142.0, 131.4, 130.8, 127.3, 126.4, 123.1, 113.2, 111.4, 107.5, 106.4, 44.5, 33.7; HRMS m/z (M+) calculated for C_13_H_11_BrN_2_O: 290.0055. Found: 290.0058.

### 3-(Thiophen-2-yl)indolin-2-one (KS18)

Yield 75% (81 mg), as a brownish solid: MP 140–142 °C; IR (ATR) ν_max_/cm^−1^ 3363, 2928, 1698, 1608, 1461, 1261, 1104, 840, 731, and 563; ^1H^NMR (600 MHz, CDCl_3_) δ 9.25 (1H, s), 7.20 (1H, d, *J* = 7.8 Hz), 7.18–7.16 (2H, m), 6.98–6.94 (2H, m), 6.92–6.90 (1H, m), 6.85 (1H, d, *J* = 7.8 Hz), 4.80 (1H, s); ^13^C NMR (150 MHz, CDCl_3_) δ 177.5, 141.4, 137.6, 128.7, 127.0, 126.2, 125.3, 125.2, 122.7, 122.6, 110.3, 47.6; HRMS m/z (M^+^) calculated for C_12_H_9_NOS: 215.0405. Found: 215.0406.

### 5-Chloro-3-(thiophen-2-yl)indolin-2-one (KS19)

Yield 78% (98 mg), as a yellowish solid: MP 155–157 °C; IR (ATR) ν_max_/cm^−1^ 3034, 2921, 2855, 1701, 1619, 1467, 1307, 1228, 1067, 791, 701, 662, and 556; ^1^H NMR (600 MHz, CDCl_3_) δ 9.32 (1H, s), 7.19 (1H, t, *J* = 3.6 Hz), 7.17 (1H, s), 7.15–7.14 (1H, m), 6.93–6.92 (2H, m), 6.77 (1H, d, *J* = 8.4 Hz), 4.80 (1H, s); ^13^C NMR (150 MHz, CDCl_3_) δ 177.2, 140.0, 136.6, 130.4, 128.8, 128.1, 127.1, 126.4, 125.6, 125.5, 111.3, 47.7; HRMS m/z (M+) calculated for C_12_H_8_ClNOS: 249.0015. Found: 249.0019.

### 5-Bromo-3-(thiophen-2-yl)indolin-2-one (KS20)

Yield 80% (117 mg), as a yellow solid: MP 161–163 °C; IR (ATR) ν_max_/cm^−1^ 3172, 3109, 2922, 2853, 1708, 1680, 1470, 1308, 1218, 1191, 806, 690, and 559; ^1^H NMR (600 MHz, CDCl_3_) δ 9.25 (1H, s), 7.38–7.37 (1H, m), 7.36 (1H, s), 7.26–7.25 (1H, m), 7.02–6.99 (2H, m), 6.97 (1H, d, *J* = 8.4 Hz), 4.87 (1H, s); ^13^C NMR (150 MHz, CDCl_3_) δ 177.0, 140.3, 136.6, 131.7, 130.8, 128.4, 127.1, 126.5, 125.6, 115.4, 111.7, 47.6; HRMS m/z (M^+^) calculated for C_12_H_8_BrNOS: 292.9510. Found: 292.9512.

## Antibacterial Activity Studies

### Bacterial strains

*S*. *aureus* (ATCC 25923), *S*. *epidermidis* (ATCC 12228), *S*. *saprophyticus* (ATCC 15305), *E*. *coli* (ATCC 25922), *P*. *aeruginosa* (ATCC 27853), and Methicillin-resistant *S*. *aureus* (MRSA) strains were isolated from Difco Tryptic Soy Agar (TSA; BD, USA) plates and inoculated in BBL Muller-Hinton Broth (MHB; BD, USA) for the preparation of the inoculum used in the antibacterial activity assays.

### Screening of active compounds with anti-*S*. *aureus* activity

The screening for compounds expressing anti-*S*. *aureus* activity was performed by using a disc diffusion method^40^. For all bacterial strains, overnight culture grown in MHB (400 μL) was inoculated into 20 mL of Difco Muller Hinton Agar (MHA; BD, USA) and solidified on petri dishes with a diameter of 90 mm (SPL, Korea). Sterile filter discs (6 mm in diameter; Advantec MFS, Taiwan) were placed on the surface of inoculated agar plates. Compounds, to a final amount of 20 μg, were impregnated by dropping 10 μL of 2 mg/mL stock solution to the sterile filter discs. The plates were then incubated at 37 °C for 24 h, and antibacterial activity was evaluated through the measurement of the diameter of the inhibition zone (mm) by using a transparent ruler. Each compound was analysed a minimum of three times and one representative image was presented.

### Validation of MRSA strains

MRSA strains were purchased from CCARM (Culture Collection of Antimicrobial Resistant Microbes; www.ccarm.or.kr). The verification of strains was performed in two independent experiments. First, assay plates for both six individual MRSA strains and *S*. *aureus* (ATCC 25923) were prepared as described in disc diffusion method and the susceptibility to oxacillin was determined by using an E-test Oxacillin (BioMérieux, France). Second, *S*. *aureus* strains were subjected to PCR amplification for the detection of *mec*A gene in accordance with a previous report^41^. The PCR protocol was performed in a total 20 μL volume using 2x Quick Taq mix (Toyobo, Japan). The PCR program was conducted with initial denaturation at 94 °C for 3 min, followed by 28 cycles of 94 °C for 30 s, 55 °C for 30 s, and 68 °C for 30 s, and ended with a final extension at 68 °C for 5 min. The PCR product was electrophoresed on 2% agarose gel and imaged with an E-Graph Gel Documentation System (Atto, Japan). Synthesized primers mecA-F (5′-GTAGAAATGACTGAACGTCCGATGA-3′) and mecA-R (5′-CCAATTCCACATTGTTTCGGTCTAA-3′) were used in the amplification, which produced a PCR amplicon of 310 bp in size.

### Determination of MIC

Microbroth dilution MIC testings were performed^42^. Briefly, fresh overnight cultured cells were grown until absorbance at 600 nm (A_600_) value of 0.5 was reached and the 500-fold diluted cells were inoculated into 200 μL of MHB in 96-well plates (SPL, Korea) and cultured cells for 16 h at 37 °C with shaking at 250 rpm by using an Incu-Mixer MP (Benchmark Scientific, USA). The MIC value is defined as the minimal concentration of chemical or antibiotic that reduced the turbidity of non-treated bacterial inoculum to 90% after overnight incubation and determined as follows: A_600_ of individual cells grown in 96-well plates was measured using a SPECTROstar Nano (BMG LABTECH, Germany) and MIC values were calculated from the plots of A_600_ vs. the concentrations of compounds. The representative MIC values of at least five independent experiments were shown. All statistical analyses were performed by using SigmaStat (Ver. 4.0) (Systat Software, Inc., USA). P-values of <0.05 were considered to indicate statistically significant difference.

### Screening for antibiotics that exhibit synergistic effects with KS16

To evaluate the combinatorial activity of **KS16** with available antibiotics, 5 μL of DMSO or **KS16** (final concentration, 2.5 μg/mL) was added to 195 μL of Muller Hinton broth inoculated with ATCC 25923 cells in a Sensititre Gram-positive MIC plate (EUST, Thermo Scientific, USA) and determined MIC by the microbroth dilution method, as described above, with the addition of AlamarBlue cell viability reagent (Thermo Scientific, USA) to allow the detection of bacterial growth in accordance with the manufacturer’s instructions. Therefore, the final concentrations of the working MIC plates were diluted to one-fourth of that in the original EUST plates.

### Determination of synergistic action of compounds to antibiotics

The fractional inhibitory concentration indices (FICIs) of the designed combinations against *S*. *aureus* were determined by using checkerboard assays, as previously described^43^ under the same conditions used to screening the antibiotics to evaluate the combinatorial activity between antibiotics and selected compounds.

### Characterization of proteins affected by KS16

Total cellular proteins extracted from ATCC25923 cells (1 × 10^9^) treated with different concentrations of **KS16** in 96-well plates were resolved in 12% TGX strain-free gels (Bio-Rad), followed by imaging with ChemiDoc-MP (Bio-Rad). Amounts of protein bands migrated on the gel was analyzed with Image Lab Software (v. 5.2.1; Bio-Rad, USA). For peptide mass fingerprinting (PMF), gels were stained with Brilliant Blue R (Sigma-Aldrich, USA) and interesting protein bands were excised from the gel, followed by subjecting them to in-gel trypsin digestion as described^44^. Then, the in-gel digested sample was subjected to MALDI-TOF MS (Microflex LRF 20, Bruker, USA) as previously described^45^. Spectra were acquired from 300 shots per spectrum in the m/z range of 600–3,000 and calibrated by two-point internal calibration using trypsin auto-digestion peaks (m/z = 842.5099 and 2211.1046). The processing and peak picking of the spectra were automatically carried out using Flex analysis software v3.0. Threshold used for peak-picking was as follows: 500 for minimum resolution of monoisotopic mass and 5 for S/N. The MS/MS database search was conducted in NCBInr and Swiss-databases using the online MS/MS ion search software, Mascot (http://www.matrixscience.com/) with subsequent parameters: trypsin as the cleaving enzyme, a maximum of one missed cleavage, iodoacetamide (Cys) as a complete modification, oxidation (Met) as a partial modification, monoisotopic masses, and a mass tolerance of ±0.1 Da. PMF acceptance criteria is probability scoring.

### Cytotoxicity assay

The cytotoxicity assay was performed as described in a previous report^46^ against human primary skin fibroblast cells (ATCC CRL 2097, USA) with approximately 5 × 10^3^ cells/well in 96-well tissue culture plates (Thermo Scientific, USA). The cells with media containing **KS16** in indicated concentrations were incubated at 37 °C for 24 and 48 h, respectively. Afterwards, one tenth of a total volume of CCK-8 solution was added to each well and the plate was incubated at 37 °C for 3 h. A_450_ was measured by SpectraMax M3 Multi-Mode Microplate Reader (Molecular devices, USA). Percentage viability of **KS16**-treated human fibroblasts to control was presented. All statistical analyses were performed by using SigmaStat (Ver. 4.0) (Systat Software, Inc., USA).

## Results and Discussion

### Chemistry

The 3-indolylindolin-2-ones **KS1**-**KS12** were synthesized by novel indium (III)-catalysed reaction of the corresponding 3-diazoindolin-2-ones **1** with indoles **2** as one-pot procedure (Fig. 2). Treatment of **1a** with **2a** using 10 mol% of In(OTf)_3_ as a catalyst in 1,2-dichloroethane at 50 °C for 6 h produced **KS1** in 80% yield. The substrates bearing electron-withdrawing and -donating groups were well-tolerated and the desired products were obtained. For example, the reactions of **1b** or **1c** bearing electron-donating groups 5-Me and 5-OMe with **2a** provided **KS2** and **KS3** in 72 and 74% yield, respectively, whereas reactions of diazo compounds **1d**–1H bearing electron-withdrawing groups, 5-F, 5-Cl, 7-Cl, 4-Br, and 5-Br, with **2a** afforded the desired products **KS4**-**KS8** in 60–77% yield. Inspired by these results, we investigated the possibility of utilizing substituted indoles in our synthetic approach. Treatment of **1a** with 5-methoxy-1*H*-indole (**2b**) bearing an electron-donating group in 1,2-DCE at 50 °C for 6 h afforded product **KS9** in 76% yield and combination with 5-fluoro-1*H*-indole (**2c**) bearing an electron-withdrawing group provided **KS10** in 62% yield. Additional reactions of **1c** with **2b** or **2c** led to the desired products **KS11** and **KS12** in 75 and 70% yield, respectively.

Encouraged by these results, further indolin-2-ones **KS13**-**KS20** bearing 1-methylpyrrol-2-yl and thiophen-2-yl group at the 3-position were synthesized starting from 3-diazoindolin-2-ones **1** and 1-methylpyrrole (**3**) or thiophene (**4**) (Fig. 3). Treatment of **1a** with **3** in the presence of 10 mol% In(OTf)_3_ in 1,2-DCE at room temperature for 4 h afforded the expected product **KS13** in 71% yield. Additional reaction of **1b** bearing an electron-donating group with **3** produced **KS14** in 91% yield and combination of **1g**-**1i** with **3** afforded the corresponding products **KS15**-**KS17** in 64–82% yield. Meanwhile, further coupling reactions of **1a**, **1e**, or 1H with thiophene (**4**) afforded desired products **KS18**-**KS20** in 75–80% yield. All synthetic compounds **KS1** to **KS20** were characterized by the analysis of their spectral data.

### Screening of oxindole derivatives as anti-*S*. *aureus* agents

**Figure 4:**
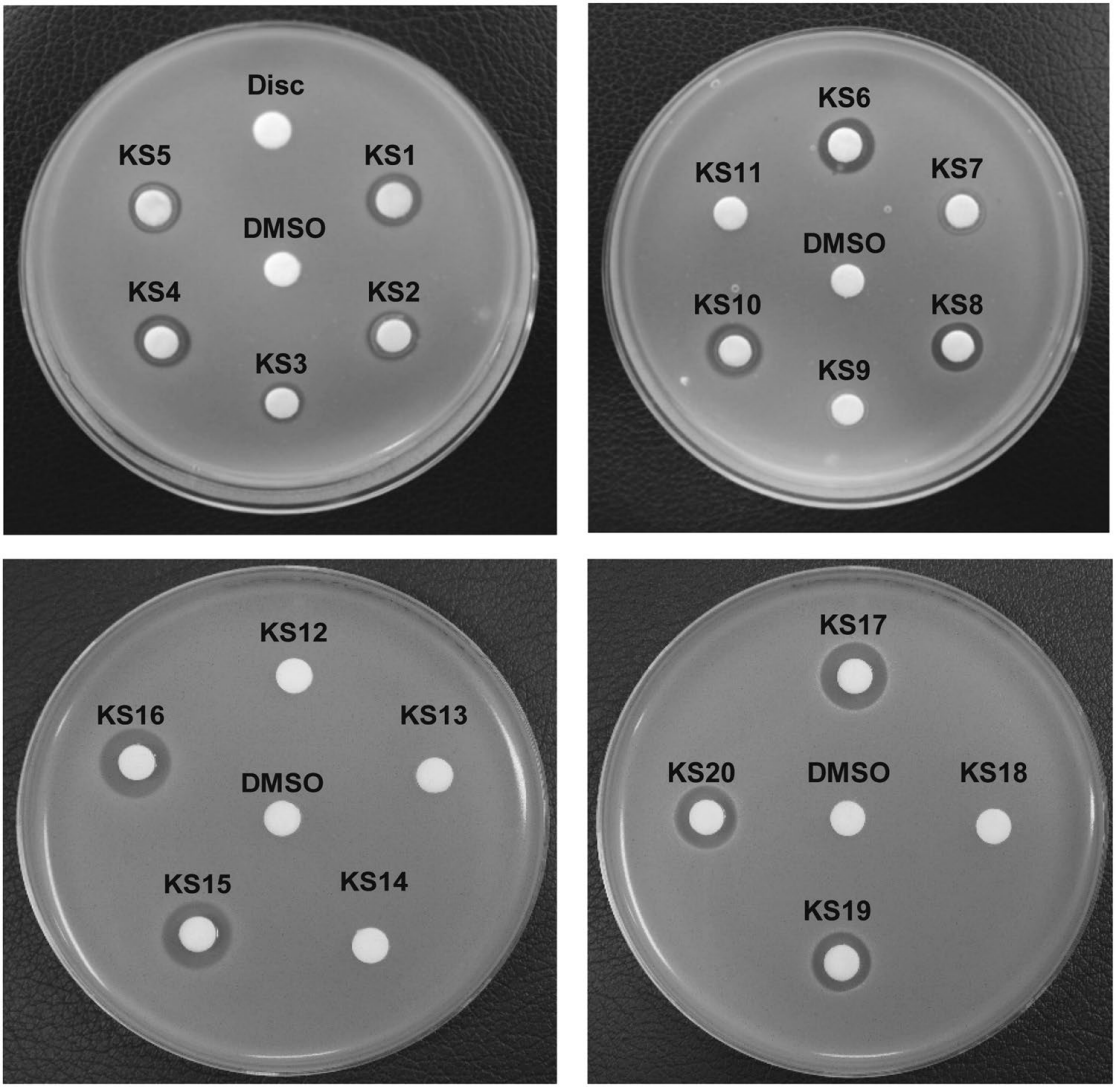
Screening of anti-*S*. *aureus* activity of 20 compounds. Newly synthesized compounds (20 μg) were tested for their individual activity against *S*. *aureus* (ATCC 25923) by using a disc fusion assay. Representative images from n = 3 are shown. The zone of inhibition (mm) was measured and is listed in Table 1. DMSO and Disc were used as negative controls. Agar plates were photographed and one of the representative images from n = 5 were processed in Adobe Illustrator CS6 (v16.0.0, Adobe Systems Inc., USA).

**Figure 5:**
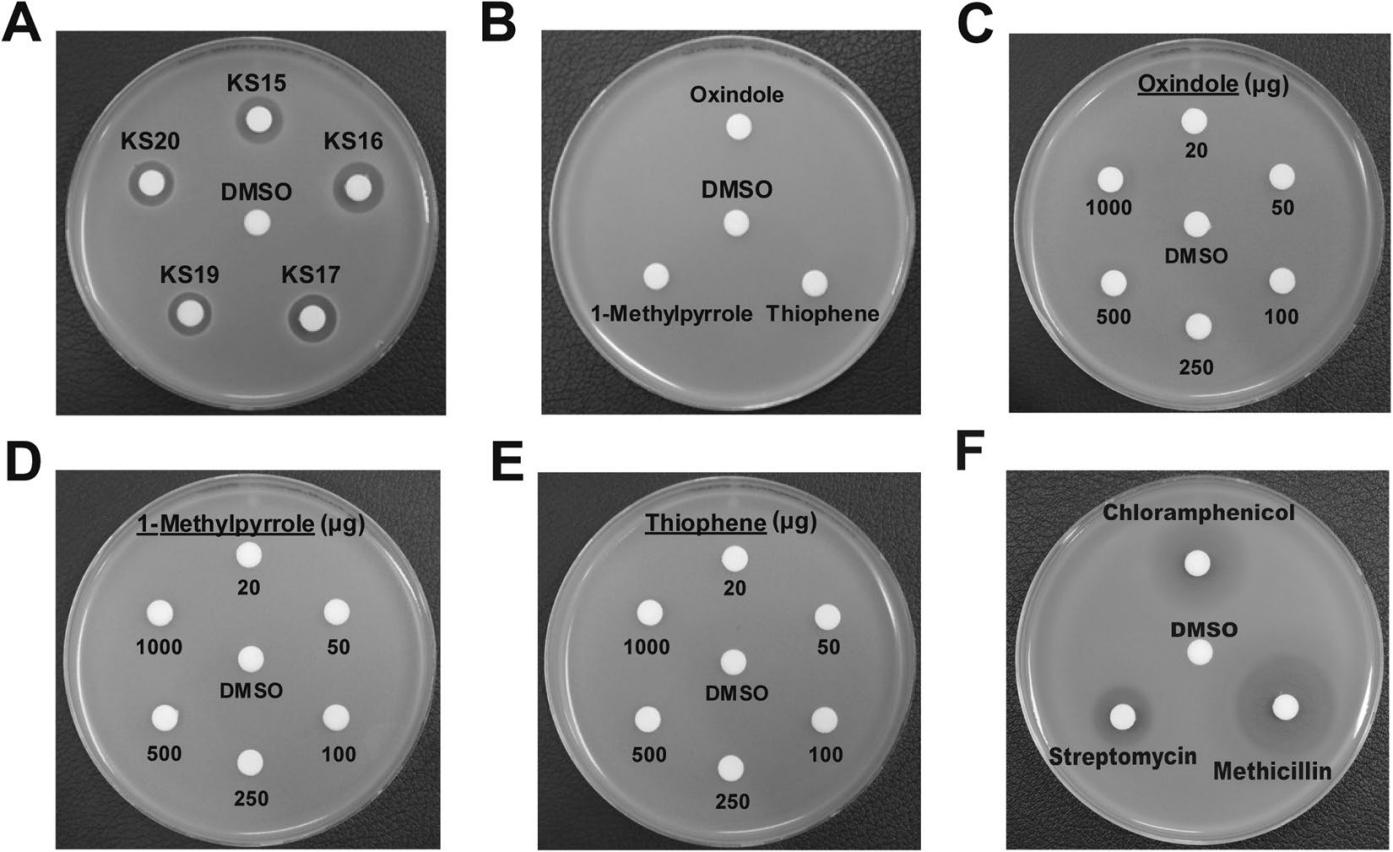
Comparison of effective chemicals, their synthetic moieties, and commercial antibiotics in expression of anti-*Staphylococcus* activity. Activity of (**A**) five highly active compounds (**KS15**, **KS16**, **KS17**, **KS19**, and **KS20**), (**B**–**E**) parental moieties used for synthesis of above compounds and (**F**) commercial antibiotics (chloramphenicol, methicillin, and streptomycin) against *S*. *aureus*. 20 μg of synthesized chemicals (**A**,**B**,**F**) were used to measure the activity, whereas 20 to 1,000 μg were used to evaluate the activity of the substrates. (**C**–**E**) Agar plates were photographed and one of the representative images from n = 5 were processed in Adobe Illustrator CS6 (v16.0.0, Adobe Systems Inc., USA).

The newly synthesized heterocyclic indolin-2-ones were next evaluated for their antibacterial activity against *S*. *aureus* by using the disc diffusion method. The anti-*S*. *aureus* activity for the 20 individual compounds (**KS1** to **KS20**) evaluated showed that 14 chemicals produced a clear zone of inhibition against *S*. *aureus* (Figs 4, 5 and Table 1). Among them, five compounds (**KS15**, **KS16**, **KS17**, **KS19**, and **KS20**) that produced a clear zone of >10 mm were selected as the target compounds (Figs 4, 5A and Table 1). To demonstrate the enhanced antibacterial activity of the synthesized compounds, we have evaluated the discrete antibacterial activity of the starting materials (1-methylpyrrole, oxindole, or thiophene) used for the synthesis of the active compounds under equal amounts (20 μg). However, none of these substrates resulted in any clear zone of inhibition against *S*. *aureus* at 20 μg (Fig. 5B and Table 1). To compare the effectiveness of the target compounds to that of the nuclear compounds, different amounts (between 20 and 1,000 μg) of 1-methylpyrrole, oxindole, and thiophene were evaluated for the production of a clear zone. We found that only oxindole at 1,000 μg produced a turbid zone (Fig. 5C), whereas other compounds did not exhibit such inhibitory activity (Fig. 5D,E). These results indicated that the newly synthesized chemical compounds were at least > 50-fold more active than the starting materials. Moreover, the compounds showed the comparable activity to streptomycin, while slightly less active to chloramphenicol, erythromycin, and methicillin (Table 1 & Fig. 5F). From a structural perspective (Fig. 3), five active compounds (**KS15**, **KS16**, **KS17**, **KS19**, and **KS20**) have common structural features: C-4/C-5-halogenated indolin-2-ones bearing 5-membered heterocycles, 1-methylpyrrol-2-yl or thiophen-2-yl group, at the 3-position. Meanwhile, 3-indolylindolin-2-ones **KS1**-**KS12** were relatively inactive, which indicated that the nature of the functionalization at 3-position was a key component in the regulation of function. Specifically, active **KS15**-**KS17** were either brominated or chlorinated at the 4- or 5-position on the benzene ring of the indolin-2-one moiety, whereas inactive **KS13**-**KS14** were either non-halogenated at 4- or 5-position or methoxy-substituted at 5-position. Hence, halogenation at the 5-position of the indolin-2-one scaffold appears to be an important factor of the activity. This structural effect on the enhanced antibacterial activity was further observed for compounds **KS19** and **KS20** bearing -Br or -Cl group at the 5-position.

**Table 1:** Evaluation of anti-*S*. *aureus* activity of target compounds, their parental moieties, and commercial antibiotics^*a*^.

Compound	Size of zone, mm
DMSO	ND
Chloramphenicol	20.0 ± 0.7
Methicillin	24.2 ± 0.8
Streptomycin	15.8 ± 0.8
Indole	ND
Oxindole	ND
Thiophene	ND
1-Methylpyrrole	ND
**KS1**	9.1 ± 0.2
**KS2**	9.2 ± 0.3
**KS3**	8.0 ± 0.4
**KS4**	9.2 ± 0.7
**KS5**	9.4 ± 0.5
**KS6**	8.9 ± 0.5
**KS7**	8.4 ± 0.2
**KS8**	9.1 ± 0.2
**KS9**	ND
**KS10**	9.5 ± 0.3
**KS11**	ND
**KS12**	ND
**KS13**	ND
**KS14**	ND
**KS15**	12.5 ± 0.8
**KS16**	12.5 ± 0.9
**KS17**	12.1 ± 0.8
**KS18**	ND
**KS19**	11.2 ± 0.4
**KS20**	11.4 ± 0.7

^a^Zone of inhibition (Figs 4 and 5) for individual compounds against S. aureus (ATCC 25923) is listed. The values shown are averaged from n = 3. Equal amounts of each compound (20 μg) resuspended in DMSO were used in the assays. ND indicates that clear zone was not observed.

In general, the SAR results indicated that heterocyclic indolin-2-ones **KS13**-**KS20** bearing 1-methylpyrrol-2-yl or thiophen-2-yl group at the 3-position on the indolin-2-one moiety and the halogenation at the 4- or 5-position on the benzene ring of the oxindole moiety significantly influenced their anti-*S*. *aureus* activities.

### Determination of MIC of compounds against *S*. *aureus*

**Figure 6:**
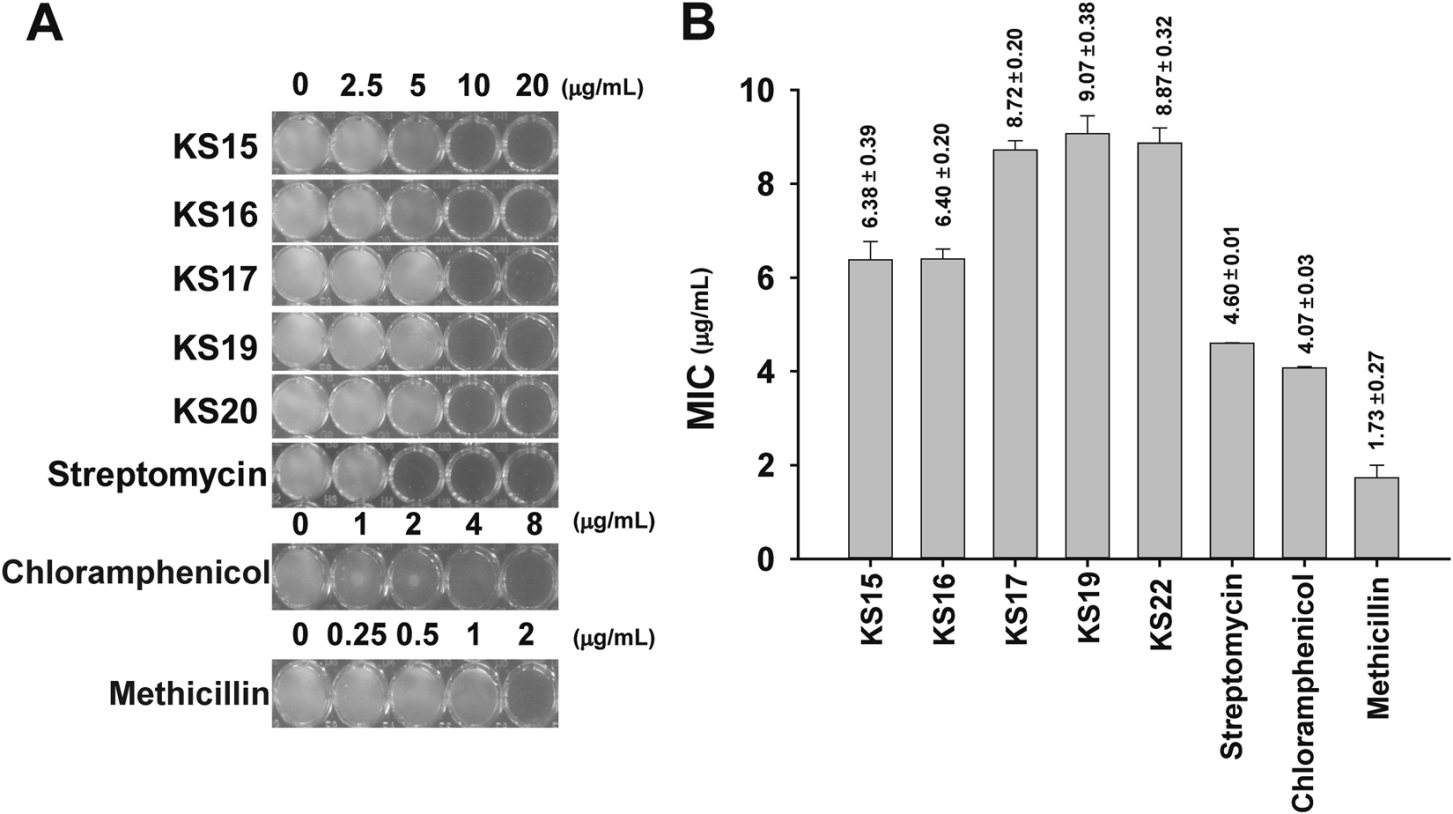
Determination of MIC against *S*. *aureus*. Antibacterial activity of the newly synthesized compounds and commercial antibiotics (chloramphenicol, methicillin, and streptomycin) against *S*. *aureus* was determined. (**A**) Effect of cell growth of ATCC25923 strain by different compounds. The cells were grown in 96-well plates with different concentrations of **KS15**, **KS16**, **KS17**, **KS19**, **KS20**, chloramphenicol, methicillin, and streptomycin. Images of 96-wells for individual compounds with indicated concentrations were photographed. One of the representative images from n = 5 were processed in Adobe Illustrator CS6 (v16.0.0, Adobe Systems Inc., USA). (**B**) The mean MIC values of the individual compounds. The data was processed from n = 5.

We further evaluated the anti-*S*. *aureus* activity of the target compounds by determining the MIC for different concentrations of **KS15**, **KS16**, **KS17**, **KS19**, and **KS20** in comparison to chloramphenicol, methicillin, and streptomycin as control antibiotics. We found that the MIC values for **KS15**, **KS16**, **KS17**, **KS19**, and **KS20** were 6.38, 6.40, 8.72, 9.07, and 8.87 μg/mL, respectively (Fig. 6A,B), whereas the MIC value for chloramphenicol, methicillin, and streptomycin, was 4.60, 4.07, and 1.73 μg/mL, respectively. Owing to the lower MIC value of **KS15** and **KS16** compared with **KS17**, the position of the halogen at the 4-position of oxindoles with 1-methylpyrrole or thiophene at 3-position appeared to be the most important moieties for enhancing anti-*S*. *aureus* activity. Moreover, our newly synthesized compounds were comparable with commercial antibiotics, which suggested that our compounds are potentially good candidate for antibiotics.

### Specificity of compounds against *Staphylococcus* species

**Figure 7:**
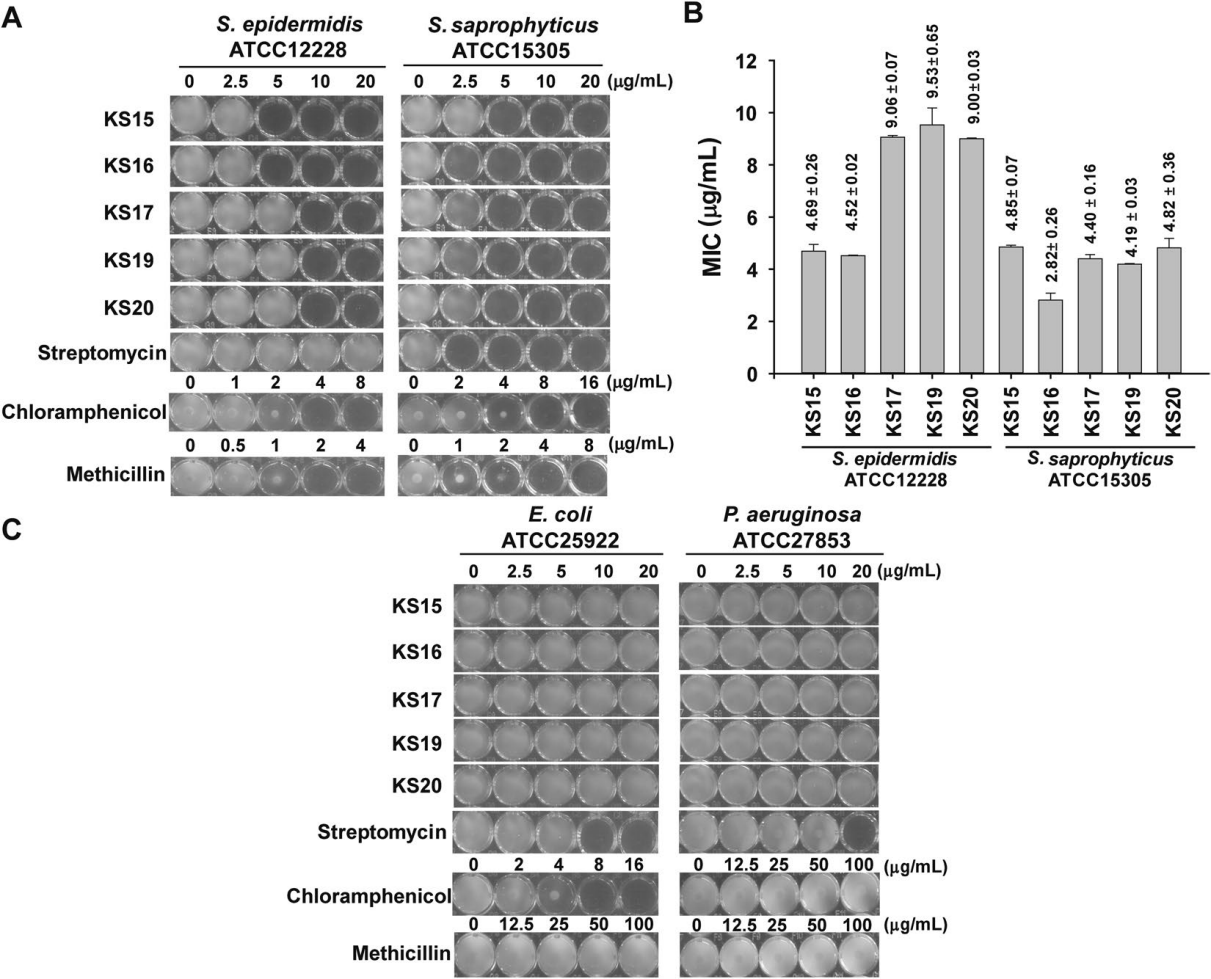
Analysis of the specificity of active compounds on Gram-positive and -negative strains. (**A**) Antibacterial activity of **KS15**, **KS16**, **KS17**, **KS19**, **KS20**, and chloramphenicol, methicillin, and streptomycin. The activity was determined with different concentrations of compounds against Gram-positive *S*. *epidermidis* (ATCC 12228) (left) and *S*. *saprophyticus* (ATCC 15305) (right). (**B**) Determination of MIC of compounds. Values indicated were averaged from n = 5. (**C**) Evaluation of antibacterial activity against Gram-negative species. The growth of *E*. *coli* (ATCC 25922) and *P*. *aeruginosa* (ATCC 27853) with different concentrations of **KS15**, **KS16**, **KS17**, **KS19**, **KS20**, chloramphenicol, methicillin, and streptomycin was depicted. Images of 96-wells for individual compounds with indicated concentrations were photographed. In (**A**,**B**) one of the representative images from n = 5 were processed in Adobe Illustrator CS6 (v16.0.0, Adobe Systems Inc., USA).

We speculated whether the active compounds selectively inhibited the growth of *S*. *aureus*. To answer this question, we determined the MIC of the compounds against subspecies of *Staphylococcus* such as *S*. *epidermidis* (ATCC 12228) and *S*. *saprophyticus* (ATCC 15305) and compared them with that of chloramphenicol, erythromycin, methicillin, and streptomycin. From the data, we found that all five compounds (**KS15**, **KS16**, **KS17**, **KS19**, and **KS20**) were active against tested species (Fig. 7A,B) with equivalent or higher efficacy with *S*. *epidermidis* and *S*. *saprophyticus*, respectively, than *S*. *aureus* (Figs 6 and 7A,B). Meanwhile, commercial antibiotics showed different activity among species. For example, chloramphenicol and methicillin, were found to be active against both *S*. *saprophyticus* and *S*. *epidermidis*, while streptomycin was inactive against *S*. *epidermidis* (MIC > 20 μg/mL) (Fig. 7A). Therefore, the experimental results suggest that the heterocyclic oxindole derivatives express a broader and specific range of antibacterial activity against members of the common colonization species of *S*. *aureus* than the control antibiotic, streptomycin.

We further tested whether new compounds are effective against two Gram-negative bacteria. First, *E*. *coli* strain (ATCC 25922) was used to examine the effect of compounds on the inhibition of bacterial growth. As shown in Fig. 7C (left), all compounds were inactive (MIC > 20 μg/mL) as methicillin (MIC > 100 μg/mL) against *E*. *coli* cells, while chloramphenicol and streptomycin showed inhibitory effects. Second, we measured MIC values against *P*. *aeruginosa*, a common interactor with *S*. *aureus* in chronic wounds and other types of infections^47–53^. As shown in Fig. 7C (right), the MIC values of all compounds against *P*. *aeruginosa* (ATCC 27853) were >20 μg/mL, as seen for *E*. *coli*, whereas streptomycin showed inhibitory effects only at 20 μg/mL, but this concentration was regarded as non-effective considering that <10 μg/mL of compounds was effective against *Staphylococcus* species (Figs 6B and 7B). Moreover, chloramphenicol and methicillin showed almost no activity to *P*. *aeruginosa* (MIC > 100 μg/mL; Fig. 7C, right). All the above data showed that compounds (**KS15**, **KS16**, **KS17**, **KS19**, and **KS20**) are specific to *Staphylococcus* species and indicated that such compounds could be utilized for the eradication of *Staphylococcus* species as narrow spectrum antibiotics in infections co-colonized with *P*. *aeruginosa* or other Gram-negative bacteria.

### Activity of compounds against MRSA strains

**Figure 8:**
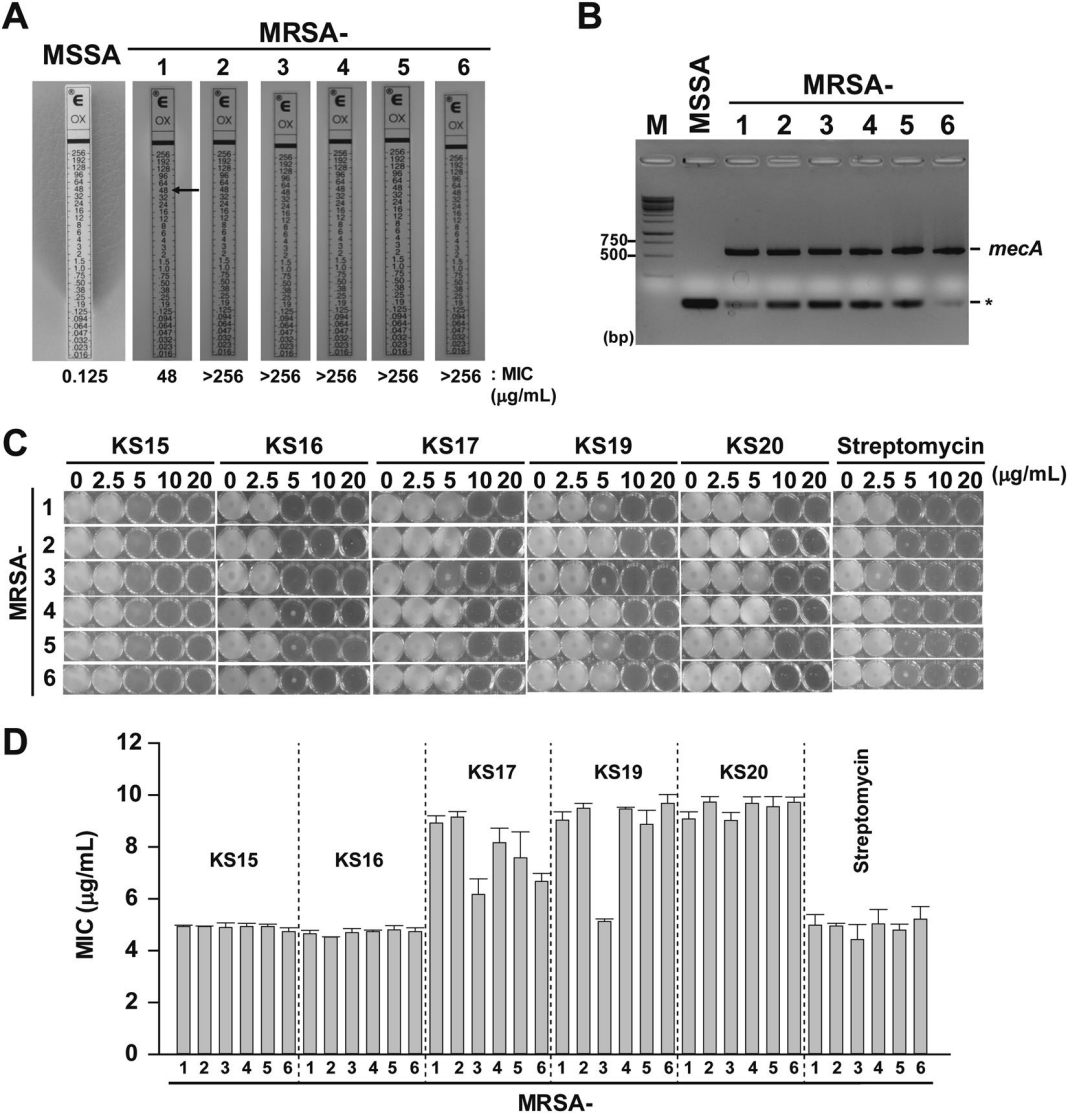
Activity of compounds against MRSA strains. (**A**) Confirmation of MRSA strains. MIC values for MSSA (ATCC 25923) and six MRSA strains were analyzed by the concentration diffusion assay using an E-test on MHA plate. Arrow indicates the MIC of oxacillin for individual strains. The data shown are representatives of n = 3. (**B**) PCR confirmation of the *mecA* gene. PCR amplification using *mecA*-specific primer sets was performed for MSSA (ATCC 25923, Lane 1) and 6 MRSA strains (Lanes 2–7). M indicates DNA size marker (GeneRuler 100 bp Plus DNA ladder; Thermo Scientific). Asterisk (*) indicates non-specific PCR product. (**C**) Growth analysis of MRSA strains against compounds and streptomycin. Different concentrations of compounds were added to ATCC 25923 cells and cultured for 24 h. One representative experimental data from n = 5 was shown. (**D**) Determination of MIC. The values (μg/mL) were averaged from n = 5. All photographed images shown in (**A**) to (**C**) were processed in Adobe Illustrator CS6 (v16.0.0, Adobe Systems Inc., USA).

We expected that the active compounds would be also effective for the inhibition of clinically-isolated MRSA strains. MRSA strains to be tested were confirmed by PCR for the amplification of the *mecA* gene^41^, a defining standard in the determination of the resistance of *S*. *aureus* to methicillin including MIC determination. The data showed that all six MRSA strains tested were much less sensitive than MSSA against oxacillin (Fig. 8A) and produced a *mecA* PCR product of 533 bp in size (Fig. 8B). Furthermore, we have determined the MIC for five active compounds (**KS15**, **KS16**, **KS17**, **KS19**, and **KS20**) against MRSA strains. The results showed that MIC values for the compounds against all MRSA strains were between 4.5 and 9.7 μg/mL (Fig. 8C,D), which were similar or slightly lower than that of ATCC 25923 (MSSA). Interestingly, **KS15** and **KS16** showed stronger activity against MRSA than against MSSA, which suggested that **KS15** and **KS16** might be potential compounds for the eradication of MRSA strains which may have been due to the unique functionalization in comparison to other evaluated compounds. The results can provide further insights on how to alter the antibacterial activity of the heterocyclic-bearing oxindole derivatives with specific functionalization at different positions. Therefore, our compounds may be alternative antibiotics for the specific eradication of both methicillin-susceptible and methicillin-resistant *Staphylococcus* species.

### Synergistic action of KS16 with aminoglycosides antibiotics

**Figure 9:**
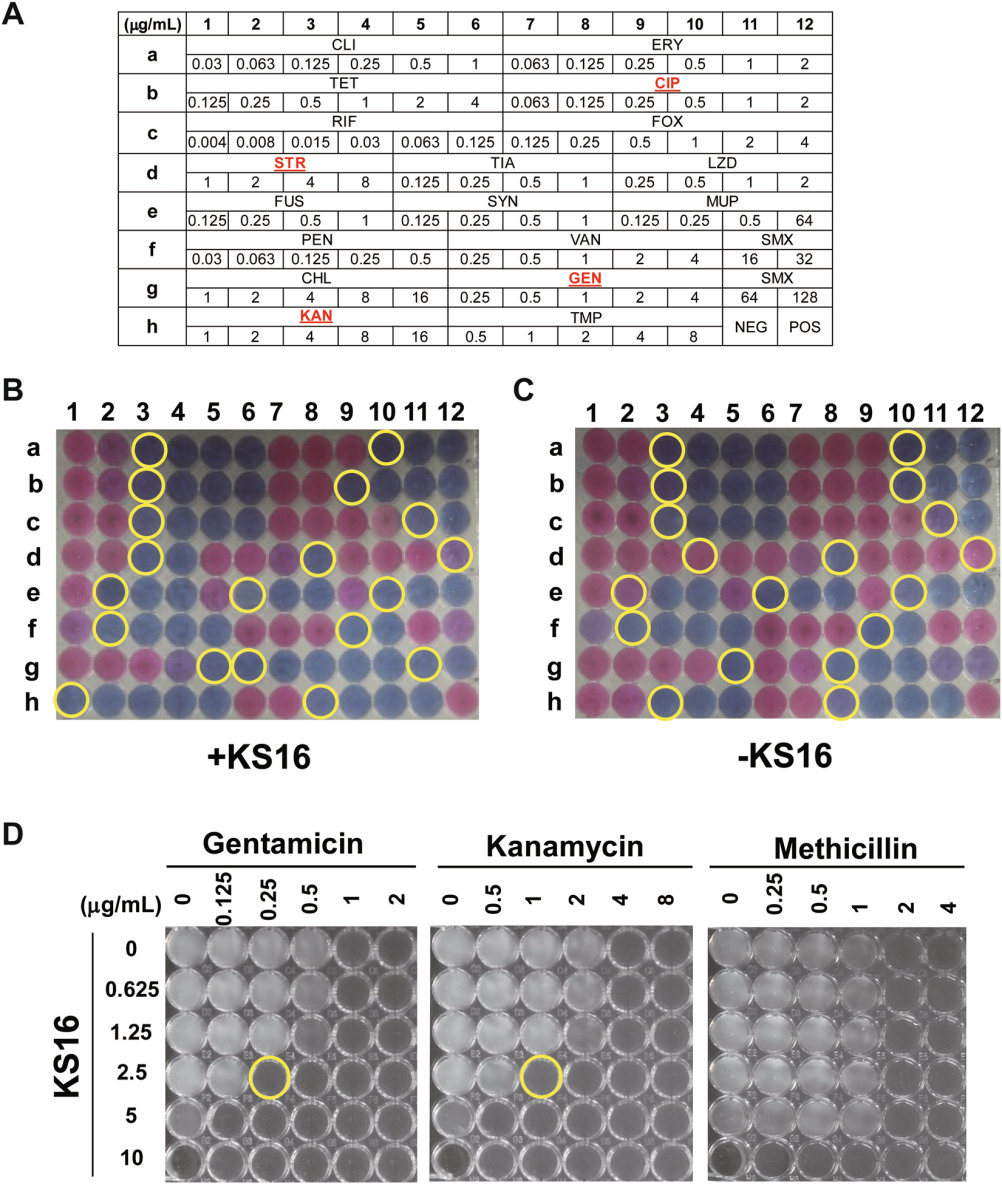
Synergistic activity of **KS16** to antibiotics. (**A**) Information on antibiotics and the working concentration. Sensititre Gram-positive MIC plate (Cat. No. EUST) with final concentration of individual antibiotics shown. CHL, Chloramphenicol; CIP, Ciprofloxacin; CLI, Clindamycin; ERY, Erythromycin; FOX, Cefoxitin; FUS, Fusidate; GEN, Gentamicin; KAN, Kanamycin; LZD, Linezolid; MUP, Mupirocin; PEN, Penicillin; RIF, Rifampin; SMX, Sulfamethoxazole; STR, Streptomycin; SYN, Quinupristin/Dalfopristin; TET, Tetracycline; TIA, Tiamulin; TMP, Trimethoprim; VAN, Vancomycin; NEG and POS indicate the negative and positive control, respectively. The antibiotics induced by **KS16** are indicated in yellow. (**B**,**C**) Effect of **KS16** on the susceptibility to antibiotics. ATCC 25923 cells were cultured in a Sensititre Gram positive MIC plate (Cat. No. EUST) with (**B**) or without (**C**) **KS16** (2.5 μg/mL). Yellow circle of the plate was indicated MIC for each antibiotic. (**D**) Checkerboard assays for gentamicin, kanamycin, and methicillin. Different concentrations of **KS16** and antibiotics were used to determine the susceptibility of ATCC 25923 cells. The yellow circles indicate the concentrations showing FICI = 0.5. One of representative from n = 3 was shown. All images of (**B**–**D**) were photographed and processed in Adobe Illustrator CS6 (v16.0.0, Adobe Systems Inc., USA).

The five compounds (**KS15**, **KS16**, **KS17**, **KS19**, and **KS20**) showed comparable activity to *S*. *aureus* species with a commercial antibiotic, streptomycin (Fig. 4 & Table 1). Moreover, above compounds showed specific activity to *Staphylococcus* species unlike streptomycin (Figs 6 and 7). This indicates that newly synthesized compounds could directly use as specific anti-*S*. *aureus* agents. To understand their plausible mechanisms, we approached to identify synergistic antibiotics that is increased their activities by new active compounds assuming that the antibiotics and compounds inhibit the same pathway for the killing of *S*. *aureus*. To this end, we selected **KS16**, one of the most active compounds in this study, and evaluated its combinatorial action with existing antibiotics planted in a pre-made Sensititre Gram-positive MIC plate (Fig. 9A) by determining MIC values of with or without 2.5 μg/mL of **KS16**, the concentration at which no killing of *S*. *aureus* was found (Fig. 6). Results showed that the addition of **KS16** to the antibiotics (ciprofloxacin, gentamicin, kanamycin, and streptomycin) increased the susceptibility to *S*. *aureus* compared with the control sample (Fig. 9B,C; Table 2). Addition to antibiotics seeded on the pre-made Sensititre Plate, we separately evaluated the effect of **KS16** to the susceptibility to methicillin, an ineffective antibiotic to MRSAs. Neither the increase in MIC values of methicillin itself (2.0 μg/mL) nor the synergistic action by **KS16** was detected (Fig. 9D). Therefore, **KS16** may not expand the arsenal of therapeutic agents for eradicating MRSAs. Interestingly, all selected antibiotics, except ciprofloxacin, a fluoroquinolone class broad-spectrum antibiotic, are known to work as protein synthesis inhibitors targeting the bacterial ribosome. Among them, the gentamicin and kanamycin susceptibility to *S*. *aureus* was increased to more than 4-fold. To evaluate the effect of potency of the combination of **KS16** and gentamicin or kanamycin in comparison to their individual activities, synergy testing by checkerboard assay was performed and found that combinations of **KS16** with gentamicin or kanamycin expressed FICI of 0.5 (Fig. 9D), an expected value as synergistic drug combination.

**Table 2:** Synergistic activity of **KS16** on antibiotics.

Antibiotics	Acronym	Subclass (cellular target)	MIC (μg/mL)	
			−KS16	+KS16
Cefoxitin	FOX	β-lactam (penicillin-binding proteins)	2	2
Chloramphenicol	CHL	Amphenicol (50 S)	16	16
Ciprofloxacin	CIP	Fluoroquinolone (DNA gyrase)	0.5	0.25
Clindamycin	CLI	Lincosamide (50 S)	0.125	0.125
Erythromycin	ERY	Macrolide (50 S)	0.5	0.5
Fusidate	FUS	Steroid (EF-G)	0.25	0.25
Gentamicin	GEN	Aminoglycoside (30 S)	1	0.25
Kanamycin	KAN	Aminoglycoside (30 S)	4	1
Linezolid	LZD	Oxazolidinone (50 S)	>2	>2
Methicillin	MET	β-lactam	2.0	2.0
Mupirocin	MUP	Isoleucyl t-RNA synthetase	0.25	0.25
Penicillin	PEN	β-lactam (peptidoglycan)	0.063	0.063
Quinupristin/Dalfopristin	SYN	Lincosamide (50 S)	0.25	0.25
Rifampin	RIF	Rifampicin (RNA polymerase)	0.015	0.015
Streptomycin	STR	Aminoglycoside (30 S)	8	4
Sulfamethoxazole	SMX	Sulphonamide (Dihydropteroate synthase, DHPS)	64	64
Tetracycline	TET	Tetracycline (30 S)	0.5	0.5
Tiamulin	TIA	Pleuromutilin (50 S)	1	1
Trimethoprim	TMP	Dihydrofolate reductase	2	2
Vancomycin	VAN	Glycopeptide (cell wall)	2	2

Sensititre Gram-positive MIC plate (Cat. No. EUST) except methicillin was used to determine the MIC value (μg/mL). Five independent experiments were performed and reproducible MIC values are listed in the Table. Antibiotics that induced activity through **KS16** are indicated in red. The MIC values decreased by **KS16** are indicated in red.

### Increased cellular level of autolysin by KS16

Checkerboard assays indicate that **KS16** is a compound for the synergistic combination with gentamicin and kanamycin by sharing the part of cellular targets or pathways for the antibacterial action by gentamicin and kanamycin. Second possibility is that **KS16** increases the permeability for the entry into ribosome targeting antibiotics as reported on the combination of gentamicin and vancomycin although the exact mechanism of synergistic combination has not been verified^54^. In opposite to broad-spectrum activity of gentamicin and kanamycin to multiple species^55^, **KS16** may target gene product(s) only presented within *S*. *aureus* to function as both antibacterial and synergistic agent with high specificity.

**Figure 10:**
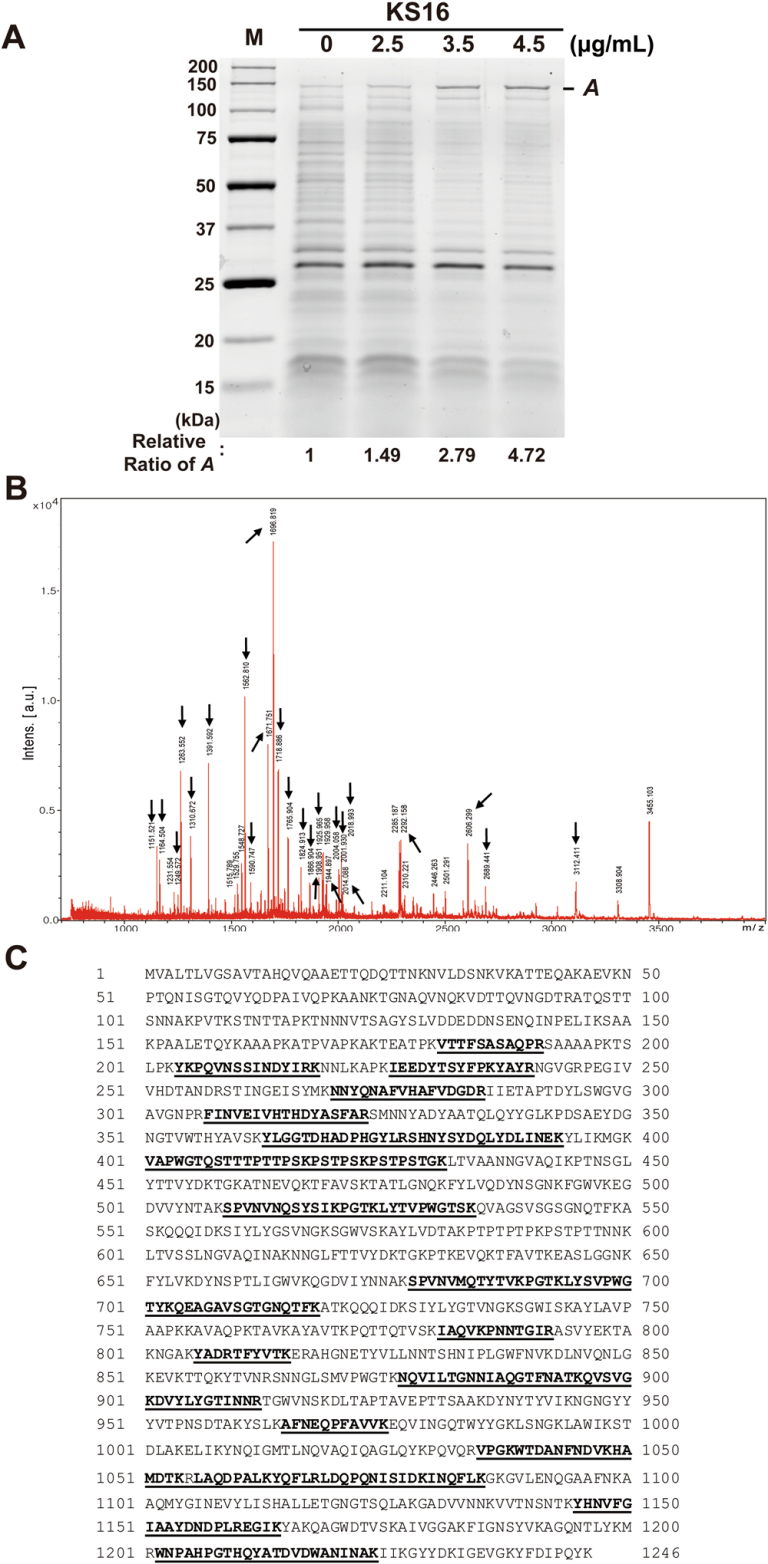
Characterization of protein affected by **KS16**. (**A**) Analysis of total cellular proteins from ATCC 25923 cells (1 × 10^9^) treated with different concentrations of **KS16**. Total cellular proteins were resolved on 12% TGX stain-free gels and imaged. The ratio of *A* was calculated using Image Lab Software (Bio-Rad). One of the representative data from n = 3 was shown. (**B**) MALDI-TOF/MS spectrum of band *A*. Arrows indicate the peptide fragments obtained. (**C**) Identification of autolysin protein. Analysis of peptide fragments using Mascot and Swiss-Prot identified that peptides sequences are matching to autolysin protein derived from *Staphylococcus aureus subsp*. *aureus* CIGC341D. Amino acid sequences were taken from NCBIprot database (GenBank: EHT86028.1). Bold underlined sequences (29% coverage) represent peptides matched with autolysin. All images were processed in Adobe Illustrator CS6 (v16.0.0, Adobe Systems Inc., USA).

Because **KS16** showed the synergy to antibiotics modulating protein synthesis (Fig. 9) we assumed that the protein composition in *S*. *aureus* cells is changed by **KS16**. To verify this hypothesis, total proteins from *S*. *aureus* cultures treated with **KS16** of sublethal concentrations (0 to 5 μg/mL) were compared to that of control sample. Interestingly, we found that a protein band migrating ~150 kDa (band ***A***) on SDS-PAGE gel was increased to 4.72-fold by **KS16** with concentration-dependent manner (Fig. 10A). To characterize the protein MALDI-TOF/peptide mass fingerprinting (PMF) was further performed (Fig. 10B). By Mascot and Swiss-Prot analysis of trypsin-treated fragments the protein from band ***A*** was denoted as mannosyl-glycoprotein endo-β-N-acetylglucosamidase (EC 3.2.1.96), also known as autolysin (Atl) by sequence similarity (Fig. 10C). The Atl is a bifunctional protein originally produced as a 138-kDa precursor form and processed to generate mature 62-kDa amidase (AM) and 51-kDa glucosamidase (GM) domains by sequencially^56–58^.

In functional aspects, Atl is known as a peptidoglycan hydrolase involved in bacterial cell wall degradation through trimming of peptidoglycan and the separation of the daughter cells after division^59,60^. Moreover, the protein is involved in the initial perforation of the cell wall during the autolysis of penicillin-treated *S*. *aureus*^61^. Therefore, our results suggest that **KS16** seems to function as an anti-*S*. *aureus* by activating the degradation of peptidoglycan layers of target cells and/or perforation of the cell wall. However, future studies are required to understand how **KS16** showed the synergistic activity to antibiotics related to protein synthesis and how **KS16** selectively affected to the viability of *Staphylococcus* species. For this purpose, profiling analyses of both total RNAs and proteins by high-throughput techniques such as RNA-sequencing and proteomics are required. Such studies will broaden the usage of heterocyclic oxindole compounds as new lead molecules with greater activity and specificity for the eradication of *Staphylococcus* species.

### Cytotoxicity of KS16

**Figure 11:**
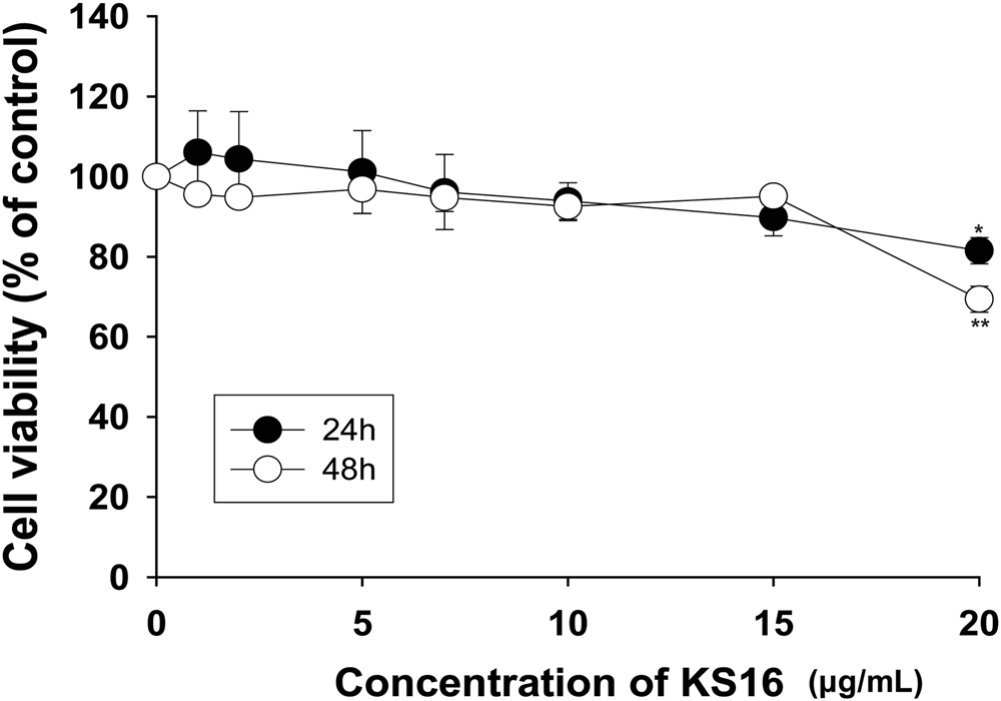
Cytotoxicity of assays. Cell viability of human fibroblasts presented as percentage of control after 24 to 48 h exposure to different concentrations of **KS16**. The data represent the average values of n = 3. * and ** Indicate P < 0.05 and P < 0.01, respectively. The image was processed in Adobe Illustrator CS6 (v16.0.0, Adobe Systems Inc., USA).

For the evaluation of cytotoxicity of **KS16**, human skin fibroblast cell (CRL 2097) was selected in the aspect that one of colonization sites for *S*. *aureus* is skin tissues^62^. The cytotoxicity of **KS16** with a range of increasing concentrations (0 to 20 μg/mL) after 24 and 48 h of incubation was determined using the CCK-8 assay. Data showed that **KS16** at MIC level (4.5 to 6.4 μg/mL) against *Staphylococcus* species including MRSAs did not exhibit any cytotoxicity (Fig. 11). Moreover, even at 20 μg/mL, which is 3.1- to 4.4-fold of MIC value did not exhibit significant cytotoxicity. This indicates that **KS16** could lead to possible non-toxic anti-MRSA application for skin infection.

## Conclusion

In summary, we have designed and synthesized diverse oxindoles bearing 3-heterocycles by indium (III)-catalysed reaction of 3-diazoindolin-2-ones and indoles, 1-methylpyrrole, or thiophene. Among the synthesized compounds **KS15**, **KS16**, **KS17**, **KS19**, and **KS20** were the most active compounds with high specificity against various *Staphylococcus* species including MRSA with equivalent or superior activity to streptomycin. The study on synergistic activity of **KS16** with existing antibiotics indicates that **KS16** increased the susceptibility of *S*. *aureus* to ciprofloxacin, gentamicin, kanamycin, and streptomycin. Among them, gentamicin and kanamycin exhibited a synergistic combination with **KS16** to *S*. *aureus*. Moreover, the **KS16** increased the cellular level of autolysin (Atl) protein and expressed the antibacterial activity. Finally, the treatment of **KS16** to skin fibroblast cells at the MIC levels used for killing *Staphylococcus* species was non-toxic. All our findings suggest that our newly developed heterocyclic oxindoles are valuable lead antibacterial agents in the eradication of multidrug-resistant *Staphylococcus* species.

## Supplementary information

Supporting Information

## References

[1] President of the General Assembly of the United Nations. Press release: high-level meeting on antimicrobial resistance, http://www.un.org/pga/71/2016/09/21/press-release-hl-meeting-on-antimicrobial-resistance/ (2016).

[2] Brown ED, Wright GD (2016). Antibacterial drug discovery in the resistance era. Nature.

[3] Diekema DJ (2001). Survey of infections due to Staphylococcus species: frequency of occurrence and antimicrobial susceptibility of isolates collected in the United States, Canada, Latin America, Europe, and the Western Pacific region for the SENTRY Antimicrobial Surveillance Program, 1997–1999. Clin. Infect. Dis..

[4] O’Neill, J. Tackling drug-resistant infections globally: final report and recommendations. In the *Review on Antimicrobial Resistance*, https://amr-review.org/sites/default/files/160525_Final%20paper_with%20cover.pdf (2016).

[5] Lowy FD (2013). Antimicrobial resistance: the example of Staphylococcus aureus. J. Clin. Invest..

[6] Witte W (2009). Community-acquired methicillin-resistant Staphylococcus aureus: what do we need to know?. Clin. Microbiol. Infect..

[7] Lowy FD (1998). Staphylococcus aureus infections. N. Engl. J. Med..

[8] Zinkernagel AS (2008). Significance of Staphylococcus lugdunensis bacteremia: report of 28 cases and review of the literature. Infection.

[9] National Nosocomial Infections Surveillance System. National Nosocomial Infections Surveillance (NNIS) System Report, data summary from January 1992 through June 2004. *Am*. *J*. *Infect*. *Control*. **32**, 470–485 (2004). 10.1016/S0196655304005425 15573054

[10] Vuong C, Otto M (2002). Staphylococcus epidermidis infections. Microbes Infect..

[11] Raz R, Colodner R, Kunin CM (2005). Who are you-Staphylococcus saprophyticus?. Clin. Infect. Dis..

[12] Chambers HF (2001). The changing epidemiology of Staphylococcus aureus?. Emerg. Infect. Dis..

[13] Raad I, Alrahwan A, Rolston K (1998). Staphylococcus epidermidis: emerging resistance and need for alternative agents. Clin. Infect. Dis..

[14] Parker D (2018). A live vaccine to Staphylococcus aureus infection. Virulence.

[15] Zhang X (2018). Master mechanisms of Staphylococcus aureus: consider its excellent protective mechanisms hindering vaccine development!. Microbiol. Res..

[16] Zha G (2019). Discovery of novel arylethenesulfonyl fluorides as potential candidates against methicillin-resistant of Staphylococcus aureus (MRSA) for overcoming multidrug resistance of bacterial infections. Eur. J. Med. Chem..

[17] Ravindar L (2018). Aryl fluosulfate analogues as potent antimicrobial agents: SAR, cytotoxicity and docking studies. Bioorg. Chem..

[18] Manukumar HM (2017). Novel T-C@AgNPs mediated biocidal mechanism against biofilm associated methicillin-resistant Staphylococcus aureus (Bap-MRSA) 090, cytotoxicity and its molecular docking studies. Med. Chem. Comm..

[19] Mohammed YHE (2018). Vision for medicine: Staphylococcus aureus biofilm war and unlocking key’s for anti-biofilm drug development. Microb. Pathog..

[20] Rakesh KP (2018). Combating a master manipulator: Staphylococcus aureus immunomodulatory molecules as targets for combinatorial drug discovery. ACS Comb. Sci..

[21] Donlan RM (2002). Biofilms: Microbial Life on Surfaces. Emerg. Infect. Dis..

[22] Bansal T (2007). Differential effects of epinephrine, norepinephrine, and indole on Escherichia coli O157:H7 chemotaxis, colonization, and gene expression. Infect. Immun..

[23] Lee J, Jayaraman A, Wood TK (2007). Indole is an inter-species biofilm signal mediated by SdiA. BMC Microbiol..

[24] Oh S, Go GW, Mylonakis E, Kim Y (2012). The bacterial signalling molecule indole attenuates the virulence of the fungal pathogen Candida albicans. J. Appl. Microbiol..

[25] Lee JH (2015). The multifaceted roles of the interspecies signalling molecule indole in Agrobacterium tumefaciens. Environ. Microbiol..

[26] Stewart PS, Costerton JW (2001). Antibiotic resistance of bacteria in biofilms. Lancet.

[27] Ferrer MD (2017). Effect of antibiotics on biofilm inhibition and induction measured by real-time cell analysis. J. Appl. Microbiol..

[28] Rakesh KP (2018). Promising bactericidal approach of dihydrazone analogues against bio-film forming Gram-negative bacteria and molecular mechanistic studies. RSC Adv..

[29] Zhang X (2018). Role of BP*C@AgNPs in Bap-dependent multicellular behavior of clinically important methicillin-resistant Staphylococcus aureus (MRSA) biofilm adherence: A key virulence study. Microb. Pathog..

[30] Rui L, Reardon KF, Wood TK (2005). Protein engineering of toluene ortho-monooxygenase of Burkholderia cepacia G4 for regiospecific hydroxylation of indole to form various indigoid compounds. Appl. Microbiol. Biotechnol..

[31] Lee J (2007). Enterohemorrhagic Escherichia coli biofilms are inhibited by 7-hydroxyindole and stimulated by isatin. Appl. Environ. Microbiol..

[32] Lee JH (2016). Halogenated indoles eradicate bacterial persister cells and biofilms. AMB Expr..

[33] Dreifuss AA (2010). Antitumoral and antioxidant effects of a hydroalcoholic extract of cat’s claw (Uncaria tomentosa) (Willd. Ex Roem. & Schult) in an *in vivo* carcinosarcoma model. J. Ethnopharmacol..

[34] Giménez DG (2010). Cytotoxic effect of the pentacyclic oxindole alkaloid mitraphylline isolated from Uncaria tomentosa bark on human ewing’s sarcoma and breast cancer cell lines. Planta. Medica..

[35] Guan H (2004). Design and synthesis of aminopropyl tetrahydroindole-based indolin-2-ones as selective and potent inhibitors of Src and Yes tyrosine kinase. Bioorg. Med. Chem. Lett..

[36] Rindhe SS, Karale BK, Gupta RC (2011). Synthesis, antimicrobial and antioxidant activity of some oxindoles. Indian J. Pharm. Sci..

[37] Singh SB (2013). A new eco-friendly strategy for the synthesis of novel antimicrobial spiro-oxindole derivatives via supramolecular catalysis. Supramol. Chem..

[38] Singh H (2014). Ultrasound promoted one pot synthesis of novel fluorescent triazolyl spirocyclic oxindoles using DBU based task specific ionic liquids and their antimicrobial activity. Eur. J. Med. Chem..

[39] Sayed M, Kamal El-Dean AM, Ahmed M, Hassanien R (2018). Synthesis of some heterocyclic compounds derived from indole as antimicrobial agents. Synth. Comm..

[40] Bonev B, Hooper J, Parisot J (2008). Principles of assessing bacterial susceptibility to antibiotics using the agar diffusion method. J. Antimicrob. Chemother..

[41] Abd Al-Abbas MJ (2012). Antimicrobial susceptibility of Enterococcus faecalis and a novel Planomicrobium isolate of bacteremia. Int. J. Med. Med. Sci..

[42] Wiegand I, Hilpert K, Hancock RE (2008). Agar and broth dilution methods to determine the minimal inhibitory concentration (MIC) of antimicrobial substances. Nat. Protoc..

[43] Odds FC (2003). Synergy, antagonism, and what the chequerboard puts between them. J. Chemother..

[44] Shevchenko ON (1996). Linking genome and proteome by mass spectrometry: large-scale identification of yeast proteins from two dimensional gels. Proc. Natl. Acad. Sci. USA.

[45] Fernandez J (1998). Routine identification of proteins from sodium dodecyl sulfate-polyacrylamide gel electrophoresis (SDS-PAGE) gels or polyvinyl difluoride membranes using matrix assisted laser desorption/ionization-time of flight-mass spectrometry (MALDI-TOF-MS). Electrophoresis.

[46] Lee J (2012). A novel small molecule facilitates the reprogramming of human somatic cells into a pluripotent state and supports the maintenance of an undifferentiated state of human pluripotent stem cells. Angew. Chem. Int. Ed..

[47] Baldan R (2014). Adaptation of Pseudomonas aeruginosa in Cystic Fibrosis airways influences virulence of Staphylococcus aureus *in vitro* and murine models of co‐infection. PLoS One.

[48] Dalton T (2011). An *in vivo* polymicrobial biofilm wound infection model to study interspecies interactions. PLoS One.

[49] Dowd SE (2008). Survey of bacterial diversity in chronic wounds using pyrosequencing, DGGE, and full ribosome shotgun sequencing. BMC Microbiol..

[50] Joo HS, Otto M (2002). Molecular basis of *in vivo* biofilm formation by bacterial pathogens. Chem. Biol..

[51] Michelsen CF (2014). Staphylococcus aureus alters growth activity, autolysis, and antibiotic tolerance in a human host‐adapted Pseudomonas aeruginosa lineage. J. Bacteriol..

[52] Pastar I (2013). Interactions of methicillin resistant Staphylococcus aureus USA300 and Pseudomonas aeruginosa in polymicrobial wound infection. PLoS One.

[53] Seth AK (2014). Impact of a novel, antimicrobial dressing on *in vivo*, Pseudomonas aeruginosa wound biofilm: Quantitative comparative analysis using a rabbit ear mode. Wound Repair Regen..

[54] Brewer NS (1977). Antimicrobial agents-Part II. The aminoglycosides: streptomycin, kanamycin, gentamicin, tobramycin, amikacin, neomycin. Mayo. Clin. Proc..

[55] Cottagnoud P (2003). Vancomycin acts synergistically with gentamicin against penicillin-resistant pneumococci by increasing the intracellular penetration of gentamicin. Antimicrob. Agents Chemother..

[56] Houston P (2011). Essential role for the major autolysin in the fibronectin-binding protein-mediated Staphylococcus aureus biofilm phenotype. Infect. Immun..

[57] Grilo IR (2014). The glucosaminidase domain of Atl-the major Staphylococcus aureus autolysin- has DNA-binding activity. Microbiologyopen..

[58] Foster SJ (1995). Molecular characterization and functional analysis of the major autolysin of Staphylococcus aureus 8325/4. J. Bacteriol..

[59] Oshida T (1995). A Staphylococcus aureus autolysin that has an N-acetylmuramoyl-L-alanine amidase domain and an endo-*β*-N-acetylglucosaminidase domain: cloning, sequence analysis, and characterization. Proc. Natl. Acad. USA.

[60] Sugai M (1995). Identification of endo-*β*-N-acetylglucosaminidase and N-acetylmuramyl-L-alanine amidase as cluster-dispersing enzymes in *Staphylococcus aureus*. J. Bacteriol..

[61] Sugai, M. *et al*. Localized perforation of the cell wall by a major autolysin: atl gene products and the onset of penicillin-induced lysis of Staphylococcus aureus. *J*. *Bacteriol*. **179**, 2958–2962. 10.1128/jb.179.9.2958-2962.1997 PMC179060 9139914

[62] Tong SY (2015). Staphylococcus aureus infections: epidemiology, pathophysiology, clinical manifestations, and management. Clin. Microbiol. Rev..

