# Supplementary material for: Design, synthesis, and discovery of novel oxindoles bearing 3-heterocycles as species-specific and combinatorial agents in eradicating Staphylococcus species
Source: Sci Rep. 2019 Sep 16;9:8012. doi: 10.1038/s41598-019-44304-1 (PMC6746789; doi:10.1038/s41598-019-44304-1)
Supplement: Supplementary file 1 — Supporting Information [file 41598_2019_44304_MOESM1_ESM.pdf]

Design, synthesis, and discovery of novel oxindoles bearing 3-heterocycles as species-specific and combinatorial agents in eradicating *Staphylococcus* species

Jonghoon Shin[1,+], Krishna Bahadur Somai Magar[2,+], Jungwoon Lee[3],

Kwang-sun Kim[1,*] & Yong Rok Lee[2,*]

[1]Department of Chemistry and Chemistry Institute for Functional Materials, Pusan National University, Busan 46241, Republic of Korea

[2]School of Chemical Engineering, Yeungnam University, Gyeongsan 38541, Republic of Korea

[3]Environmental Disease Research Center, Korea Research Institute of Bioscience & Biotechnology, 125 Gwahak-ro, Yuseong-gu, Daejeon 34141, Republic of Korea

*Correspondence and requests for materials should be addressed to Y.R.L. (E-mail:yrlee@yu.ac.kr) or K.S.K. (E-mail:kwangsun.kim@pusan.ac.kr)

[+]These authors contributed equally to this work

SUPPORTING INFORMATION (SI)

TABLE OF CONTENTS

SI1. ^{1}H NMR and ^{13}C NMR Spectra of Synthesized Compounds KS1-KS20 …………...S2-S21

SI2. Copy of GC Spectrum of compound KS16 ………………………………………………S22

^{1}H NMR and ^{13}C NMR Spectra of Synthesized Compounds KS1-KS20

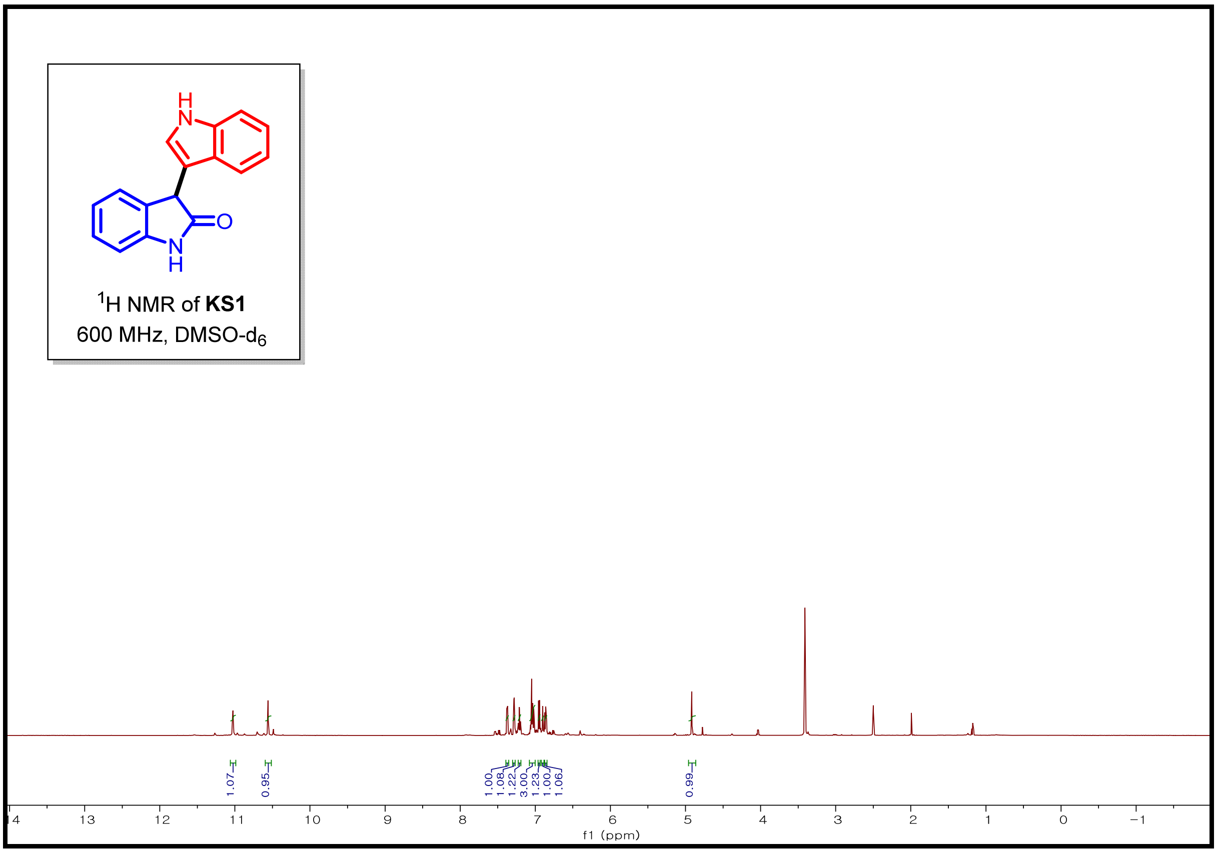

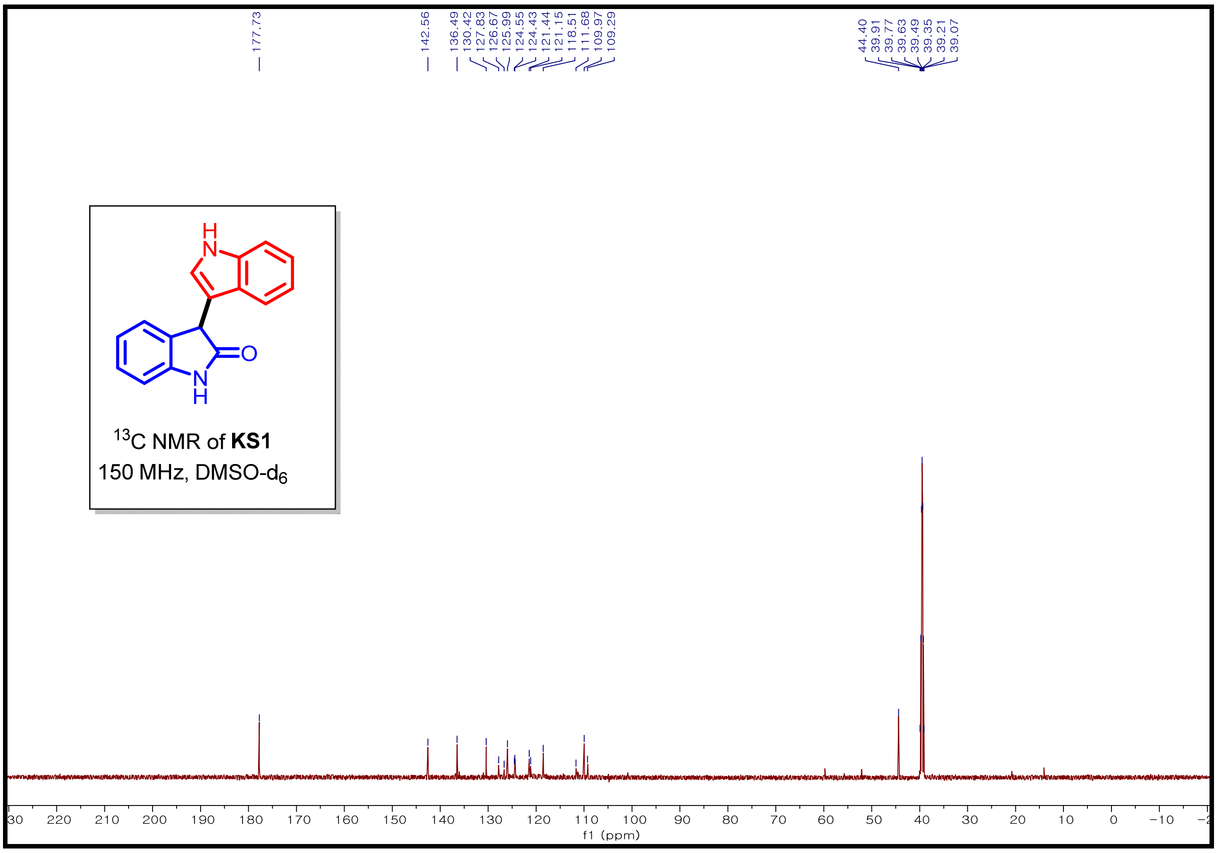

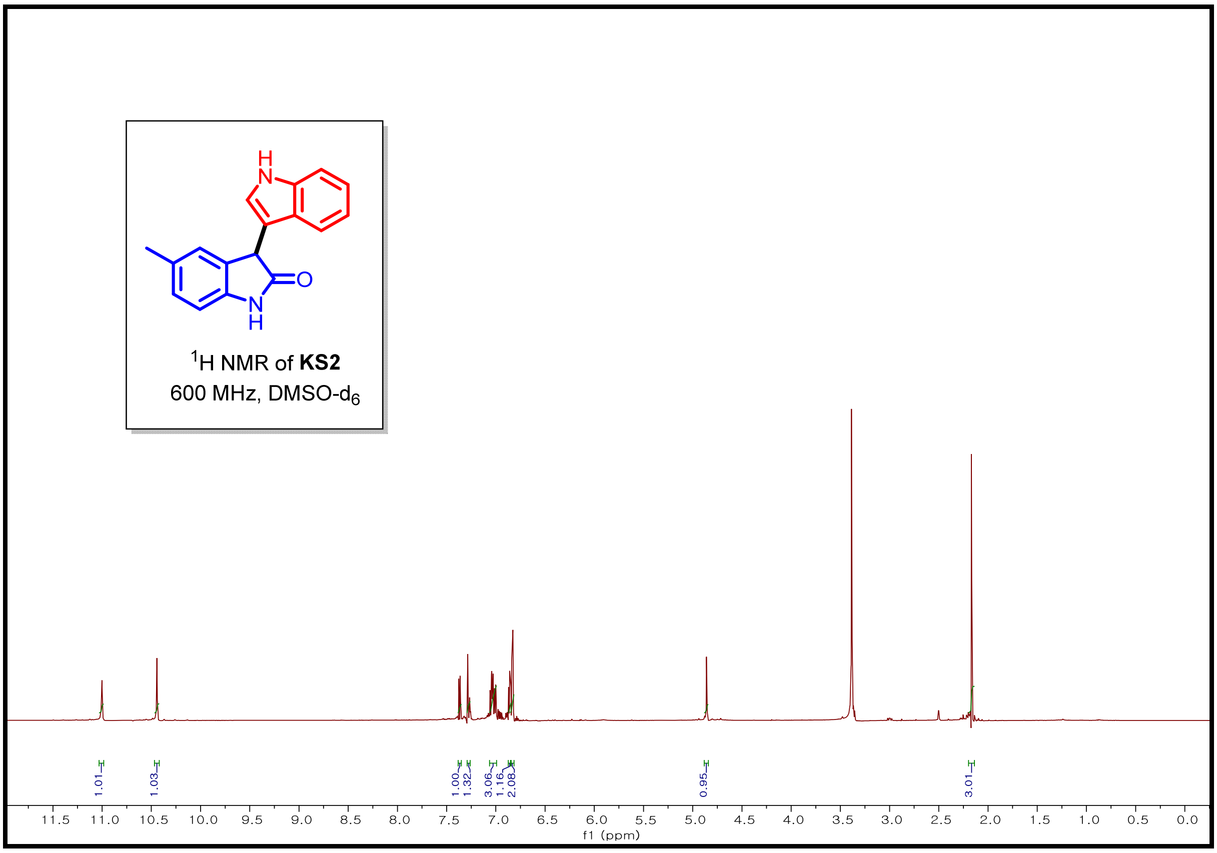

1H NMR of KS2
600 MHz, DMSO-d6
1.01
1.03
1.00
1.32
3.06
1.16
2.08
0.95
3.01
11.5 11.0 10.5 10.0 9.5 9.0 8.5 8.0 7.5 7.0 6.5 6.0 5.5 5.0 4.5 4.0 3.5 3.0 2.5 2.0 1.5 1.0 0.5 0.0
f1 (ppm)

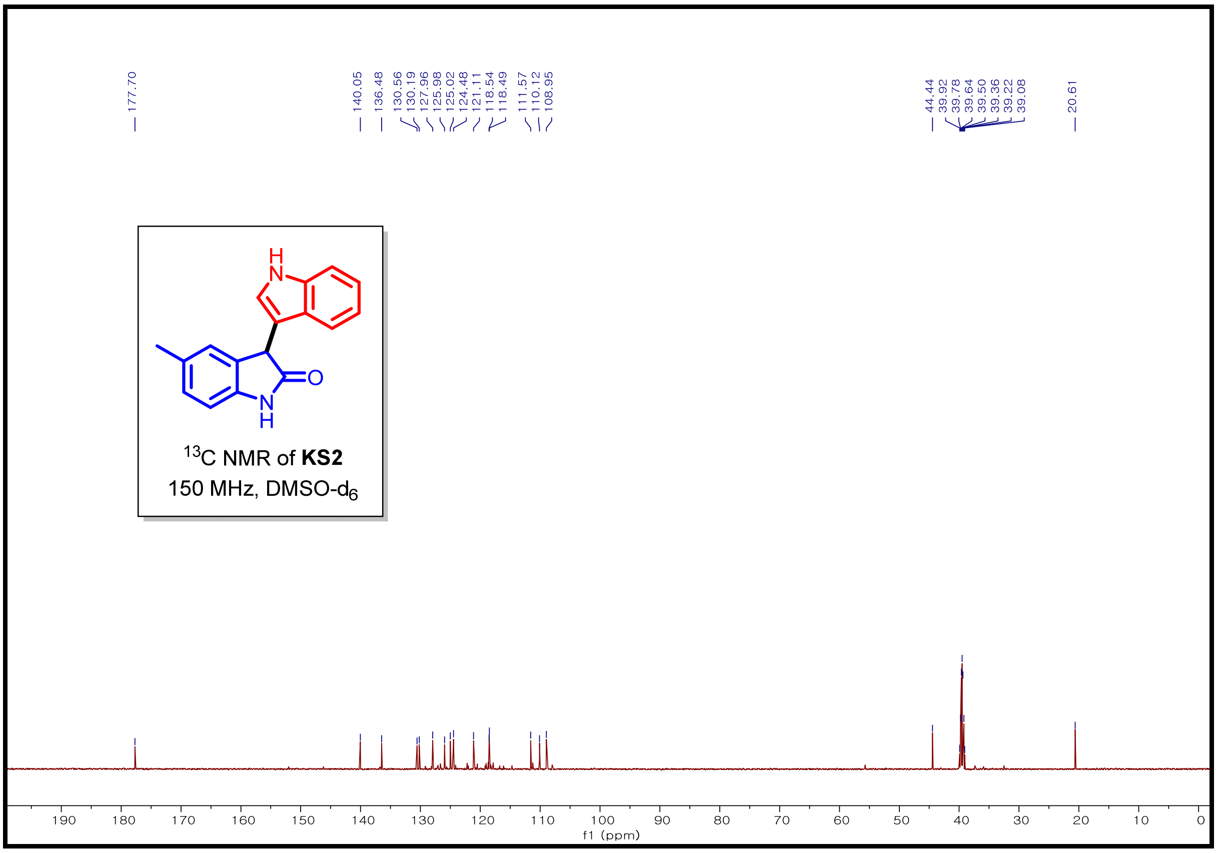

177.70
140.05
136.48
130.56
130.19
127.96
125.98
125.02
124.48
121.11
118.54
118.49
111.57
110.12
108.95
44.44
39.92
39.78
39.64
39.50
39.36
39.22
39.08
20.61
13C NMR of KS2
150 MHz, DMSO-d6
190 180 170 160 150 140 130 120 110 100 90 80 70 60 50 40 30 20 10 0
f1 (ppm)

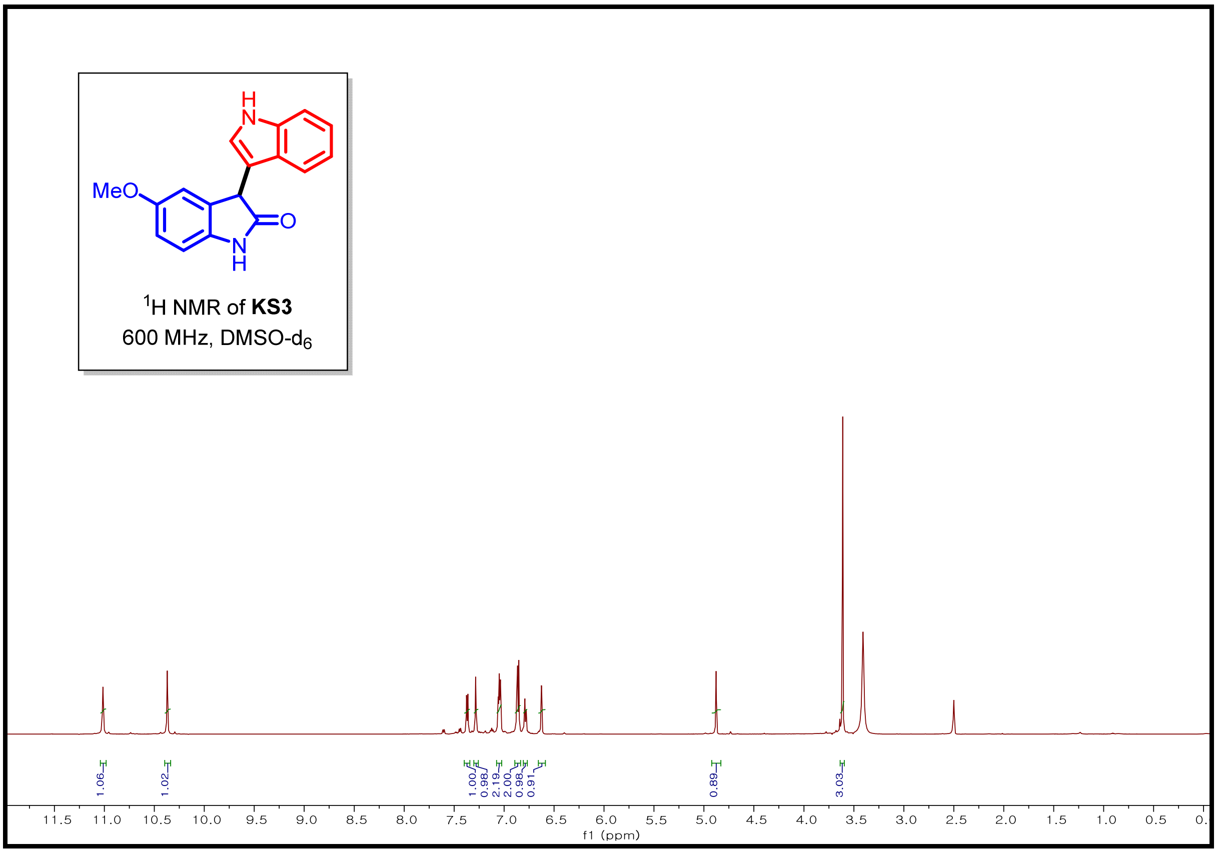
MeO
H
N
O
N
H
1H NMR of KS3
600 MHz, DMSO-d6
1.06
1.02
1.00
0.98
2.19
2.00
0.98
0.91
0.89
3.03
11.5 11.0 10.5 10.0 9.5 9.0 8.5 8.0 7.5 7.0 6.5 6.0 5.5 5.0 4.5 4.0 3.5 3.0 2.5 2.0 1.5 1.0 0.5
f1 (ppm)

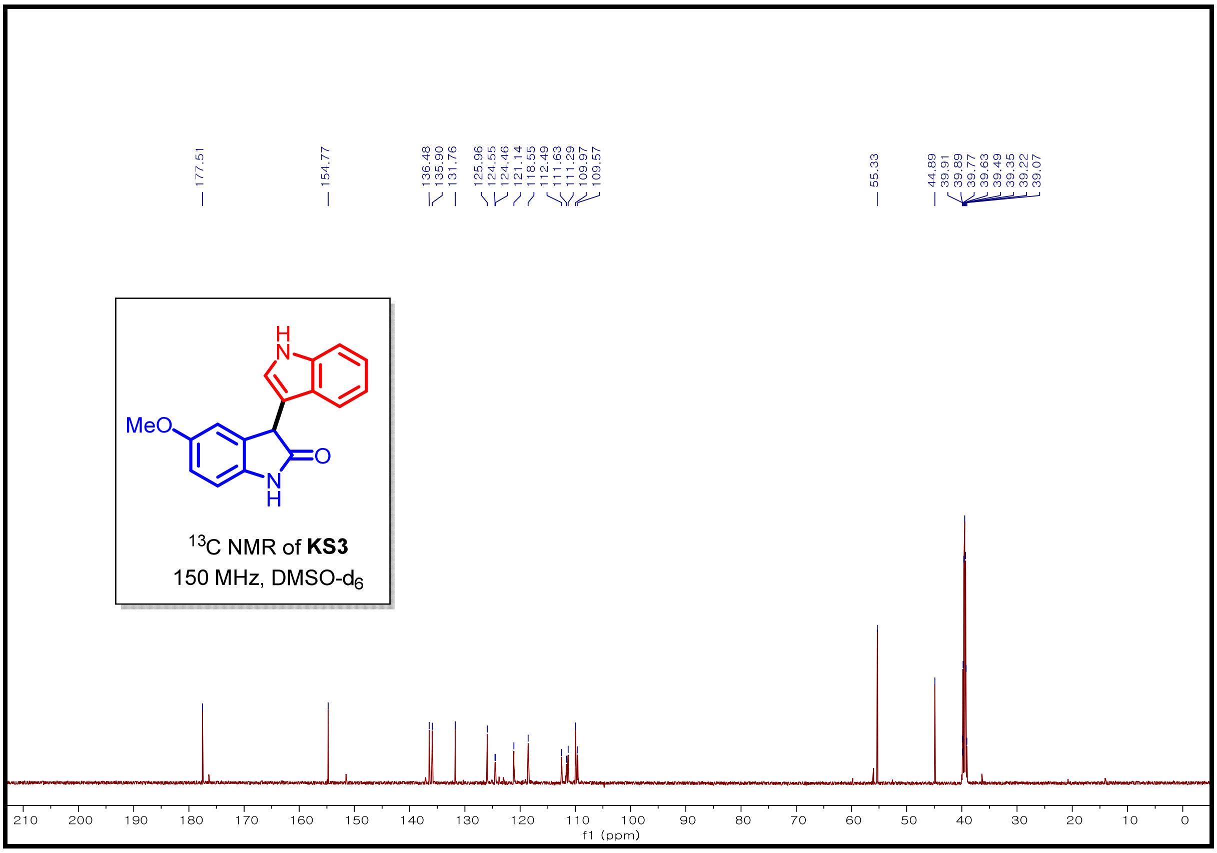
177.51
154.77
136.48
135.90
131.76
125.96
124.55
124.46
121.14
118.55
112.49
111.63
111.29
109.97
109.57
55.33
44.89
39.91
39.89
39.77
39.63
39.49
39.35
39.22
39.07
MeO
H
N
O
N
H
13C NMR of KS3
150 MHz, DMSO-d6
210 200 190 180 170 160 150 140 130 120 110 100 90 80 70 60 50 40 30 20 10 0
f1 (ppm)

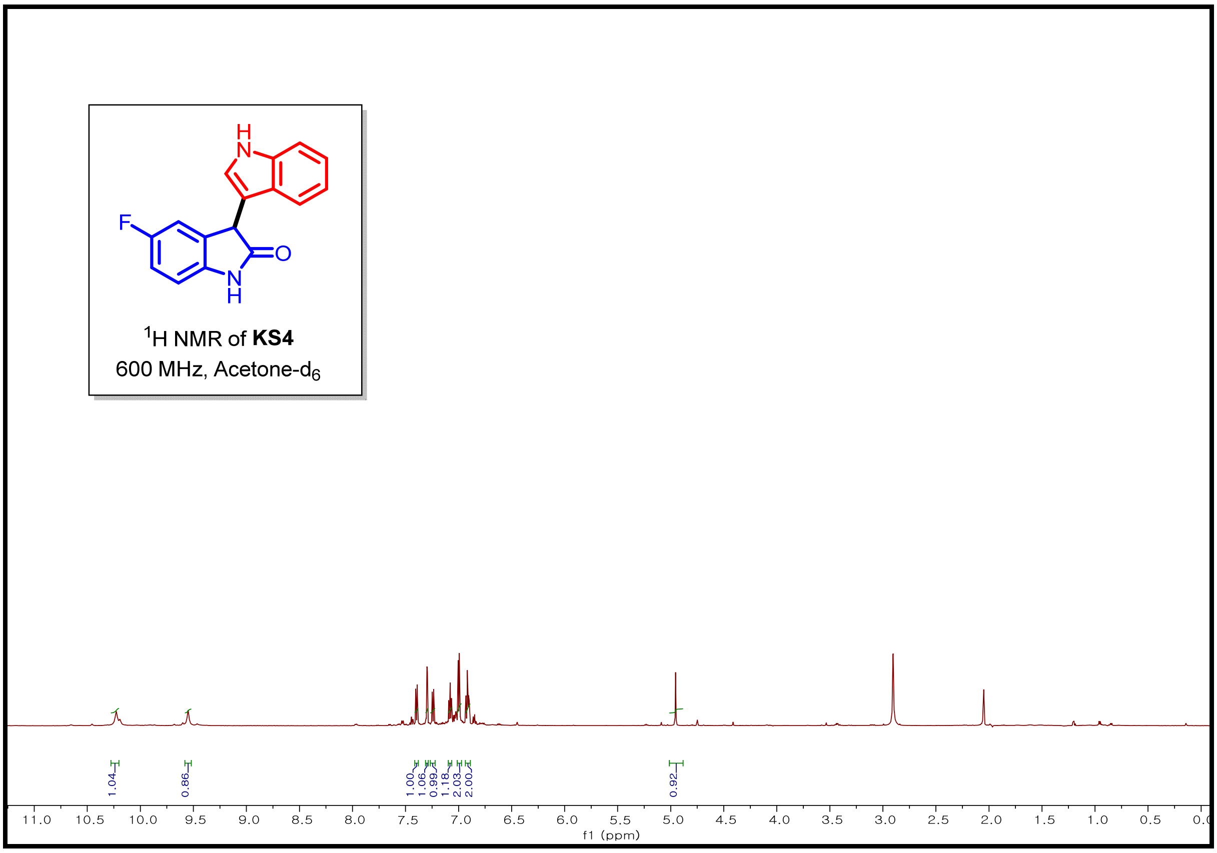

^{1}H NMR of **KS4**
600 MHz, Acetone-d$_6$
1.04
0.86
1.00
1.06
0.99
1.18
2.03
2.00
0.92
f1 (ppm)

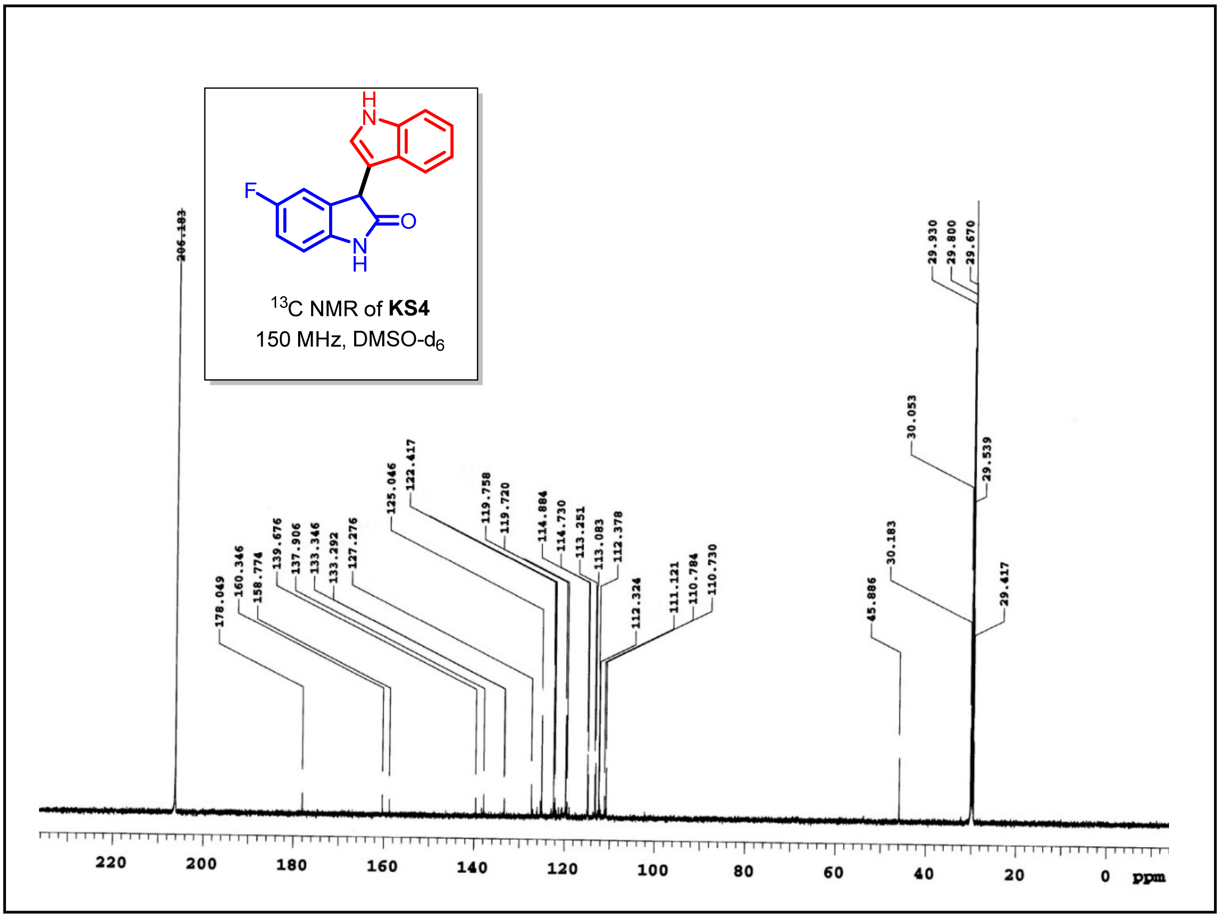

^{13}C NMR of **KS4**
150 MHz, DMSO-d$_6$
206.183
178.049
160.346
158.774
139.676
137.906
133.346
133.292
127.276
125.046
122.417
119.758
119.720
114.884
114.730
113.251
113.083
112.378
112.324
111.121
110.784
110.730
45.886
30.183
30.053
29.930
29.800
29.670
29.539
29.417
ppm

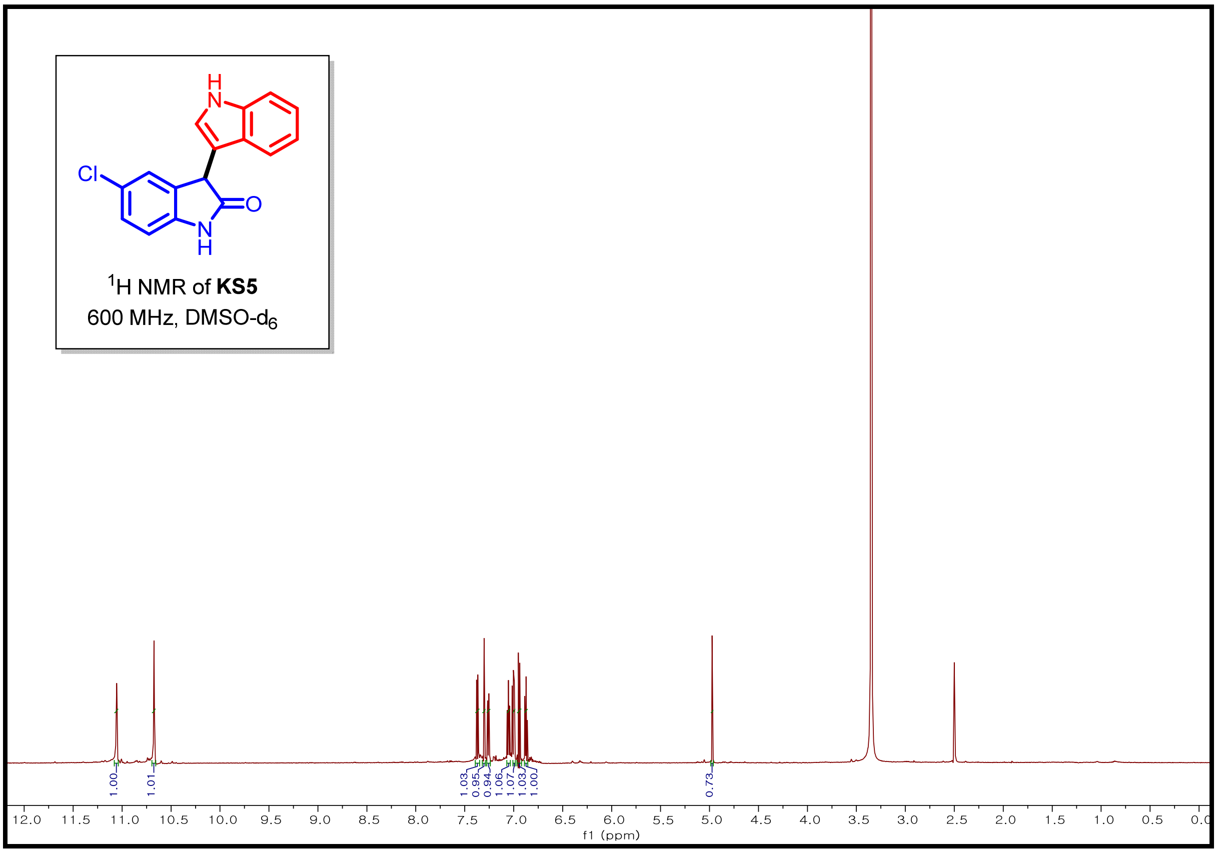

Cl
H
N
O
N
H
1H NMR of KS5
600 MHz, DMSO-d6
1.00
1.01
1.03
0.95
0.94
1.06
1.07
1.03
1.00
0.73
f1 (ppm)

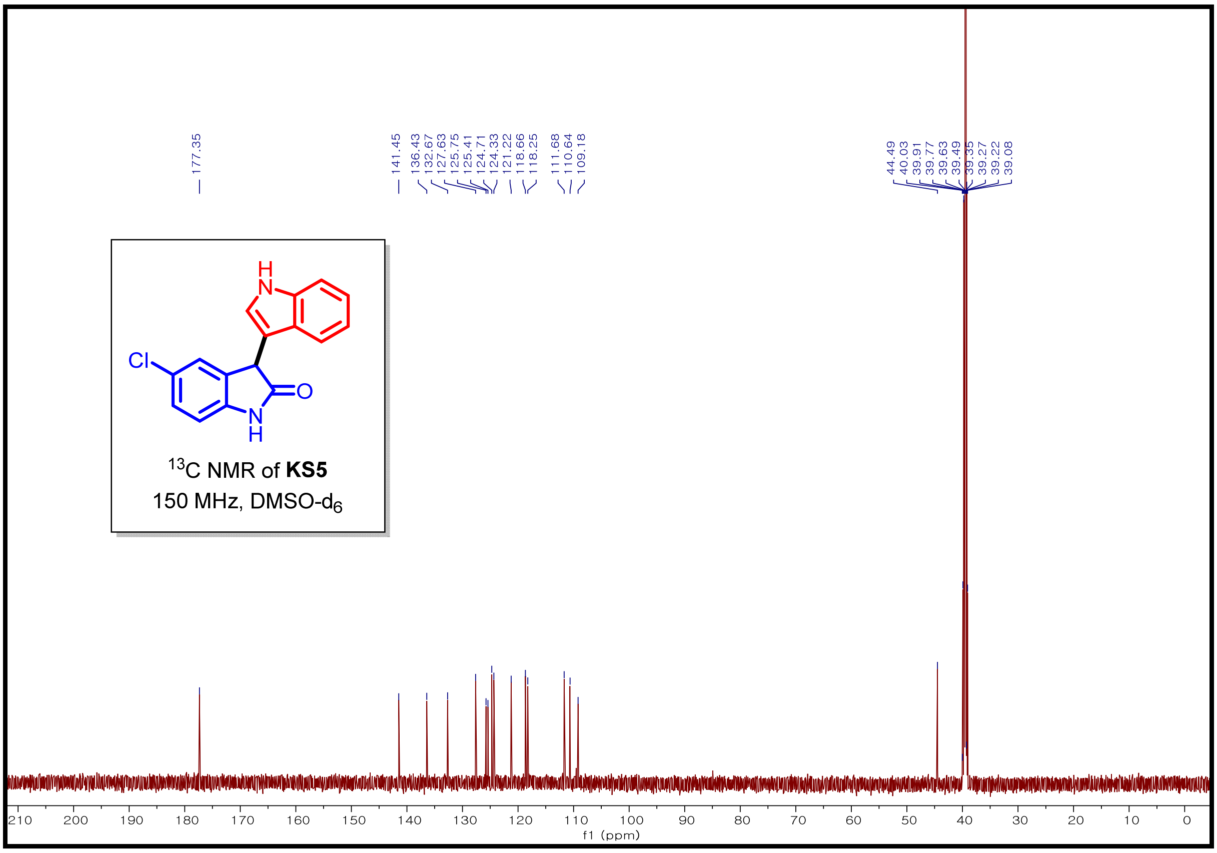

177.35
141.45
136.43
132.67
127.53
125.75
125.41
124.71
124.33
121.22
118.66
118.25
111.68
110.64
109.18
44.49
40.03
39.91
39.77
39.63
39.49
39.35
39.27
39.22
39.08
Cl
H
N
O
N
H
13C NMR of KS5
150 MHz, DMSO-d6
f1 (ppm)

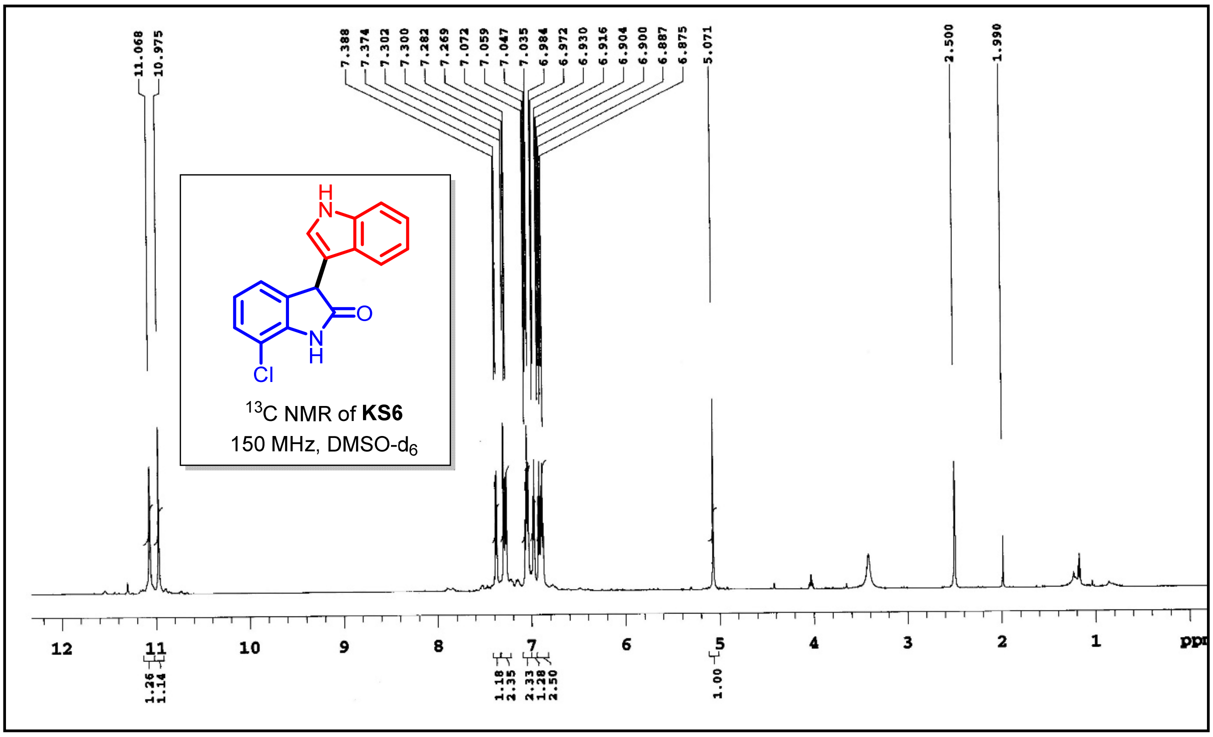
11.068
10.975
7.388
7.374
7.302
7.300
7.282
7.269
7.072
7.059
7.047
7.035
6.984
6.972
6.930
6.916
6.904
6.900
6.887
6.875
5.071
2.500
1.990
^{13}C NMR of **KS6**
150 MHz, DMSO-d_6
1.26
1.14
1.18
2.35
2.33
1.28
2.50
1.00
12
11
10
9
8
7
6
5
4
3
2
1
ppm

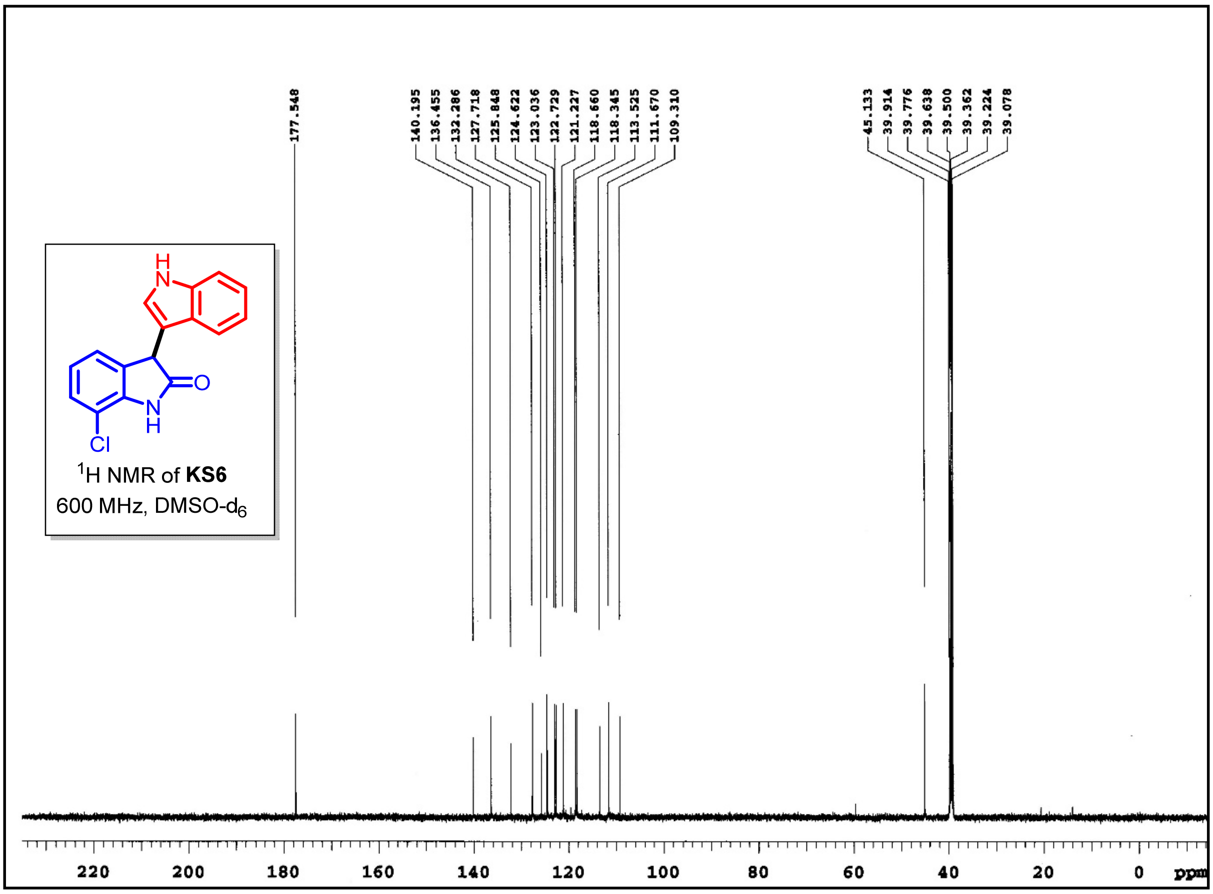
177.548
140.195
136.455
132.286
127.718
125.848
124.622
123.036
122.729
121.227
118.660
118.345
113.525
111.670
109.310
45.133
39.914
39.776
39.638
39.500
39.362
39.224
39.078
^{1}H NMR of **KS6**
600 MHz, DMSO-d_6
220
200
180
160
140
120
100
80
60
40
20
0
ppm

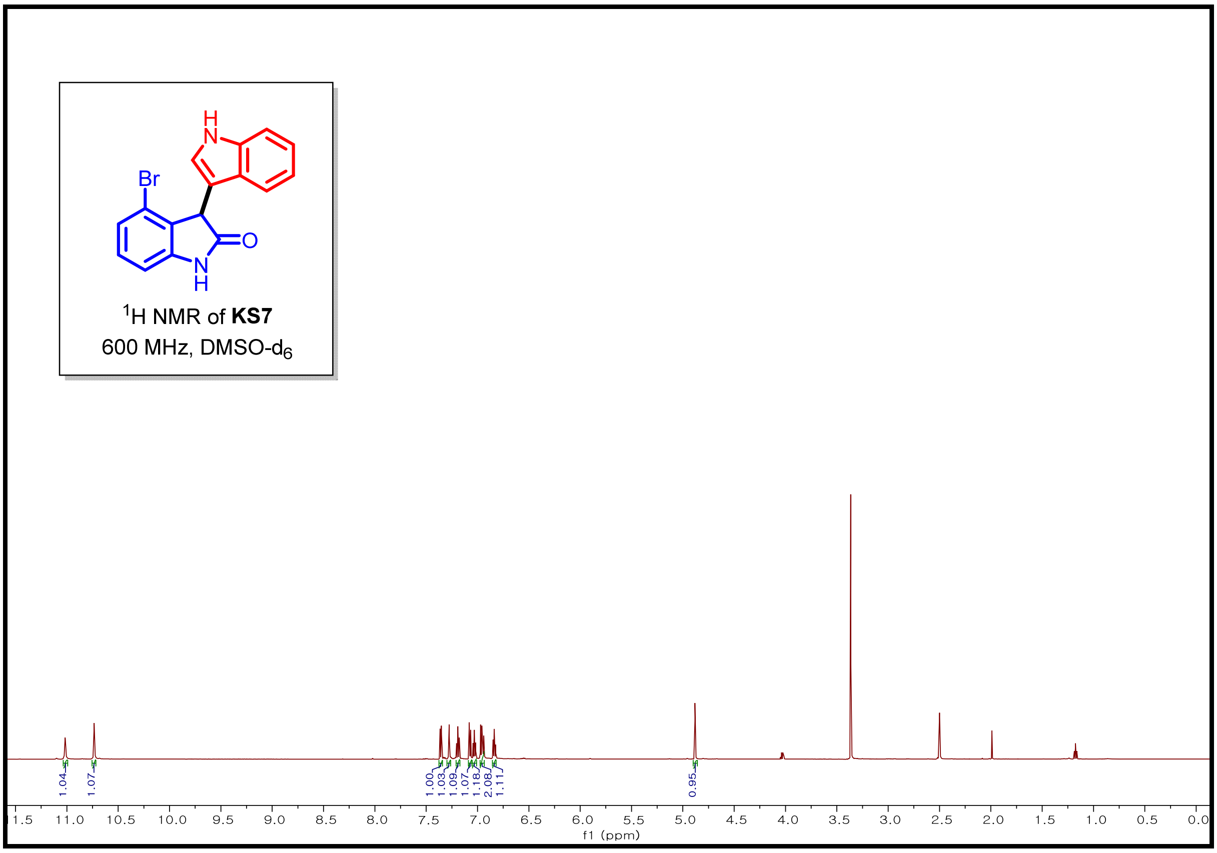

Br
H
N
O
N
H
1H NMR of KS7
600 MHz, DMSO-d6
1.04
1.07
1.00
1.03
1.09
1.07
1.18
2.08
1.11
0.95
f1 (ppm)

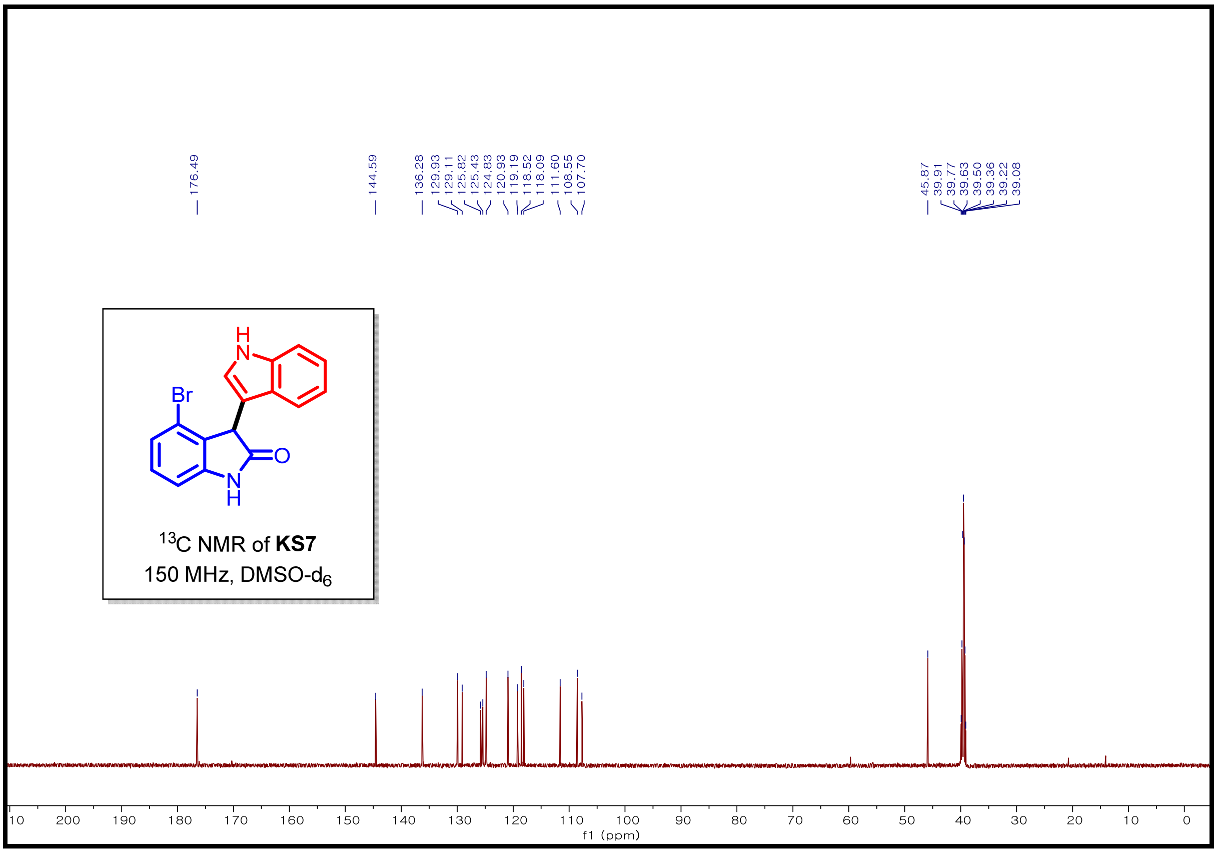

176.49
144.59
136.28
129.93
129.11
125.82
125.43
124.83
120.93
119.19
118.52
118.09
111.60
108.55
107.70
45.87
39.91
39.77
39.63
39.50
39.36
39.22
39.08
Br
H
N
O
N
H
13C NMR of KS7
150 MHz, DMSO-d6
f1 (ppm)

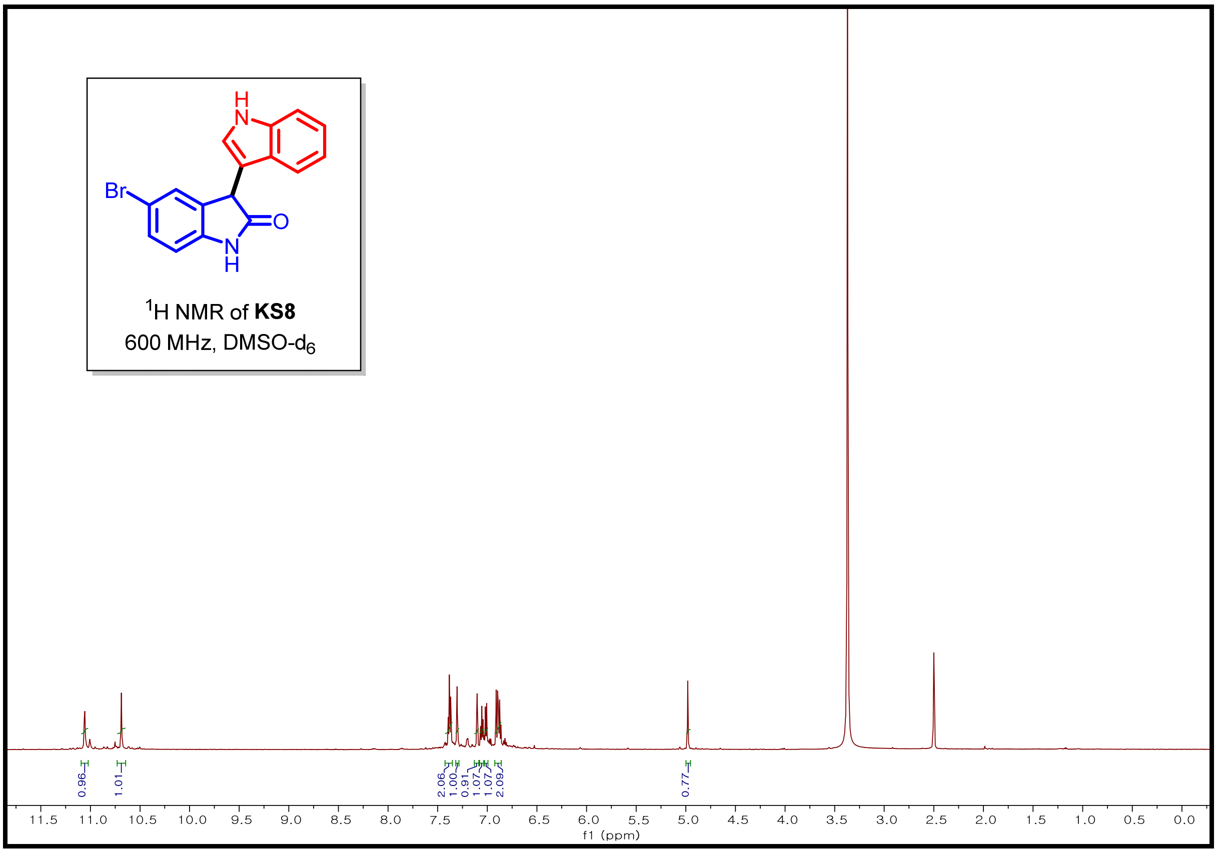

^{1}H NMR of KS8
600 MHz, DMSO-d6
Br
N H
O
0.96
1.01
2.06
1.00
0.91
1.07
1.07
1.07
2.09
0.77
f1 (ppm)

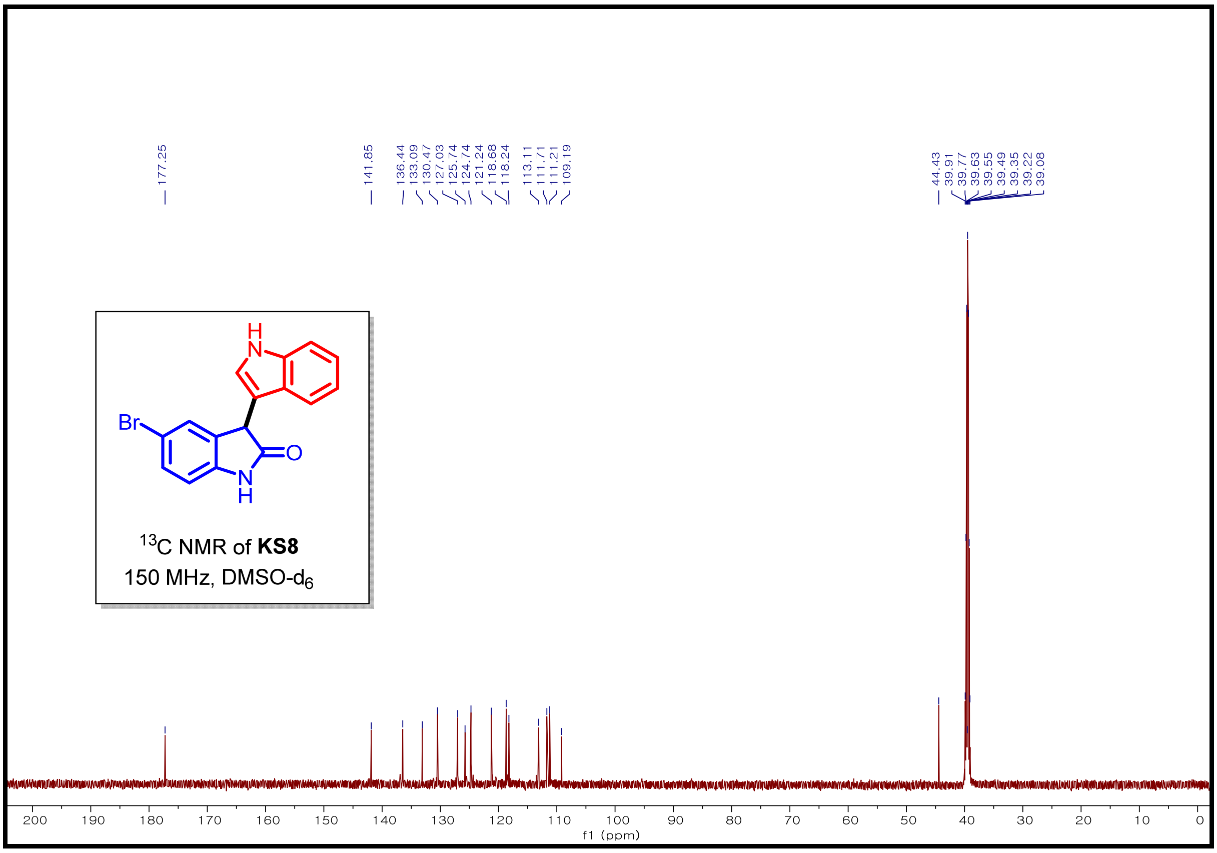

^{13}C NMR of KS8
150 MHz, DMSO-d6
Br
N H
O
177.25
141.85
136.44
133.09
130.47
127.03
125.74
124.74
121.24
118.68
118.24
113.11
111.71
111.21
109.19
44.43
39.91
39.77
39.63
39.55
39.49
39.35
39.22
39.08
f1 (ppm)

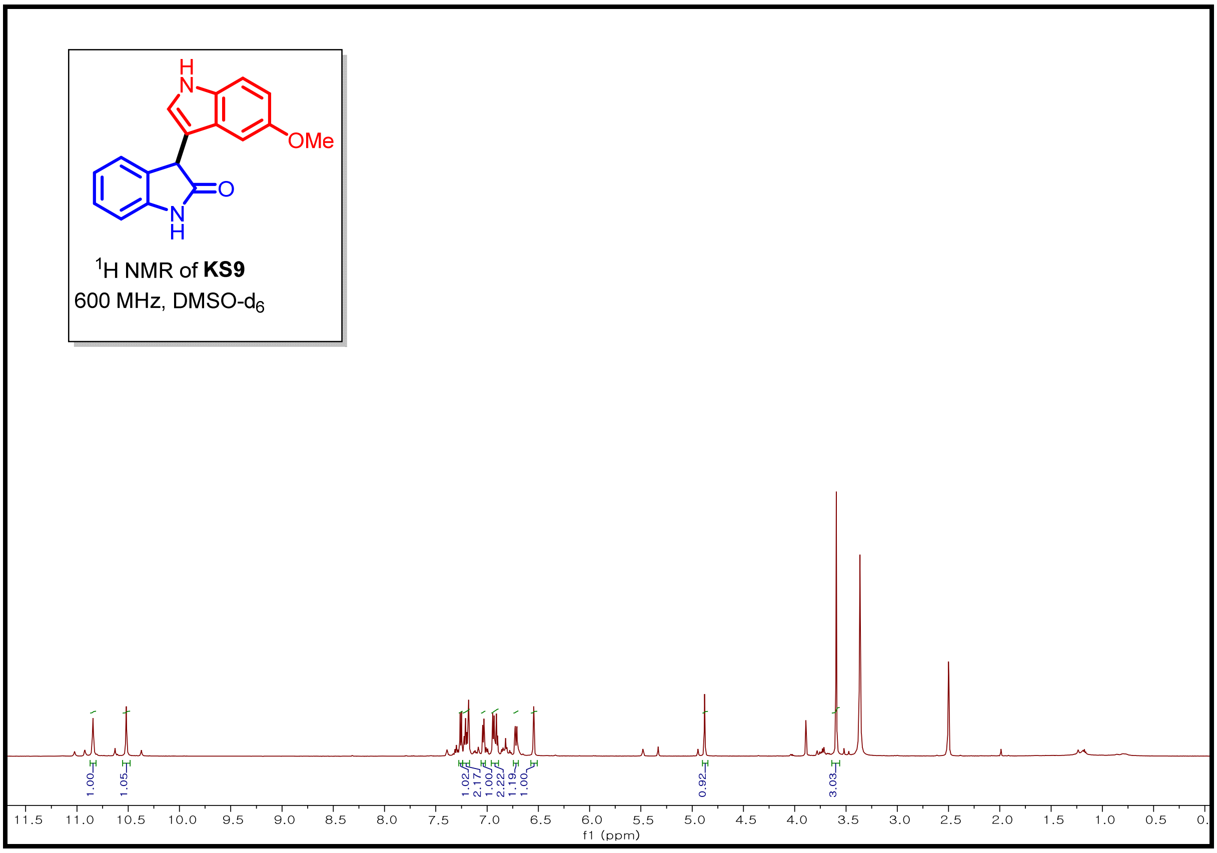

H
N
OMe
O
N
H
^{1}H NMR of **KS9**
600 MHz, DMSO-d$_6$
1.00
1.05
1.02
2.17
1.00
2.22
1.19
1.00
0.92
3.03
11.5 11.0 10.5 10.0 9.5 9.0 8.5 8.0 7.5 7.0 6.5 6.0 5.5 5.0 4.5 4.0 3.5 3.0 2.5 2.0 1.5 1.0 0.5 0.
f1 (ppm)

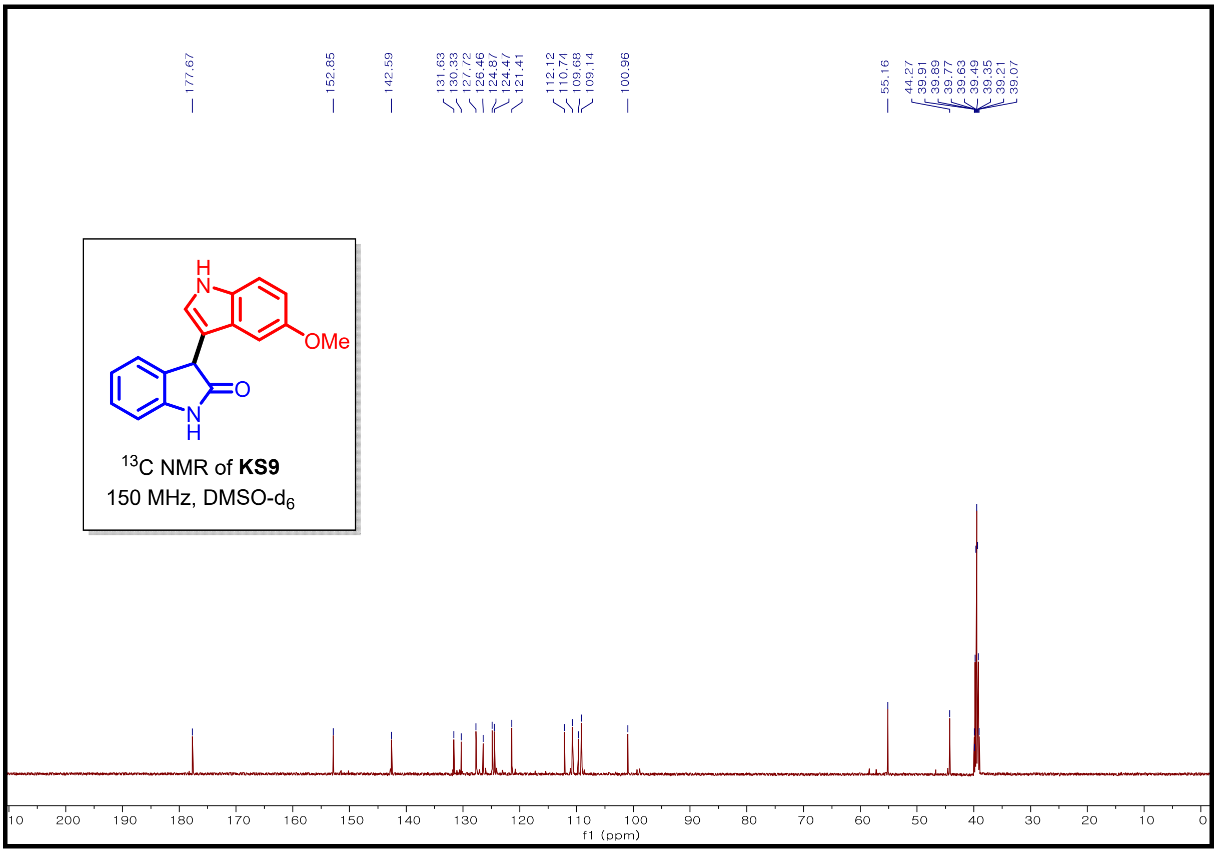

177.67
152.85
142.59
131.63
130.33
127.72
126.46
124.87
124.47
121.41
112.12
110.74
109.68
109.14
100.96
55.16
44.27
39.91
39.89
39.77
39.63
39.49
39.35
39.21
39.07
H
N
OMe
O
N
H
^{13}C NMR of **KS9**
150 MHz, DMSO-d$_6$
10 200 190 180 170 160 150 140 130 120 110 100 90 80 70 60 50 40 30 20 10 0
f1 (ppm)

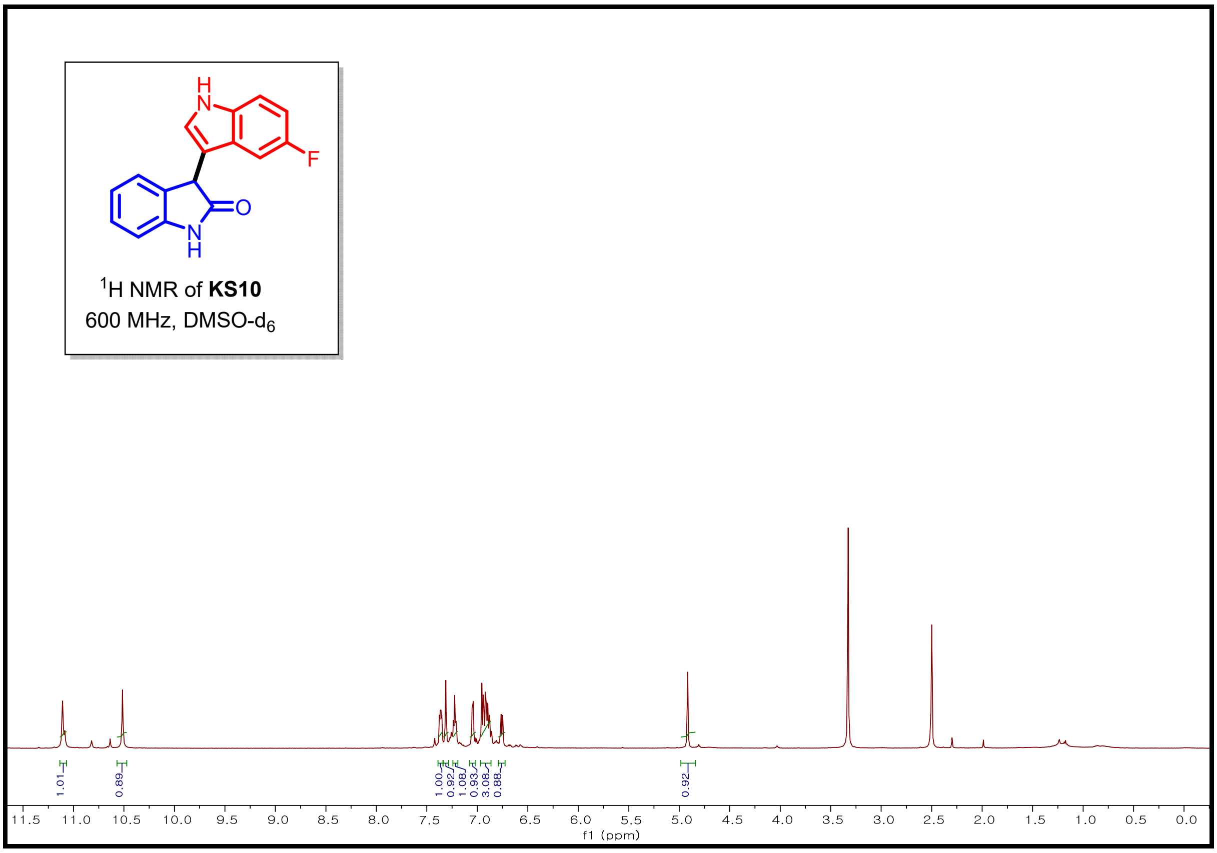

F
O
^{1}H NMR of **KS10**
600 MHz, DMSO-d$_6$
1.01
0.89
1.00
0.92
1.08
0.93
3.08
0.88
0.92
11.5 11.0 10.5 10.0 9.5 9.0 8.5 8.0 7.5 7.0 6.5 6.0 5.5 5.0 4.5 4.0 3.5 3.0 2.5 2.0 1.5 1.0 0.5 0.0
f1 (ppm)

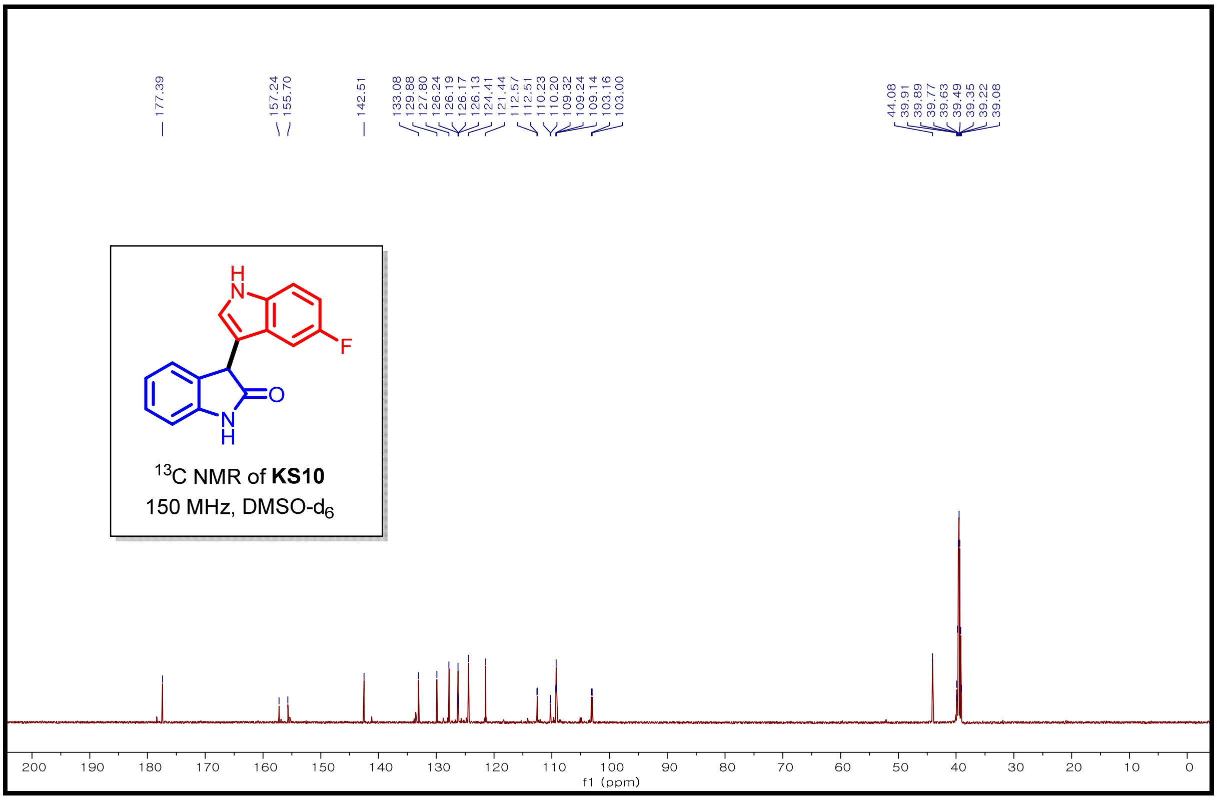

177.39
157.24
155.70
142.51
133.08
129.88
127.80
126.24
126.19
126.17
126.13
124.41
121.44
112.57
112.51
110.23
110.20
109.32
109.24
109.14
103.16
103.00
44.08
39.91
39.89
39.77
39.63
39.49
39.35
39.22
39.08
F
O
^{13}C NMR of **KS10**
150 MHz, DMSO-d$_6$
200 190 180 170 160 150 140 130 120 110 100 90 80 70 60 50 40 30 20 10 0
f1 (ppm)

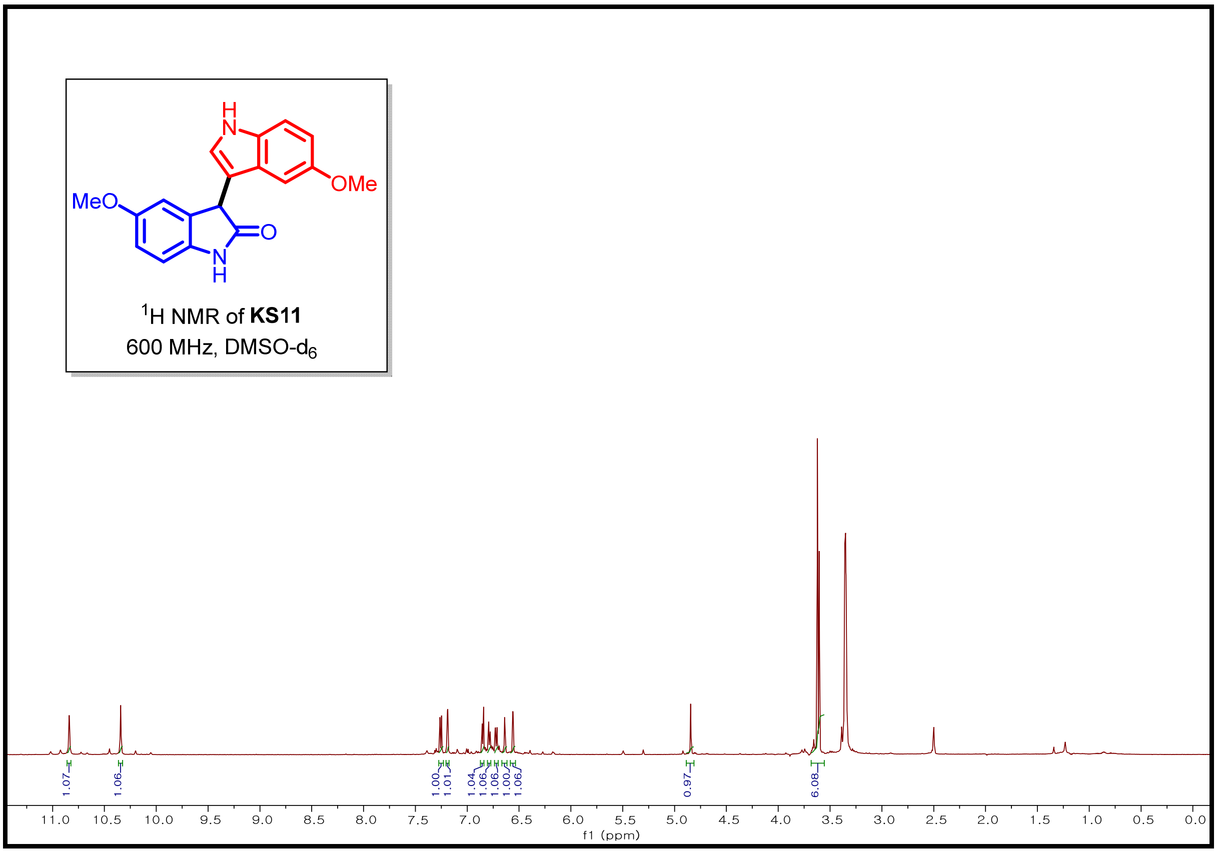
H
N
OMe
MeO
O
N
H
1H NMR of KS11
600 MHz, DMSO-d6
1.07
1.06
1.00
1.01
1.04
1.06
1.06
1.00
1.06
0.97
6.08
11.0 10.5 10.0 9.5 9.0 8.5 8.0 7.5 7.0 6.5 6.0 5.5 5.0 4.5 4.0 3.5 3.0 2.5 2.0 1.5 1.0 0.5 0.0
f1 (ppm)

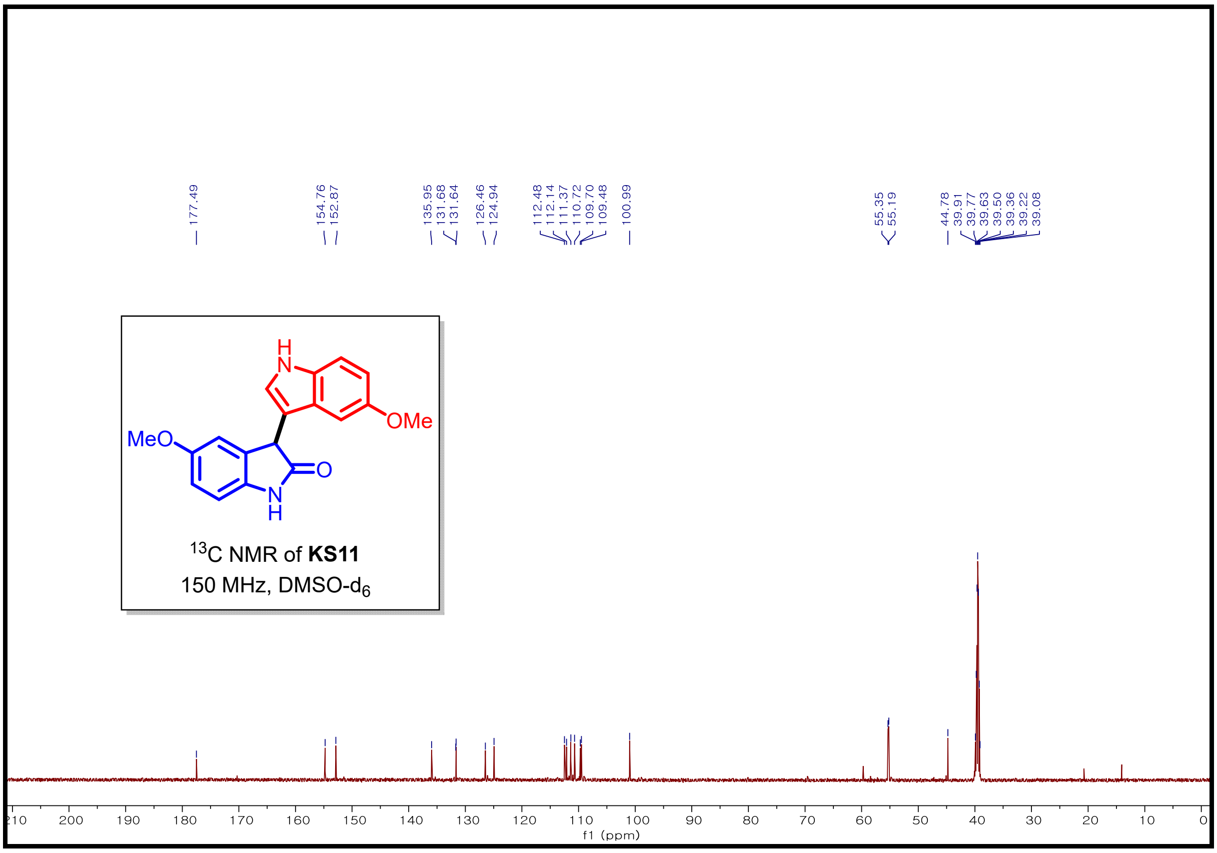
177.49
154.76
152.87
135.95
131.68
131.64
126.46
124.94
112.48
112.14
111.37
110.72
109.70
109.48
100.99
55.35
55.19
44.78
39.91
39.77
39.63
39.50
39.36
39.22
39.08
H
N
OMe
MeO
O
N
H
13C NMR of KS11
150 MHz, DMSO-d6
200 190 180 170 160 150 140 130 120 110 100 90 80 70 60 50 40 30 20 10 0
f1 (ppm)

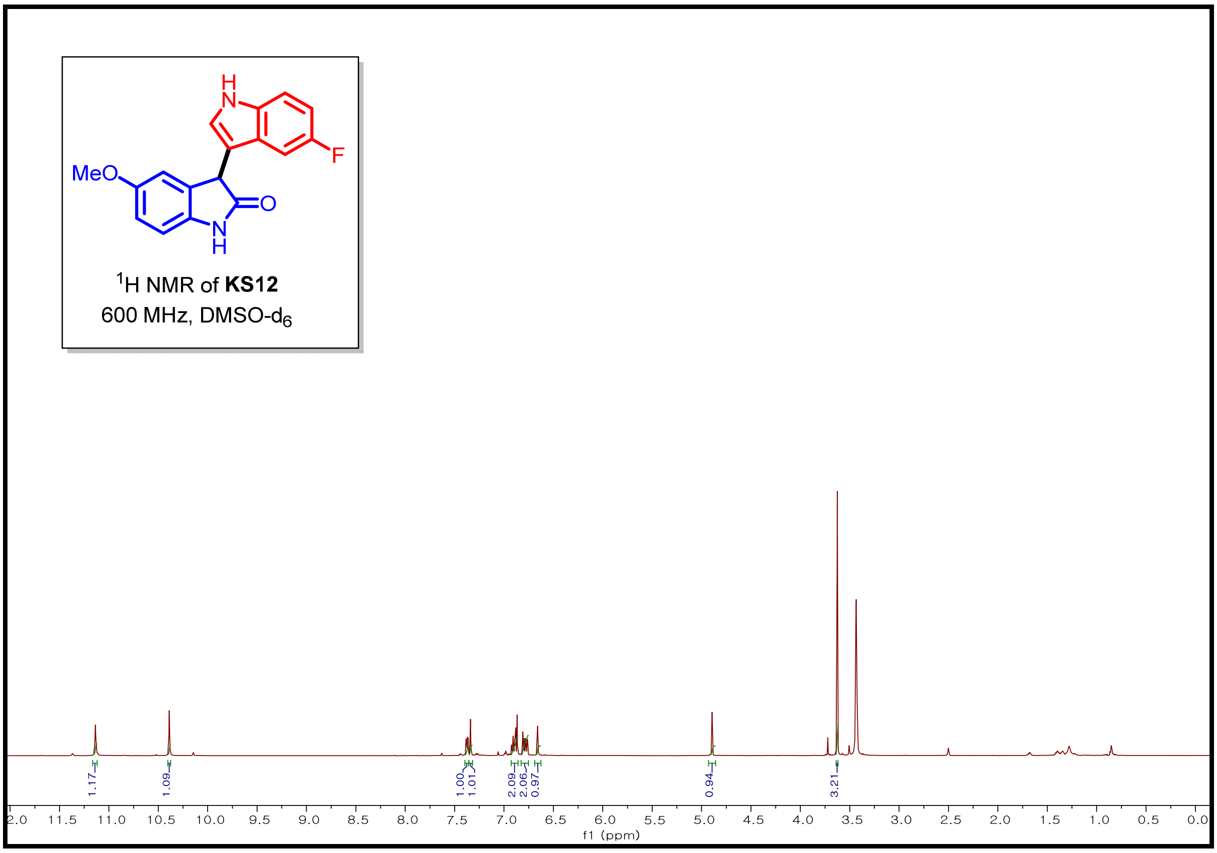
MeO
F
^{1}H NMR of **KS12**
600 MHz, DMSO-d$_6$
1.17
1.09
1.00
1.01
2.09
2.06
0.97
0.94
3.21
f1 (ppm)

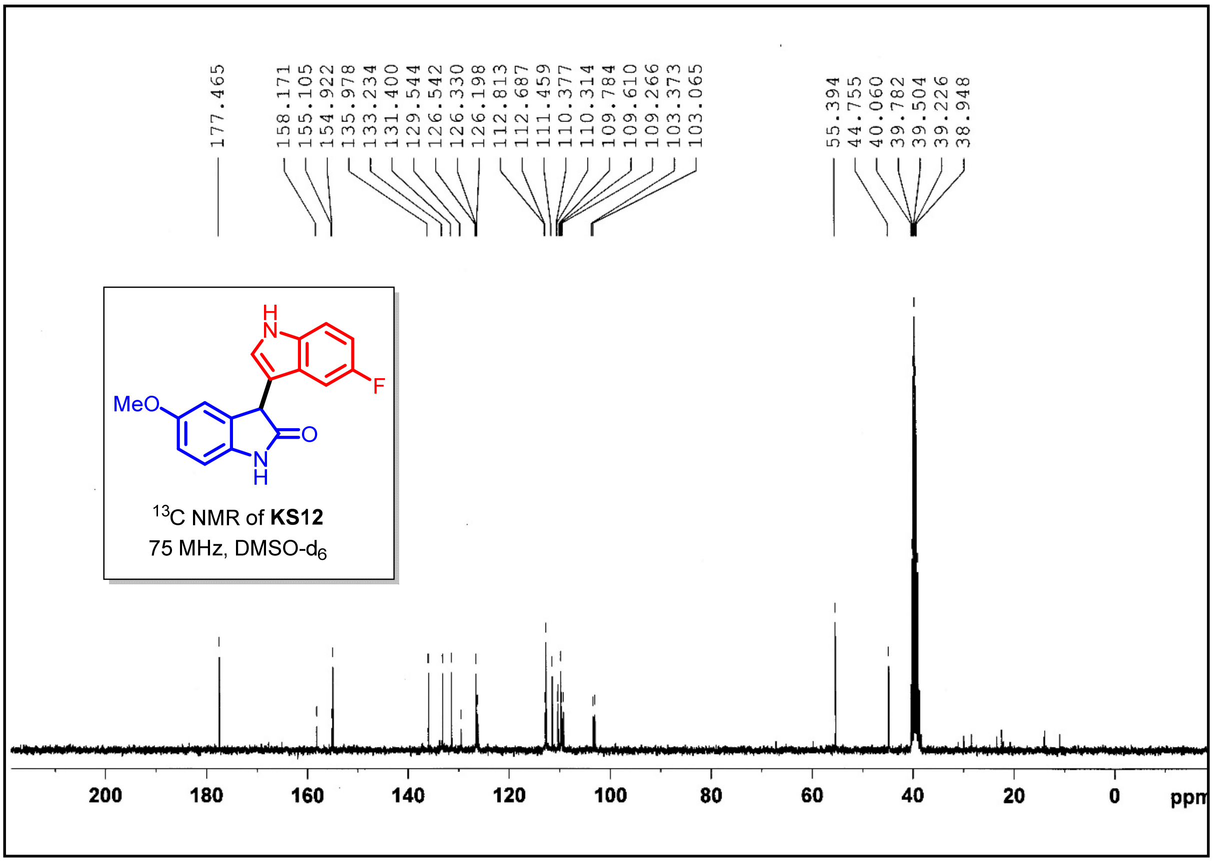
177.465
158.171
155.105
154.922
135.978
133.234
131.400
129.544
126.542
126.330
126.198
112.813
112.687
111.459
110.377
110.314
109.784
109.610
109.266
103.373
103.065
55.394
44.755
40.060
39.782
39.504
39.226
38.948
MeO
F
^{13}C NMR of **KS12**
75 MHz, DMSO-d$_6$
ppm

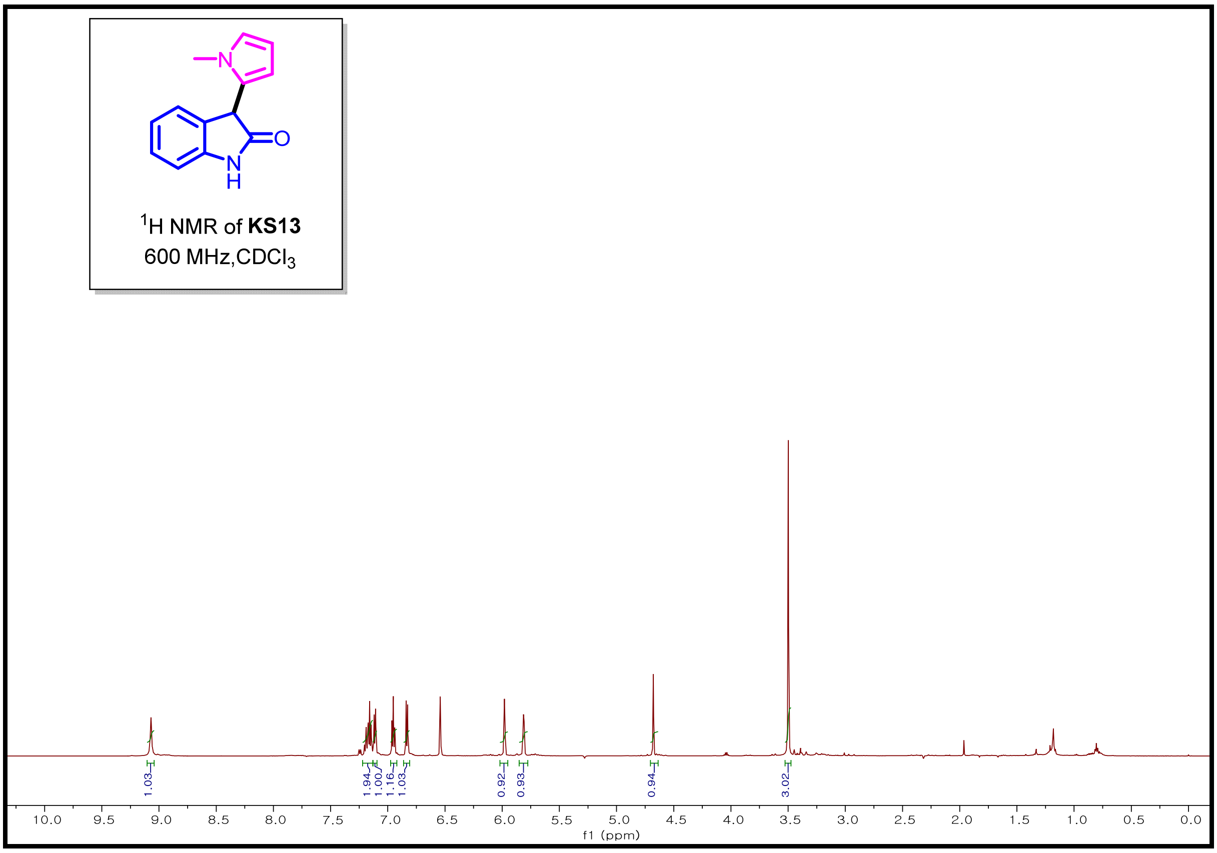
N
O
N
H
1H NMR of KS13
600 MHz,CDCl3
1.03
1.94
1.00
1.16
1.03
0.92
0.93
0.94
3.02
f1 (ppm)

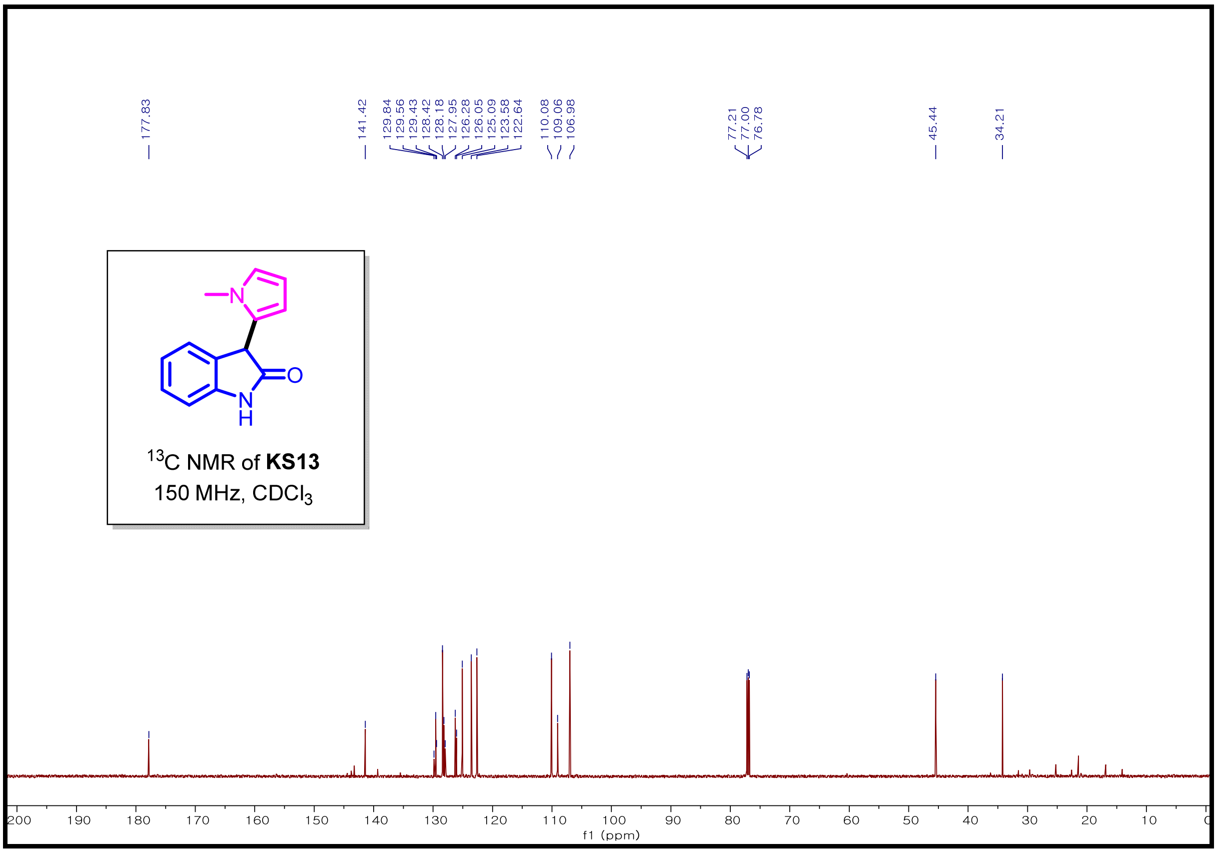
177.83
141.42
129.84
129.56
129.43
128.42
128.18
127.95
126.28
126.05
125.09
123.58
122.64
110.08
109.06
106.98
77.21
77.00
76.78
45.44
34.21
N
O
N
H
13C NMR of KS13
150 MHz, CDCl3
f1 (ppm)

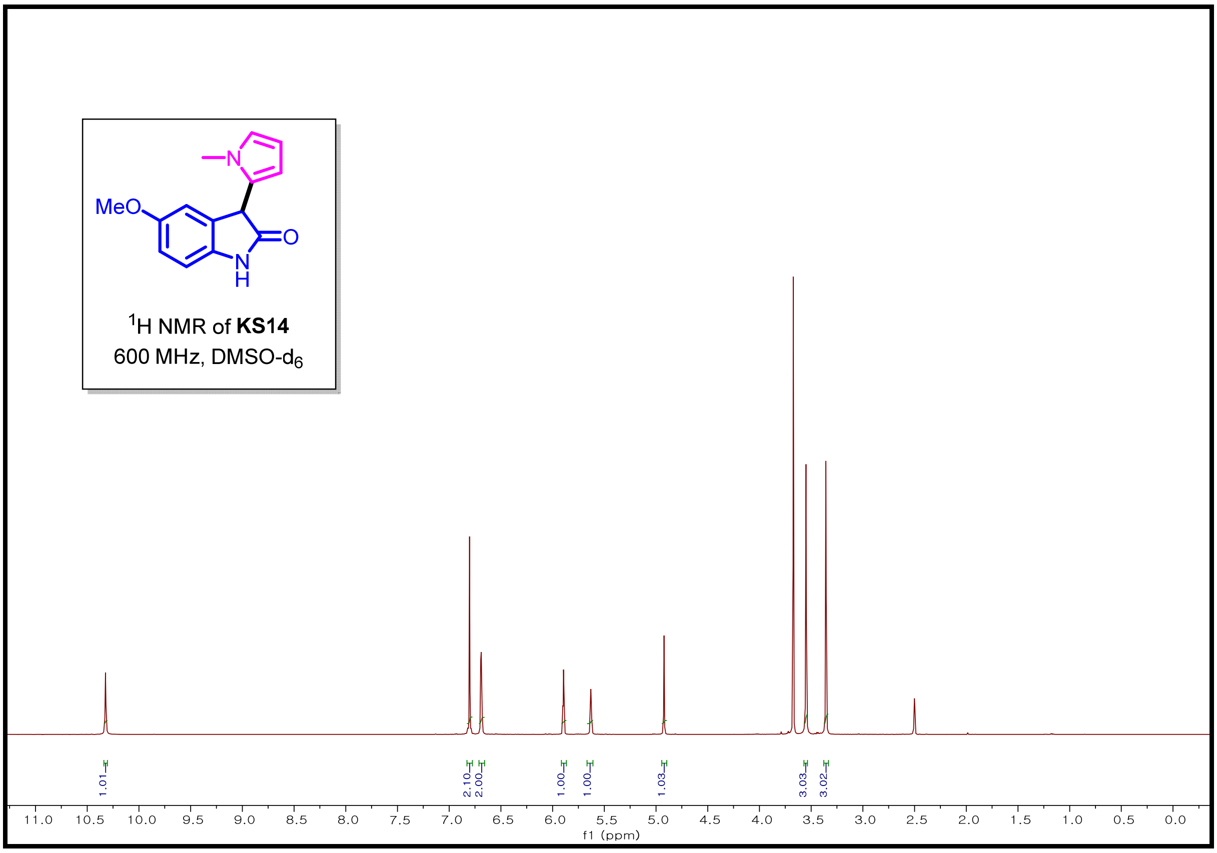

MeO
N
H
O
N
^{1}H NMR of **KS14**
600 MHz, DMSO-d$_6$
1.01
2.10
2.00
1.00
1.00
1.03
3.03
3.02
f1 (ppm)

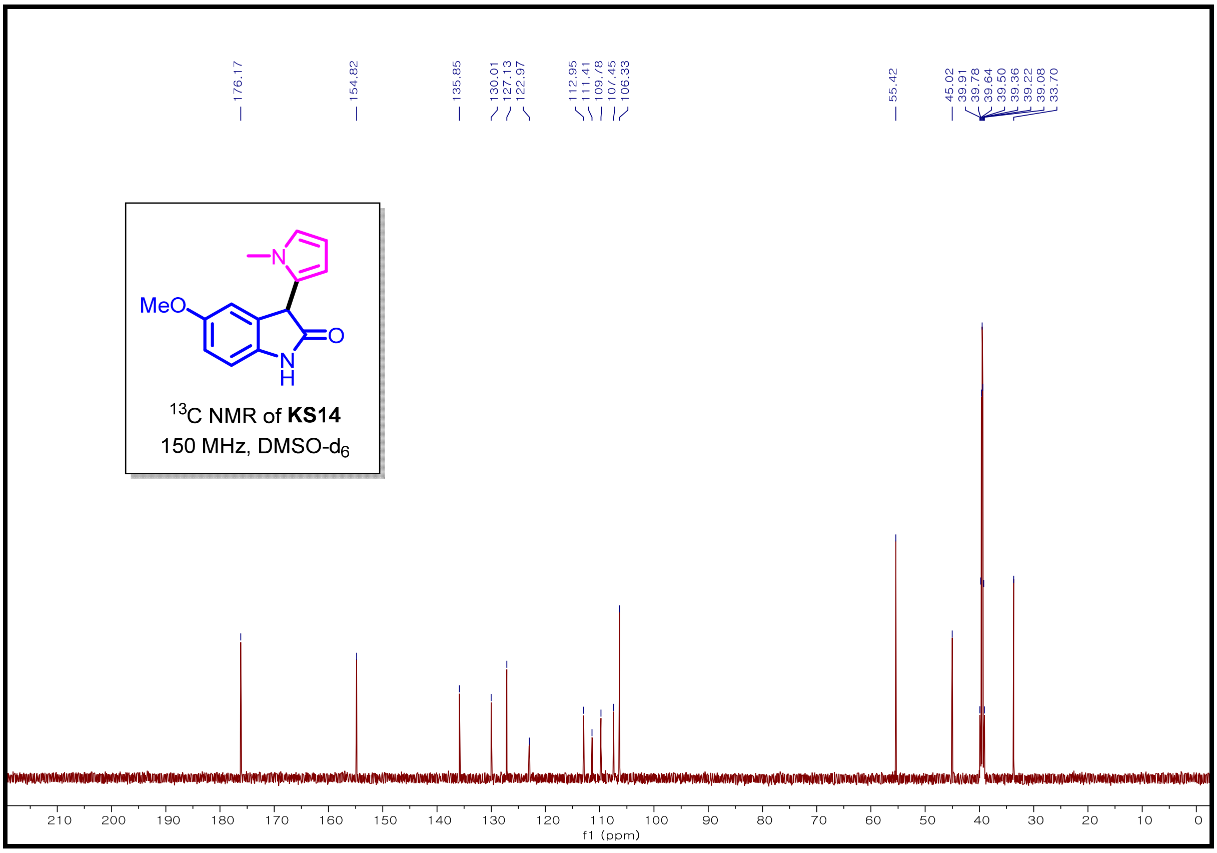

176.17
154.82
135.85
130.01
127.13
122.97
112.95
111.41
109.78
107.45
106.33
55.42
45.02
39.91
39.78
39.64
39.50
39.36
39.22
39.08
33.70
MeO
N
H
O
N
^{13}C NMR of **KS14**
150 MHz, DMSO-d$_6$
f1 (ppm)

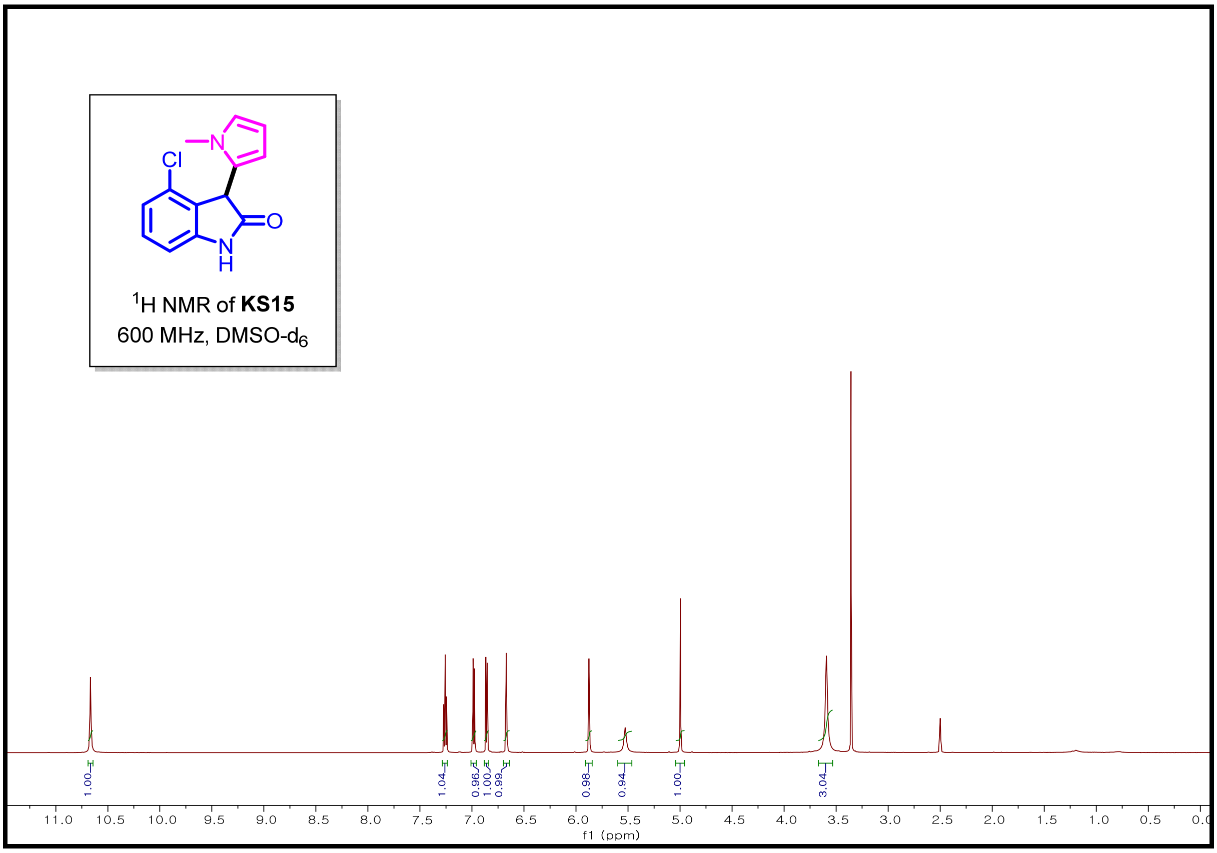
Cl
N
N
H
O
1H NMR of KS15
600 MHz, DMSO-d6
1.00
1.04
0.96
1.00
0.99
0.98
0.94
1.00
3.04
11.0 10.5 10.0 9.5 9.0 8.5 8.0 7.5 7.0 6.5 6.0 5.5 5.0 4.5 4.0 3.5 3.0 2.5 2.0 1.5 1.0 0.5 0.0
f1 (ppm)

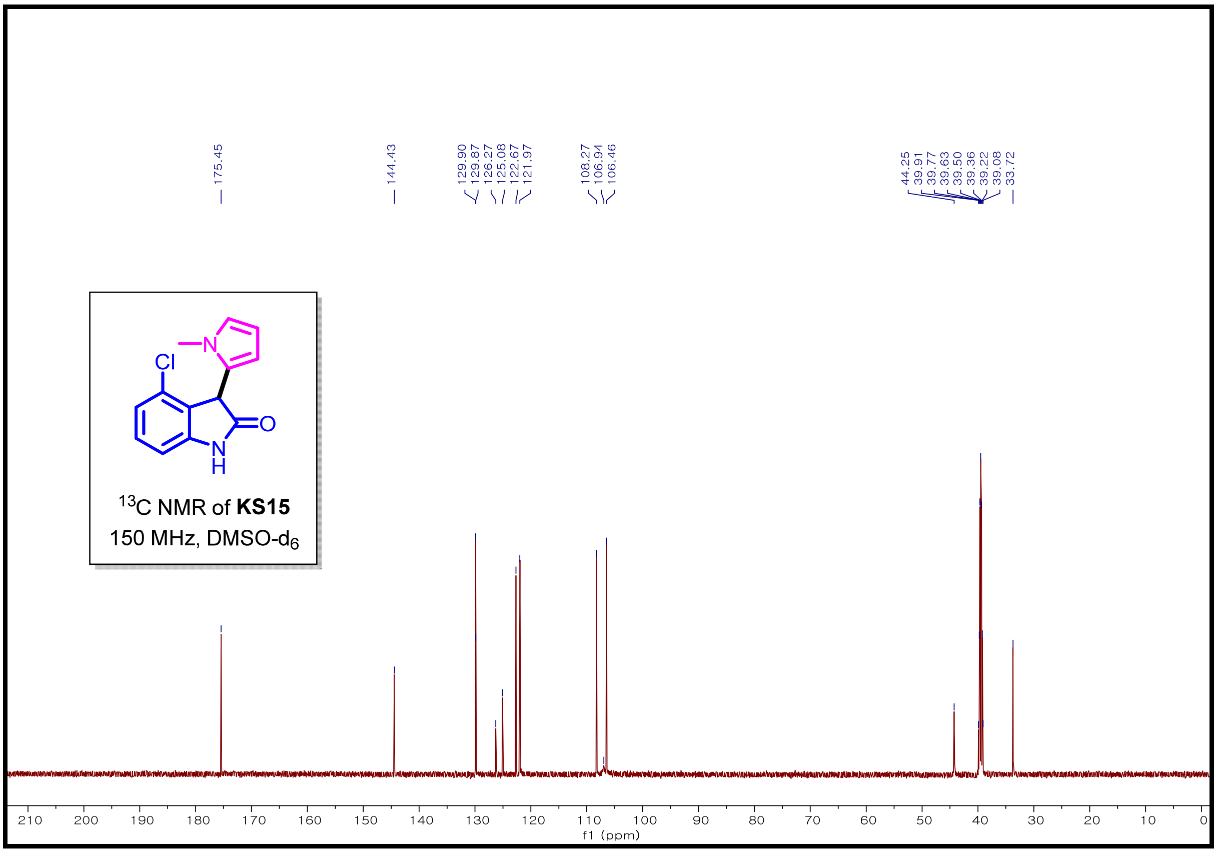
175.45
144.43
129.90
129.87
126.27
125.08
122.67
121.97
108.27
106.94
106.46
44.25
39.91
39.77
39.63
39.50
39.36
39.22
39.08
33.72
Cl
N
N
H
O
13C NMR of KS15
150 MHz, DMSO-d6
210 200 190 180 170 160 150 140 130 120 110 100 90 80 70 60 50 40 30 20 10 0
f1 (ppm)

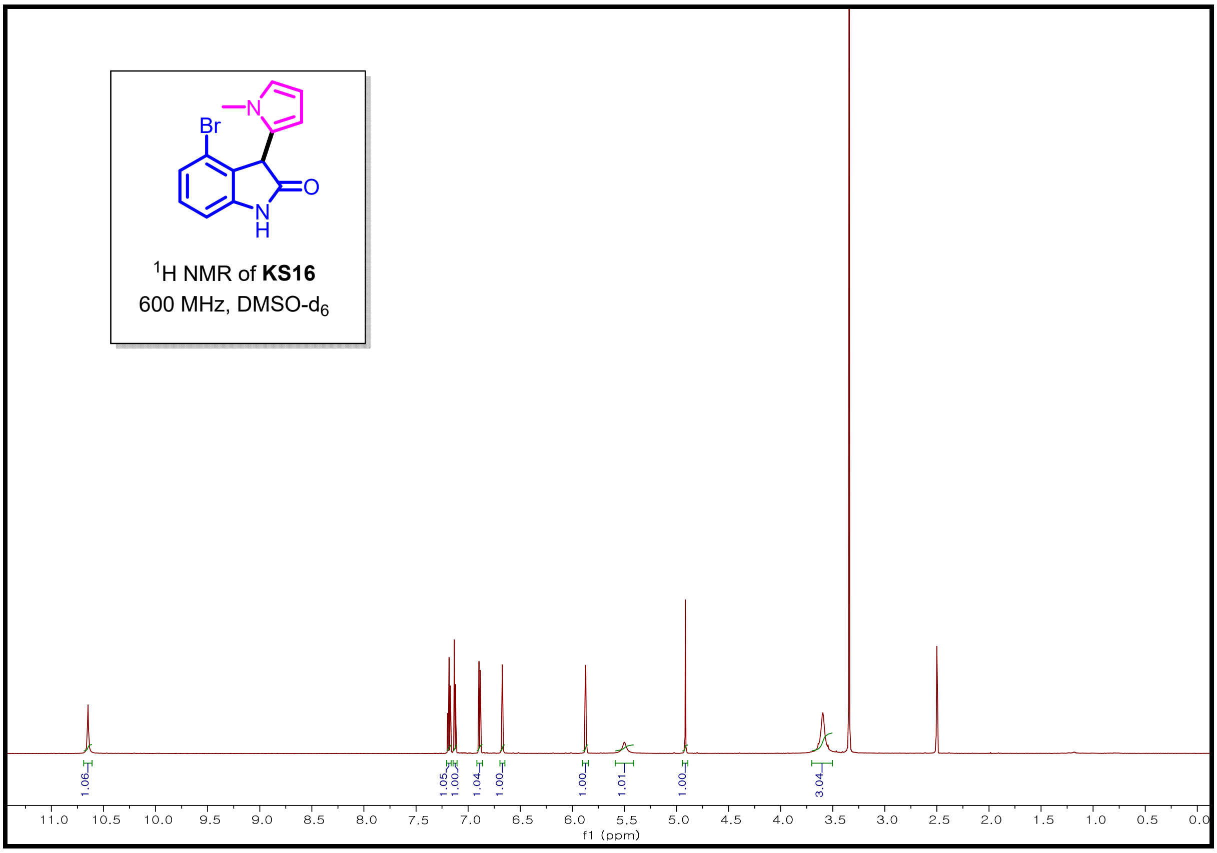
Br
N
N
H
O
1H NMR of KS16
600 MHz, DMSO-d6
1.06
1.05
1.00
1.04
1.00
1.00
1.01
1.00
3.04
f1 (ppm)

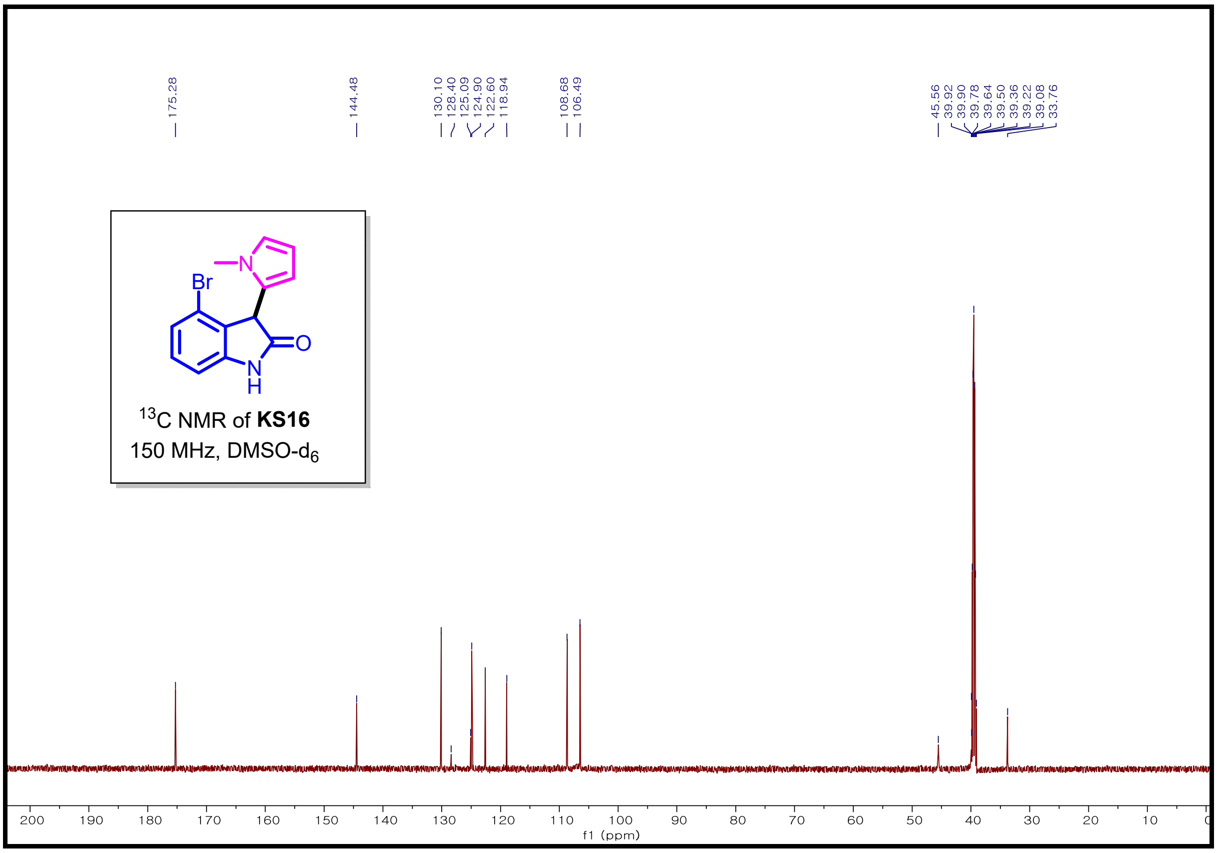
175.28
144.48
130.10
128.40
125.09
124.90
122.60
118.94
108.68
106.49
45.56
39.92
39.90
39.78
39.64
39.50
39.36
39.22
39.08
33.76
Br
N
N
H
O
13C NMR of KS16
150 MHz, DMSO-d6
f1 (ppm)

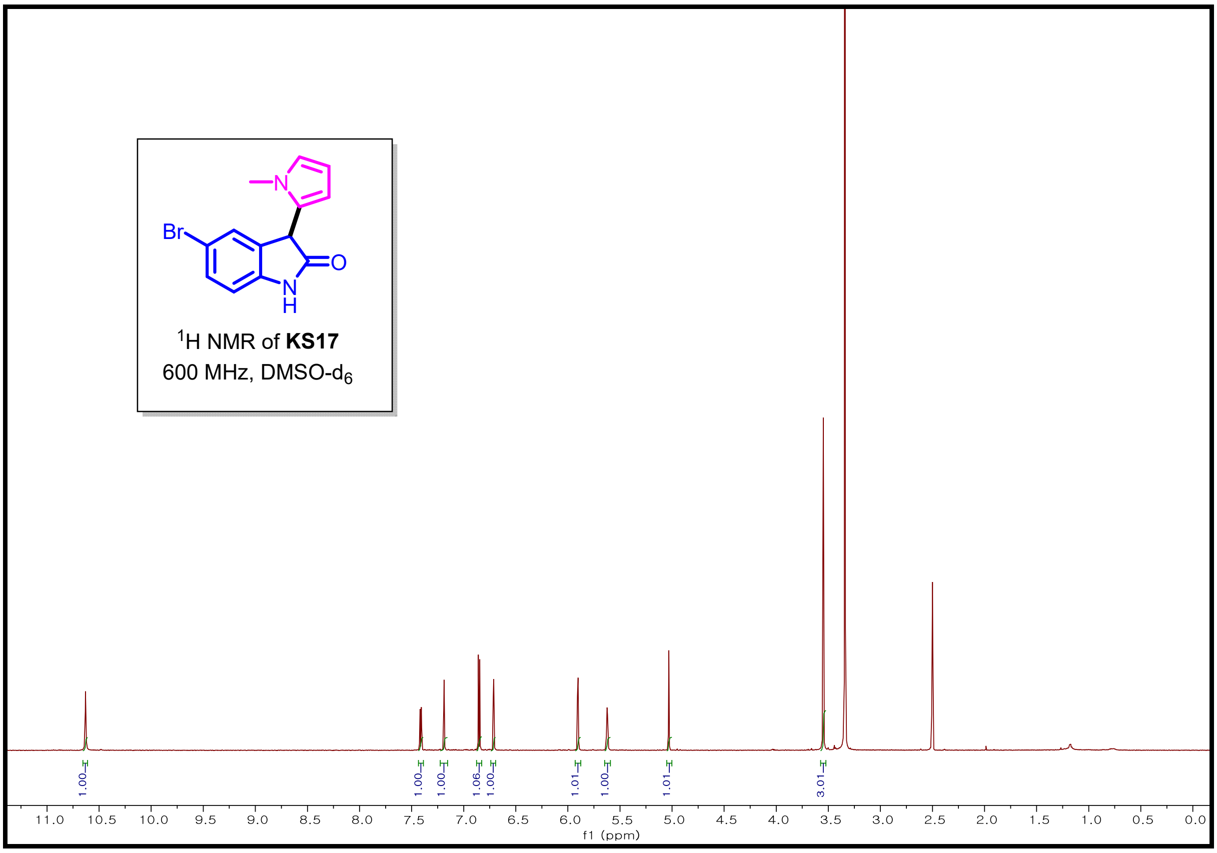
Br
N
N
H
O
^{1}H NMR of **KS17**
600 MHz, DMSO-d$_6$
1.00
1.00
1.00
1.06
1.00
1.01
1.00
1.01
3.01
f1 (ppm)

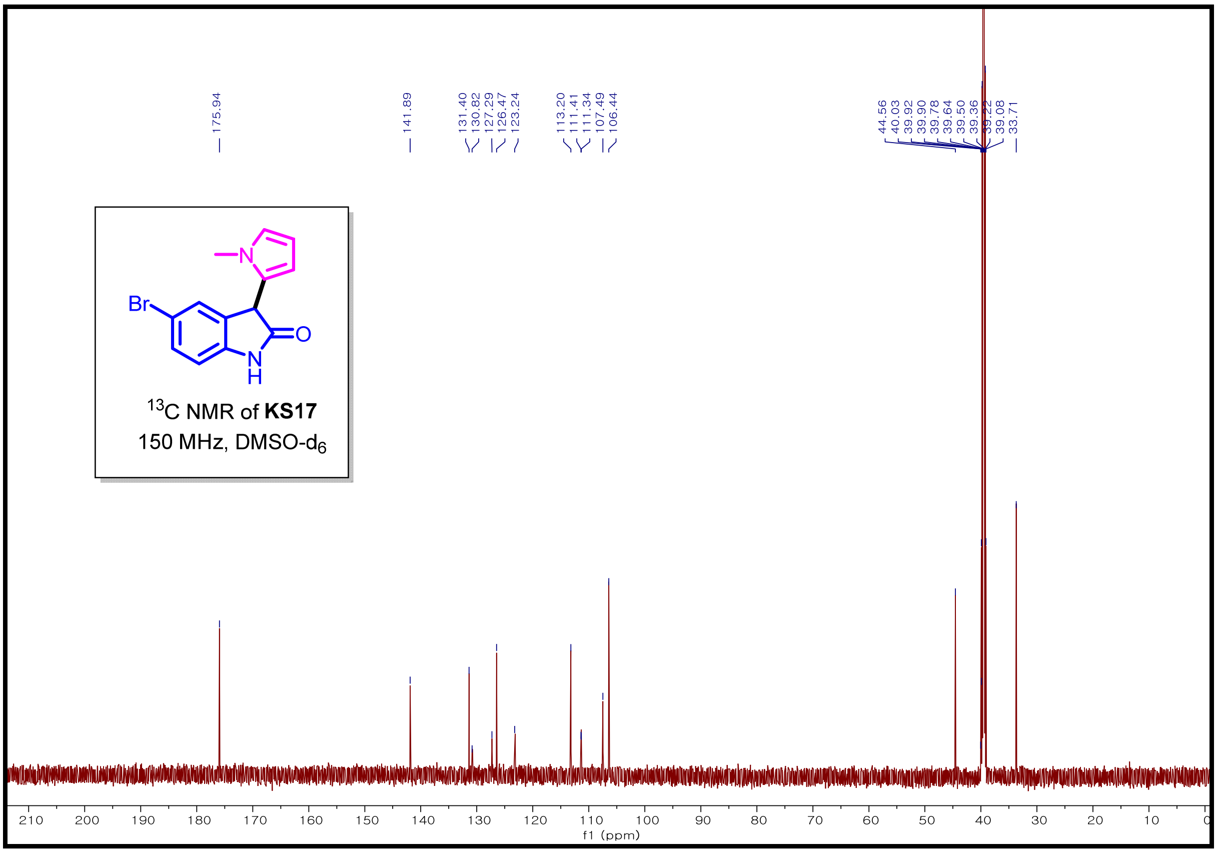
175.94
141.89
131.40
130.82
127.29
126.47
123.24
113.20
111.41
111.34
107.49
106.44
44.56
40.03
39.92
39.90
39.78
39.64
39.50
39.36
39.22
39.08
33.71
Br
N
N
H
O
^{13}C NMR of **KS17**
150 MHz, DMSO-d$_6$
f1 (ppm)

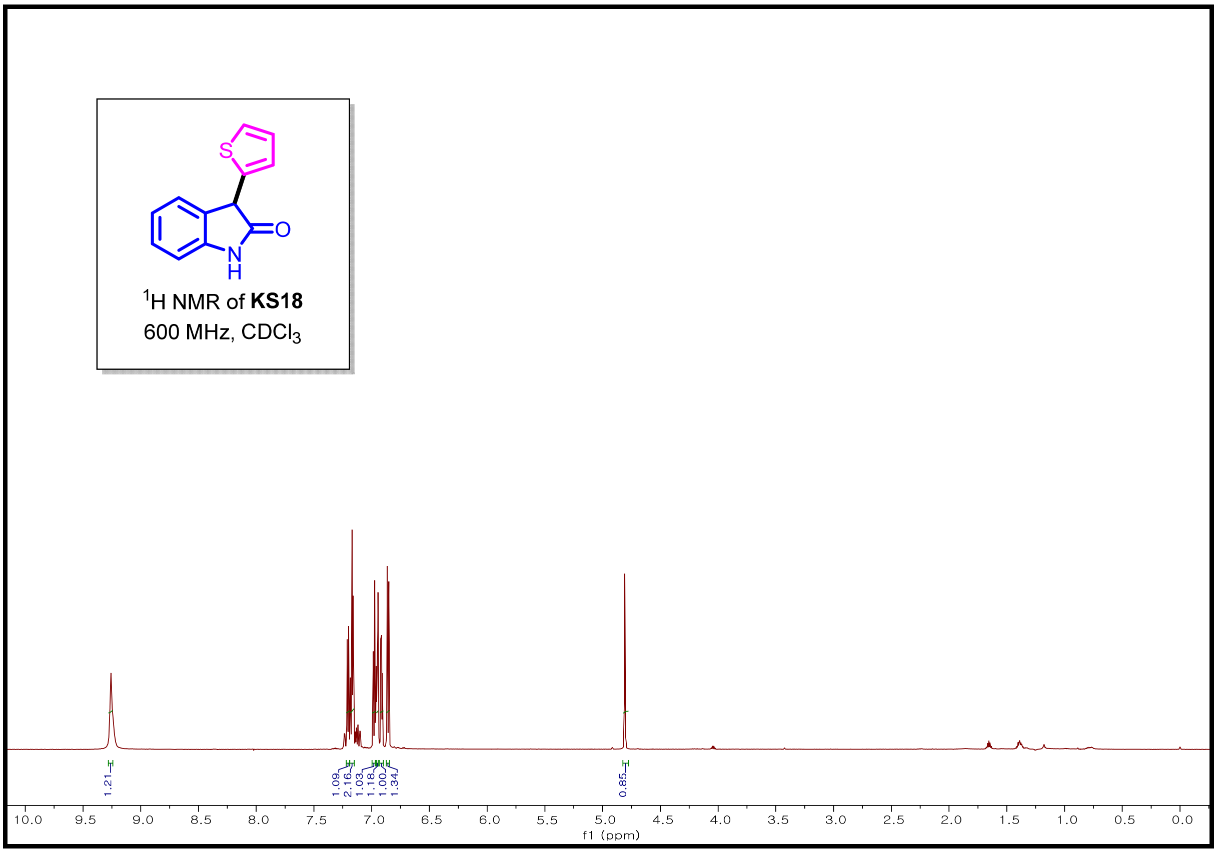
S
N
H
O
1H NMR of KS18
600 MHz, CDCl3
1.21
1.09
2.16
1.03
1.18
1.00
1.34
0.85
10.0 9.5 9.0 8.5 8.0 7.5 7.0 6.5 6.0 5.5 5.0 4.5 4.0 3.5 3.0 2.5 2.0 1.5 1.0 0.5 0.0
f1 (ppm)

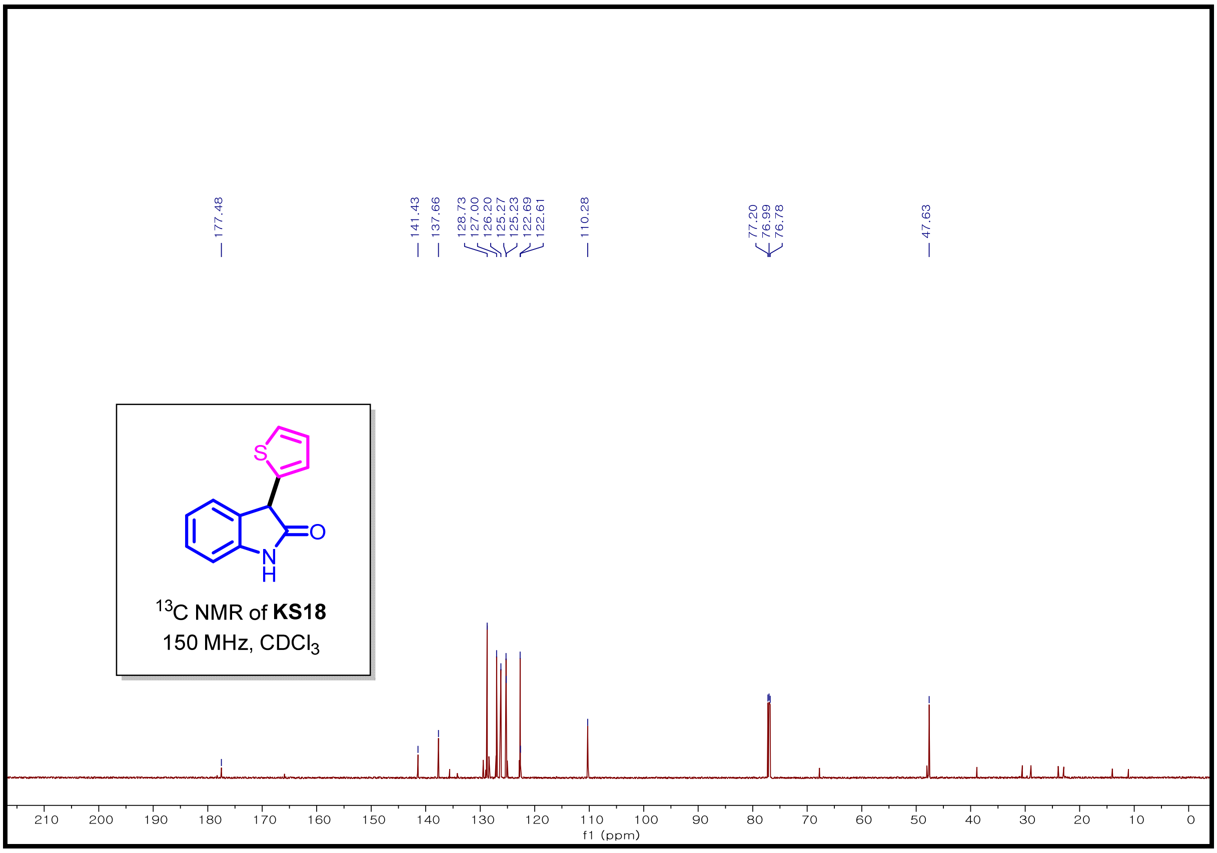
177.48
141.43
137.66
128.73
127.00
126.20
125.27
125.23
122.69
122.61
110.28
77.20
76.99
76.78
47.63
S
N
H
O
13C NMR of KS18
150 MHz, CDCl3
210 200 190 180 170 160 150 140 130 120 110 100 90 80 70 60 50 40 30 20 10 0
f1 (ppm)

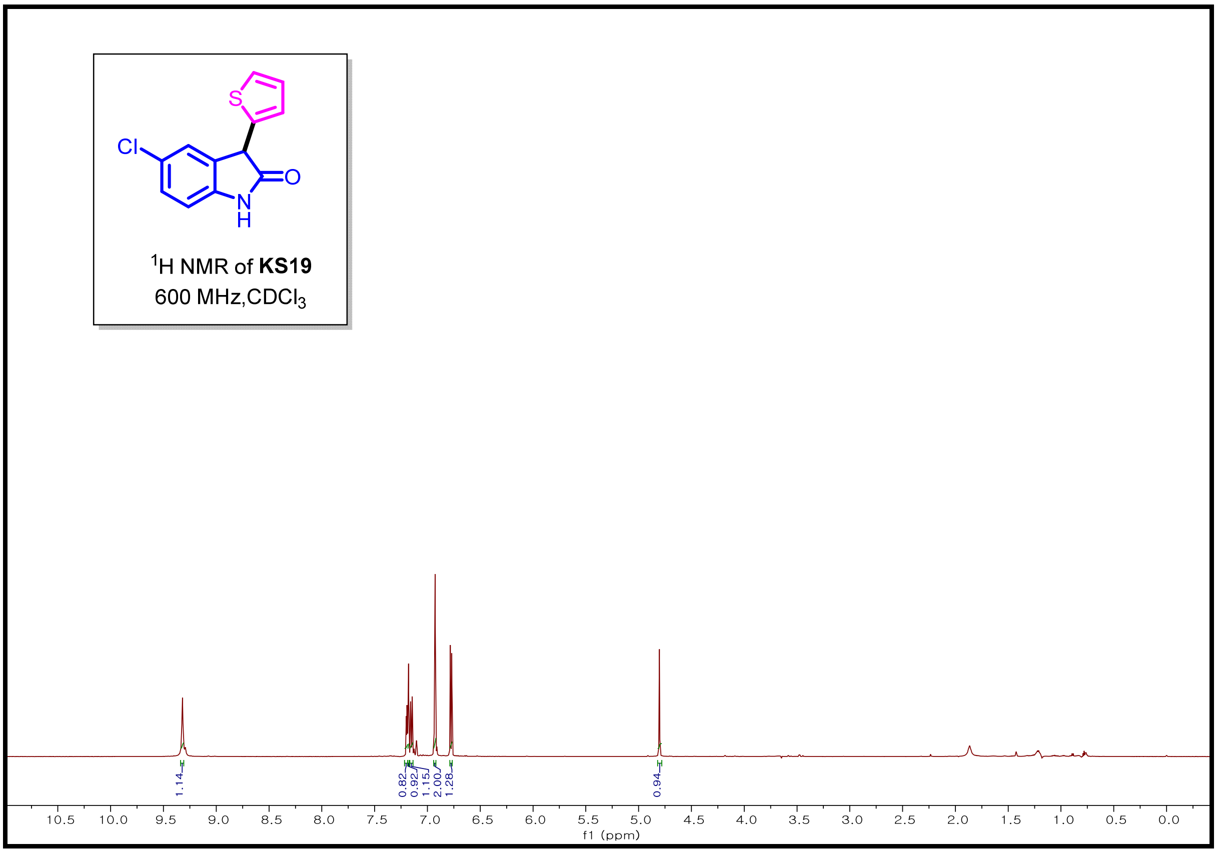
Cl
S
O
N
H
1H NMR of KS19
600 MHz,CDCl3
1.14
0.82
0.92
1.15
2.00
1.28
0.94
10.5 10.0 9.5 9.0 8.5 8.0 7.5 7.0 6.5 6.0 5.5 5.0 4.5 4.0 3.5 3.0 2.5 2.0 1.5 1.0 0.5 0.0
f1 (ppm)

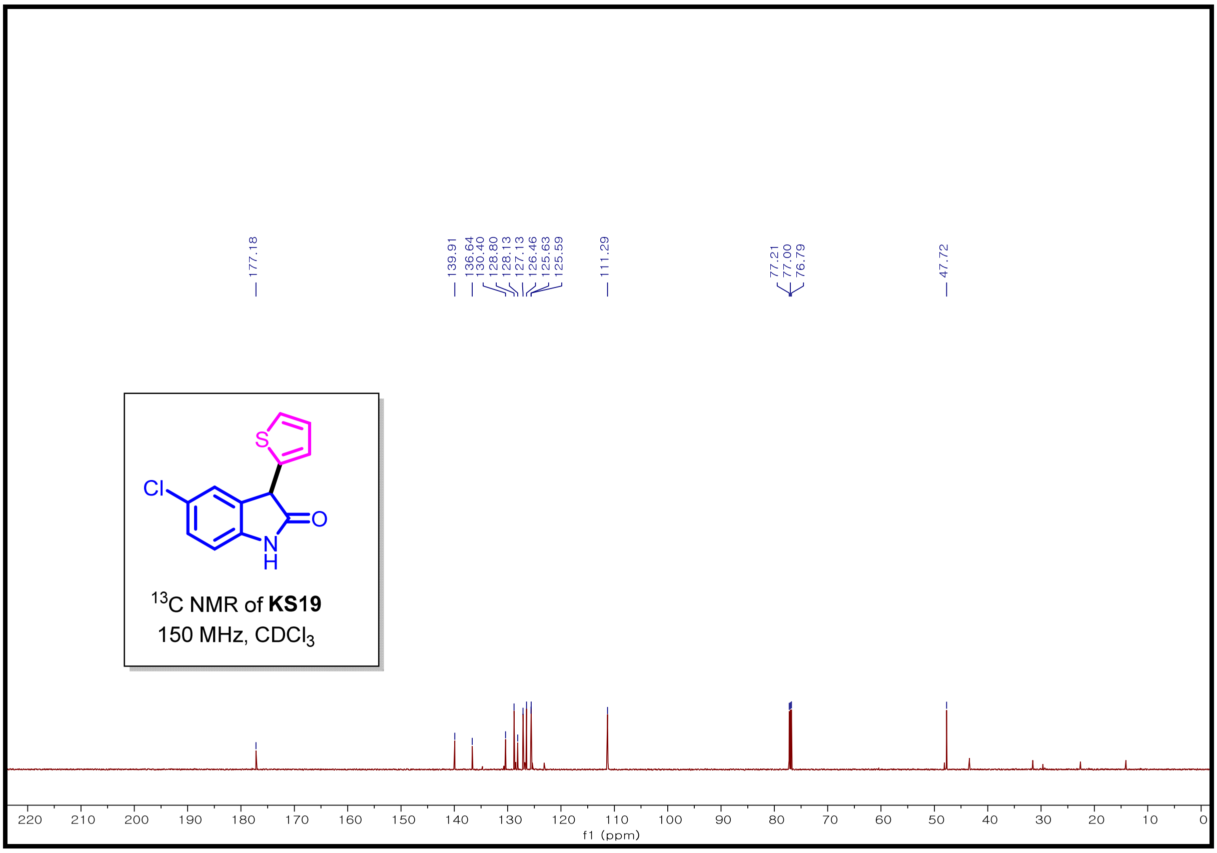
177.18
139.91
136.64
130.40
128.80
128.13
127.13
126.46
125.63
125.59
111.29
77.21
77.00
76.79
47.72
Cl
S
O
N
H
13C NMR of KS19
150 MHz, CDCl3
220 210 200 190 180 170 160 150 140 130 120 110 100 90 80 70 60 50 40 30 20 10 0
f1 (ppm)

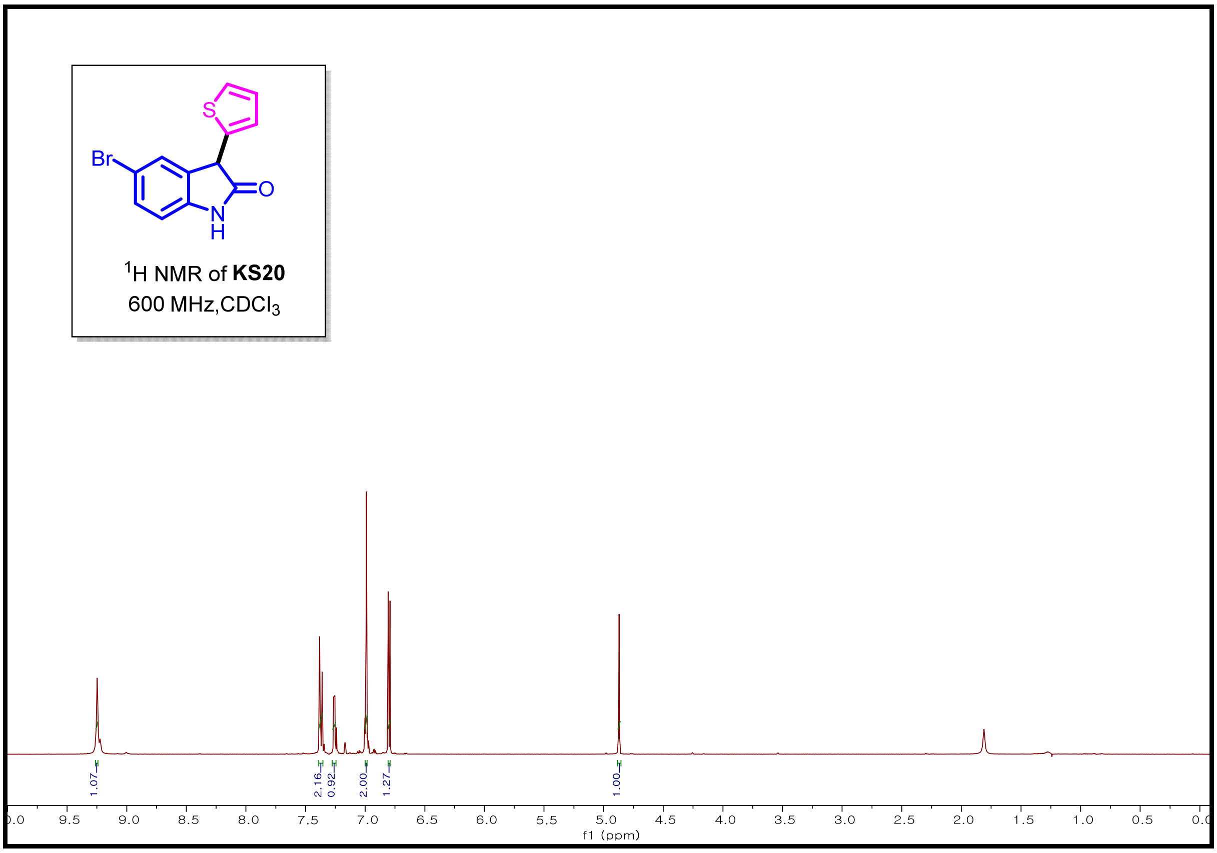
Br
S
O
N
H
1H NMR of KS20
600 MHz,CDCl3
1.07
2.16
0.92
2.00
1.27
1.00
f1 (ppm)

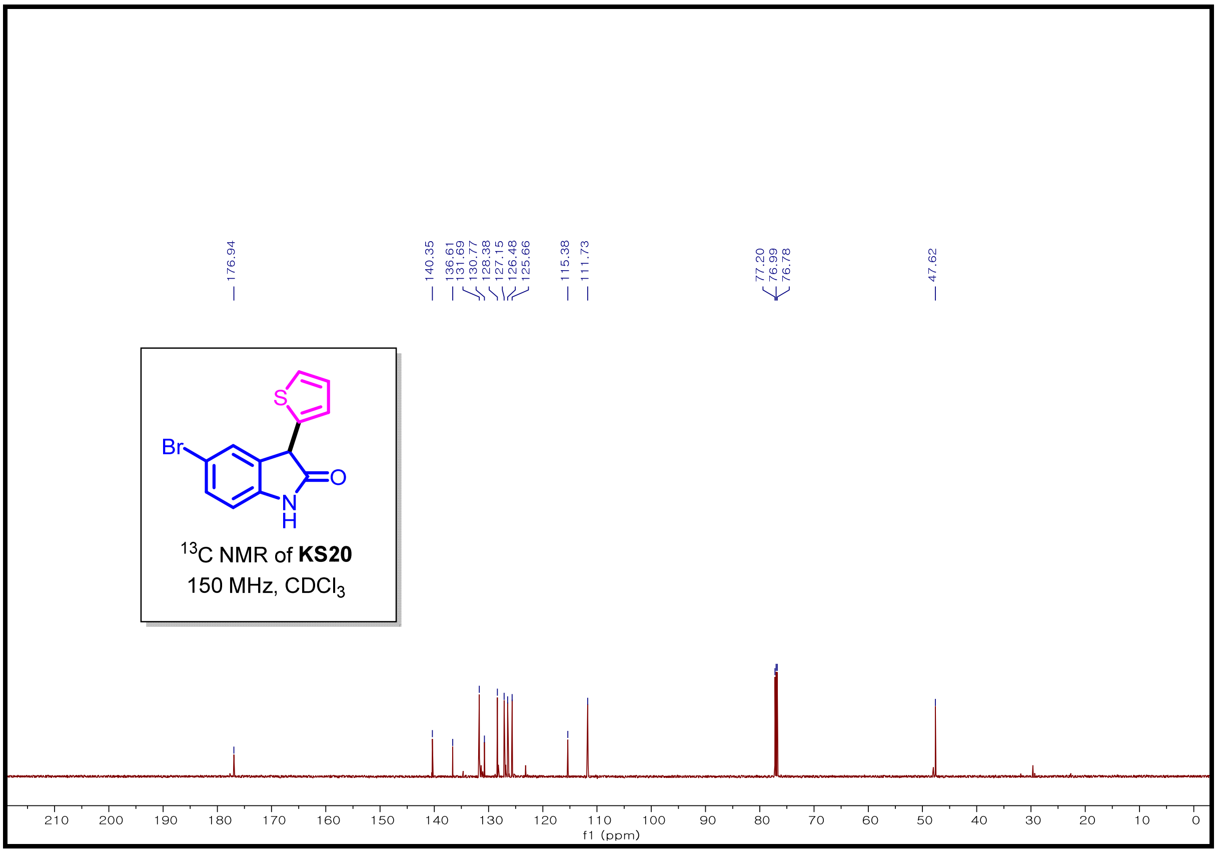
176.94
140.35
136.61
131.69
130.77
128.38
127.15
126.48
125.66
115.38
111.73
77.20
76.99
76.78
47.62
Br
S
O
N
H
13C NMR of KS20
150 MHz, CDCl3
f1 (ppm)

Copy of GC Spectrum of compound KS16

[TIC]
Data : HDK-1 — Date : 11-Apr-2019 09:10
Sample: -
Note : -
Inlet : GC — Ion Mode : EI+
Ion Species : Normal Ion — TIC Range : m/z 10 to 500

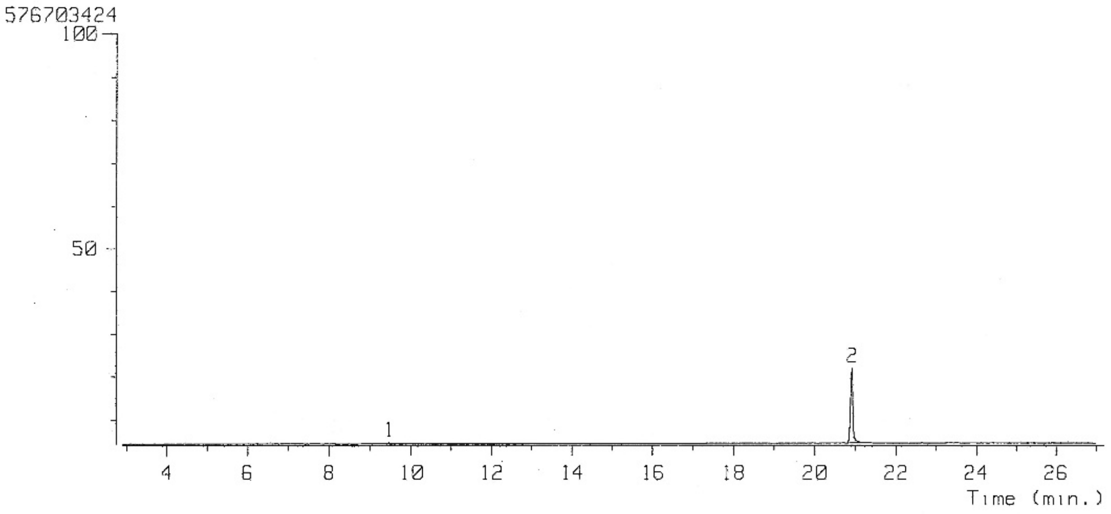

No.	RT[min]	Area	Area%	Height	Height%	Width[sec]	INTEG
1	9.46	235.44	0.59	172.48	1.78	1.28	BB
2	20.92	39537.68	99.41	9519.67	98.22	3.90	BB

Figure S1. Gas chromatogram of the compound **KS16**.
